# Burden of mortality attributable to diurnal temperature range in Thailand: a nationwide case-crossover analysis from 2007 to 2021

**DOI:** 10.1186/s41182-025-00761-1

**Published:** 2025-06-03

**Authors:** Chittamon Sritong-aon, Arthit Phosri, Tanasri Sihabut, Tawach Prechthai

**Affiliations:** 1 Department of Environmental Health Sciences, Faculty of Public Health, Mahidol University, Bangkok, Thailand; 2 Center of Excellence On Environmental Health and Toxicology (EHT), Office of the Permanent Secretary (OPS), Ministry of Higher Education, Science, Research and Innovation (MHESI), Bangkok, Thailand

**Keywords:** Diurnal temperature range, Mortality, Attributable risk, Climate change, Thailand

## Abstract

**Introduction:**

Diurnal temperature range (DTR), the difference between daily maximum and minimum temperatures, has been increasingly recognized for its potential impact on human health. However, its contribution to mortality remains underexplored, particularly in tropical regions such as Thailand.

**Objective:**

To estimate the burden of all-cause mortality attributable to variations in DTR in Thailand utilizing data from 2007 to 2021.

**Methodology:**

Data on daily all-cause mortality (ICD-10: A00–R99), excluding accidental causes, were obtained from the Strategy and Planning Division under the Office of the Permanent Secretary, Ministry of Public Health between January 2007 and December 2021, while daily meteorological data were sourced from the Thai Meteorological Department during the same period. A two-stage statistical model was utilized to assess the relationship between DTR and mortality. In the first stage, a time-stratified case-crossover design with conditional Poisson regression model was applied to estimate the province-specific associations between DTR and mortality. In the second stage, these province-specific estimates were pooled using a multivariate meta-regression model to obtain the national-level estimate. Finally, the mortality burden attributable to variations in DTR was determined using a backward perspective based on the relative risks obtained from the distributed lag non-linear model (DLNM).

**Results:**

During the study period, a total of 5,574,850 non-accidental cause of deaths was reported. The association between DTR and mortality followed a non-linear with U-shaped pattern, where the effect of DTR on mortality was higher at both low and high DTR levels. The fraction of mortality attributable to DTR at cumulative lag 0–7, 0–14, and 0–21 days was 1.88% (95% empirical confidence interval (eCI): 0.69–3.03), 2.39% (95% eCI: 0.75–3.99), and 4.67% (95% eCI: − 1.14–9.87), respectively.

**Conclusions:**

The findings indicate that both low and high DTRs were associated with an increased risk of all-cause mortality in Thailand. This underscores the need to consider DTR as a significant climate-related health risk, particularly in tropical regions, to inform public health strategies aimed at reducing the burden of climate-related mortality.

**Supplementary Information:**

The online version contains supplementary material available at 10.1186/s41182-025-00761-1.

## Introduction

Climate change is increasingly recognized as a major public health problem, with temperature extremes playing a pivotal role in shaping health outcomes worldwide [1–4]. Temperature extremes have long been recognized as a critical factor influencing adverse health outcomes [1, 5, 6], with both high and low temperatures being associated with an increased risk of mortality in many different settings [7–10]. While much attention has been paid to the impact of heatwave and cold spell, diurnal temperature range (DTR), which is the difference between daytime maximum and nighttime minimum temperatures, has emerged as an important climate variable in health research [11], yet it is often overlooked. Recent studies suggest that large DTR may exacerbate health risks, as the ability of body to adapt to temperature fluctuations is limited, particularly for vulnerable groups of populations such as the elderly, individuals with pre-existing health conditions, and those living in socioeconomically disadvantaged areas [12–15]. Diurnal temperature fluctuations have been associated with various health outcomes, including cardiovascular and respiratory diseases, as well as exacerbation of chronic conditions [14, 16]. While existing research has demonstrated the association between extreme temperatures and mortality in many regions [1, 2], few studies have focused specifically on the effects of DTR in tropical countries [14, 17–19].

Thailand, located in Southeast Asia, experiences a tropical climate characterized by significant seasonal variation, where climate variability is substantial [20, 21]. In particular, Thailand experiences a tropical monsoon climate characterized by three distinct seasons, which are a hot season (March–June), a rainy season (July–October), and a cool season (November–February). During hot season, daily maximum temperatures can reach up to 42 °C in some areas, while the cooler months typically see temperatures ranging from 20 °C to 30 °C. The rainy season, influenced by the monsoon, brings increased humidity and cloud cover, which moderates daily temperature fluctuations. These seasonal temperature variations significantly influence local ecosystems and human activities. While both intra-day (DTR) and inter-day temperature variation are important for understanding local climate patterns, this study specifically focuses on DTR. The primary reason for emphasizing DTR over inter-day temperature variation is that DTR captures daily temperature fluctuation, which is particularly sensitive to short-term, local climatic conditions such as cloud cover, solar radiation, and humidity. These daily variations are more evident in tropical regions such as Thailand, where significant temperature differences between day and night occur, especially during the dry season, when clear skies and intense solar heating lead to large diurnal temperature changes. In contrast, inter-day variation typically represents broader and longer-term trends that provide valuable insights into the overall climate patterns but may not capture the immediate daily fluctuations. As Thailand experiences a high level of temperature variability, and the increasing frequency of extreme weather events [22, 23], understanding the relationship between DTR and mortality is critical for informing public health strategies and mitigating temperature-related health risks. A number of previous studies in Thailand have investigated the relationship between temperature and mortality in specific provinces or regions [24–26]. A more recent nationwide study examined the effects of both low and high temperatures on hospital admission [27]. However, to date, only one study has considered the DTR, and that was limited to hospital admissions in Bangkok [14]. In light of these concerns, the aim of this study was to quantify the burden of mortality attributable to DTR in Thailand over a 15-year period (2007–2021). Using a nationwide case-crossover design, this study investigates the short-term effects of diurnal temperature fluctuations on mortality, focusing on the immediate impact of temperature variability on public health.

## Methodology

**Fig. 1:**
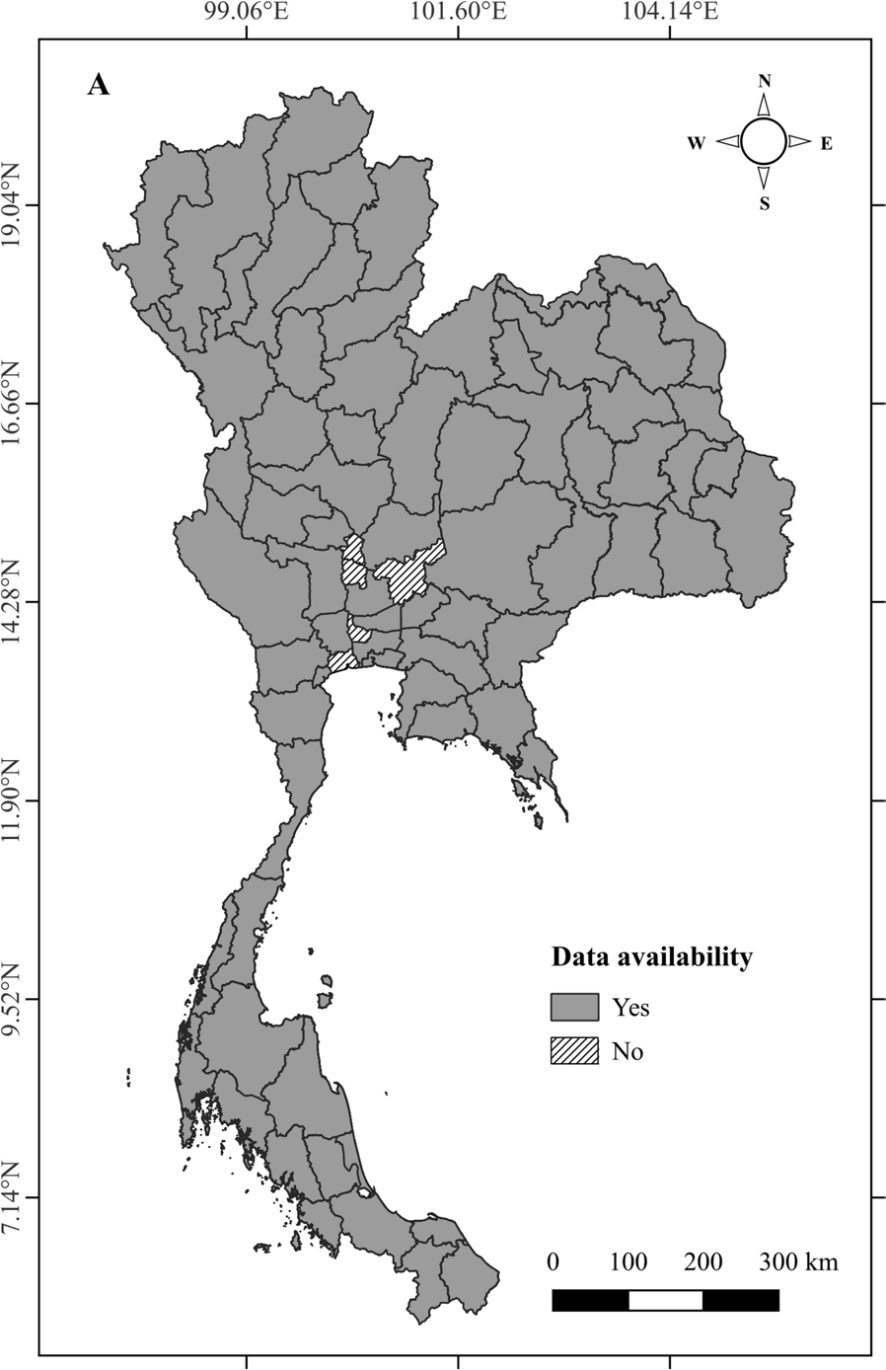
Map of Thailand indicating the included and excluded provinces

This study was conducted across Thailand, utilizing data from 72 out of 77 provinces. The provinces of Ang Thong, Nonthaburi, Samut Sakhon, Saraburi, and Sing Buri were excluded from the analysis due to incomplete meteorological data for the study period (2007–2021) (Fig. 1). The selected provinces represent a variety of geographic, climatic, and socioeconomic characteristics enabling a comprehensive analysis of the mortality burden attributable to DTR. Thailand’s climate varies from tropical in the south to more temperate in the north, providing a broad spectrum of temperature conditions for the analysis of temperature-related mortality patterns. Thailand’s population is distributed unevenly across these provinces, with major urban centers such as Bangkok, contributing significantly to the total population. By focusing on 72 provinces, this study captures a substantial portion of Thailand’s population while accounting for provincial variations in climatic conditions. This comprehensive coverage enhances the generalizability of the findings to a national level, while also identifying specific provincial variations in the burden of mortality attributable to DTR.

### Meteorological data

Meteorological data of this study were obtained from the Thai Meteorological Department (TMD), which is the national authority responsible for collecting and disseminating meteorological data in Thailand. The daily data obtained from the TMD included minimum temperature, maximum temperature, average temperature, and relative humidity. These variables were recorded by permanent weather monitoring stations situated in each province in Thailand over the study period from 2007 to 2021. The DTR is a measure of the daily variation in temperature, calculated as the difference between the daily maximum and minimum temperatures for each day across the entire study period and for each of the 72 provinces [14]. This calculation provides a daily measure of how much temperature fluctuates between the hottest and coldest points of the day.

### Mortality data

Mortality data were obtained from the Strategy and Planning Division under the Office of the Permanent Secretary, Ministry of Public Health, which is responsible for collecting, managing, and disseminating health data in Thailand. The Division maintains an extensive database of the mortality records, which includes anonymized data for individuals who have passed away in Thailand, based on death certificates submitted to the local authorities. Specifically, when an individual passes away, the attending healthcare provider issues a medical certificate that includes details such as the cause of death, date of death, and other demographic information (e.g., age, gender) for the deceased’s relatives, and they need to submit all the necessary information and documents to the registrar at the local authority within 24 h. to issue death certificate. If the death occurs outside of a medical facility, a local authority or district office may verify the cause of death and issue a certificate. Death certificates have been submitted to the Department of Provincial Administration under the Ministry of Interior, which is responsible for maintaining vital records. The information from these certificates is then aggregated at the national level by the Strategy and Planning Division, which compiles data on all reported deaths and ensures accurate registration and categorization according to the International Classification of Diseases, 10th Revision (ICD-10). The coverage of death registration in Thailand is generally high, with an estimated 99% in most areas of the country [28]. Therefore, the registration system in Thailand is considered robust and reliable for national mortality data. In this study, we obtained the data for all-cause mortality, categorized according to ICD-10 codes (A00-R99), and the date of death, as recorded on death certificates.

### Data quality and consistency

Mortality data were obtained from the Strategy and Planning Division under the Office of the Permanent Secretary under Ministry of Public Health, using standardized national reporting systems that remained consistent throughout the study period (2007–2021). According to statistical report conducted by the National Statistical Office (NSO), the completeness of death registration in Thailand was generally high and steadily improving, from 94.8% in 1995–1996 to 98.4% in 2005–2006, reaching 99.3% in 2015–2016 [24]. As our study period began in 2007, when completeness already exceeded 98%, the risk of underreporting bias due to incomplete registration is minimal. Furthermore, meteorological data were sourced from the TMD, which applies consistent measurement protocol and instrumentation throughout the country. Both temperature and mortality time-series were checked for continuity, missing data, and outliers. The mortality dataset was not incomplete, and temperature data displayed expected seasonal and interannual variation without any structural shifts, indicating stable data collection practices (Figs. S1-S4). There were no major health policy changes, disasters, or systemic disruptions during the study period that would plausibly account for the long-term trends observed in mortality. The gradual increase in mortality observed across all provinces is more likely attributable to demographic factors such as population aging, rather than data inconsistency.

### Statistical analyses

Data analysis for this study was conducted in three main sections to estimate the association between DTR and all-cause mortality at both provincial and national levels, as well as to quantify the burden of mortality attributable to DTR: (1) estimating province-specific associations using a time-stratified case-crossover design with a conditional Poisson regression; (2) pooling these estimates at the national level using multivariate meta-regression; and (3) calculating the attributable mortality burden due to DTR.

In the first section, a time-stratified case-crossover design was utilized to estimate the province-specific associations between DTR and mortality. For this design, three-way interaction between year, calendar month, and day of the week was specified to compare DTR levels between case day and its control days that were matched within the same stratum. Specifically, each stratum was defined by matching case day with many control days that shared the same day of the week within the same calendar month and year. This design automatically controls for seasonality and long-term trends, and day of the week that are constant within the same stratum [29]. The province-specific DTR–mortality associations were analyzed using a distributed lag non-linear model (DLNM) combined with a conditional Poisson regression model to account for the overdispersion. The DLNM was used to model the relationship between DTR and mortality across various lags, incorporating the natural cubic splines *(ns)* function for both the DTR and its lag period. The cross-basis matrix between DTR and its lag was constructed using the *ns* function with an optimum degree of freedom (DF), which was selected by minimizing the quasi-Akaike Information Criterion (Q-AIC) [30]. In particular, the DTR was modeled using the *ns* function, with 3 DFs by placing the spline knots at equally spaced intervals based on the province-specific distribution of DTR. The lagged periods of DTR were considered up to 21 days and modeled using the *ns* function, with 4 DFs by the placing spline knots at equally spaced intervals on a logarithmic scale of lags (Table S1). Furthermore, to assess the delayed effects of DTR on mortality, we considered three lag structures, which are lag 0–7 day, lag 0–14 day, and lag 0–21 day, to explore the sensitivity of effect estimates. These lag periods were chosen based on biological plausibility and previous literature indicating that temperature-related health effects may occur both immediately and several days after exposure. Comparison across lag periods enabled us to identify the delayed effects of DTR on mortality, and select the most appropriate lag window for final interpretation. The 21-day lag was ultimately selected as the primary model based on prior literature and biological plausibility [19, 36], and it adequately captured both immediate and delayed effects of DTR on mortality, which is also supported by the lag–response patterns indicated in Fig. S5. This model was additionally adjusted for average temperature and relative humidity, both of which were modeled using the *ns* function with 3 DFs each. We assessed the correlations among independent variables using Pearson correlation. The results showed weak correlations between DTR and mean temperature (r = − 0.07), mean temperature and relative humidity (r = − 0.12), and moderate negative correlation between DTR and relative humidity (r = − 0.64) (Fig. S6), indicating that multicollinearity is not likely to bias the model estimates. Therefore, all three variables were included in the model. A dummy variable for public holidays was also included to control for potential effects of these days on mortality. The formula used to estimate the province-specific associations between DTR and mortality is described below.

1 $${\text{log}}\left[ {{\text{E}}\left( {{\text{Y}}_{{{\text{t}},{\text{i}}}} } \right)} \right] \, = \, \alpha \, + \, \beta \left( {{\text{DTR}}_{{{\text{t}},{\text{l}},{\text{i}}}} } \right) \, + {\text{ ns}}\left( {{\text{Temp}}_{{{\text{t}},{\text{i}}}} ,{ 3}} \right) \, + {\text{ ns}}\left( {{\text{RH}}_{{{\text{t}},{\text{i}}}} ,{ 3}} \right) \, + {\text{ holiday}}_{{\text{t}}} + {\text{ stratum}}\left( {{\text{year}}:{\text{month}}:{\text{day}}} \right)_{{{\text{t}},}}$$

where E(Y_t,i_) is the expected number of deaths at day *t* and province *i*, α is the model intercept, β is the log-relative risk of mortality associated with DTR values, DTR_t,l,i_ indicates diurnal temperature range at day *t* and lag *l* of province *i*, Temp_t,i_ is the mean temperature at day *t* in province *i*, RH_t,i_ is the relative humidity at day *t* in province *i*. The ns() is the natural cubic spline function with predefined degree of freedom (DF), and holiday_t_ is a dummy variable for public holiday at day *t*. stratum(year:month:day)_t_ is a stratum defined by a three-way interaction of year, month, and day of the week at day *t*.

In the second section, the province-specific estimates derived from the first section were then pooled to obtain the national-level estimate of the DTR–mortality association. This was performed by using the multivariate meta-regression, which allowed for the synthesis of individual province-level results into a single national-level estimate. In a meta-regression model, the minimum mortality diurnal temperature range (MM-DTR) was specified as the optimum DTR, corresponding to DTR level that was associated with the lowest risk of mortality. While the framework allows for the inclusion of meta-predictors, we did not include any specific meta-predictors in our model due to limited availability of province-level covariate data. The multivariate extension of *I*^*2*^ statistics and the Cochran *Q*-test were used to assess the residual heterogeneity between the province-specific estimates, ensuring that any variation between provinces was appropriately accounted for [31]. In particular, the null hypothesis of the Cochran *Q*-test is that all province-specific estimates share a common effect size, meaning there is no heterogeneity beyond that expected by random error. Furthermore, the best linear unbiased prediction (BLUP) approach was used to obtain more stable province-specific estimates. This method improves the precision of province-specific estimates by allowing information to be shared across provinces, so that estimates from provinces with smaller number of deaths or narrower DTR ranges can be improved by borrowing information from provinces with larger sample sizes [32]. The BLUP results were then used to define the DTR–mortality association for each province, which was further applied to estimate the province-specific mortality burden attributable to DTR.

In the third section, the mortality burden attributable to the variations in DTR was calculated following the attributable risk measurement within the DLNM framework by looking the estimated risk from the current day to past exposures, according to the method proposed by Gasparrini and Leone, 2014 [33]. This approach estimates the attributable risk by summing the contributions of past exposures, weighted by the corresponding lag–response associations, to quantify the cumulative effects of DTR on mortality. The province-specific MM-DTR was defined from BLUP estimate of each province, and the number and fraction of mortality attributable DTR both above and below the MM-DTR was quantified using the following equation:

2 $${\text{AF}}_{{\text{x}}} = { 1 }{-}{\text{ exp}}\left( {{-} \, \beta_{{\text{x}}} } \right),$$

3 $${\text{AN}}_{{\text{x}}} = {\text{ AF}}_{{\text{x}}} *{\text{ N}}_{{\text{x}}} ,$$

where β_x_ is the estimated risk associated with DTR level *x* relative to the MM-DTR. *x* is the individual DTR level at a given day, while N_x_ indicates total number of deaths for DTR level *x*. AF_x_ and AN_x_ are the attributable fraction and attributable number for DTR level x, respectively, which incorporates the effects of DTR on mortality over various lag periods [24, 33]. For example, the AF for a given DTR level, such as 10 °C (AF_10_), can be computed by comparing it to the MM-DTR at lags ranging from 0 to 14 days. The formula used is 1 − exp[− (β_0_ + β_1_ + β_2_ + … + β_14_)], where β_lag_ represents the mortality risk associated with DTR at each lag (lag = 0, 1, 2,…, 14 days). The AN_10_ was calculated by multiplying AF_10_ with the average daily number of deaths over the preceding 15 days (lags 0–14) [24]. This method was performed across the DTR distribution for each province to obtain province-specific AF and AN. A Monte Carlo simulation was conducted 1000 times to estimate the 95% empirical confidence interval (95% eCI), assuming a normal distribution for the BLUP [33]. To estimate the national-level AF, the total AN obtained from 72 provinces were summed and divided by the total number of deaths during the study period.

Several sensitivity analyses were conducted to assess the reliability of the effect estimates. This involved adding autocorrelation to the conditional Poisson model by incorporating the model residual at a 1-day lag [34]. Additionally, alternative DF for the natural cubic spline of the DTR variable and its lag in the cross-basis matrix of the DLNM function were specified. All statistical analyses were carried out using the R (version 4.3.3), with the *dlnm*, *gnm*, and *mvmeta* packages.

## Results

Summary statistics for daily DTR, average temperature, relative humidity, and all-cause mortality from 72 provinces of Thailand during 2007–2021 are shown in Table 1. Daily average (± standard deviation) of DTR, average temperature, and relative humidity was 9.8 (± 3.1) °C, 27.5 (± 2.6) °C, and 76.2 (± 9.3) %, respectively. A total of 5,574,850 cases of all-cause mortality were reported over 72 provinces during the study period, with the average of 15 (± 14) cases per day. The province-specific summary statistics for meteorological variable and all-cause mortality are indicated in Table S2.

**Table 1:** Summary statistics for DTR, mean temperature, relative humidity, and number of deaths during 2007–2021 in 72 provinces of Thailand

Variables	Mean	S.D	Min	P25	P50	P75	Max	Total
DTR (°C)	9.8	3.1	0.1	7.8	9.5	11.5	26.1	–
Mean temperature (°C)	27.5	2.6	7.0	26.5	27.8	29.1	36.8	–
Relative humidity (%)	76.2	9.3	31.0	70.5	77.3	83.0	100.0	–
Number of deaths	15	14	1	7	12	19	171	5,574,850

*S.D.* standard deviation, *Min* minimum, *Max* maximum, *P25* 25th percentile, *P50* median (50th percentile), *P75* 75th percentile

**Fig. 2:**
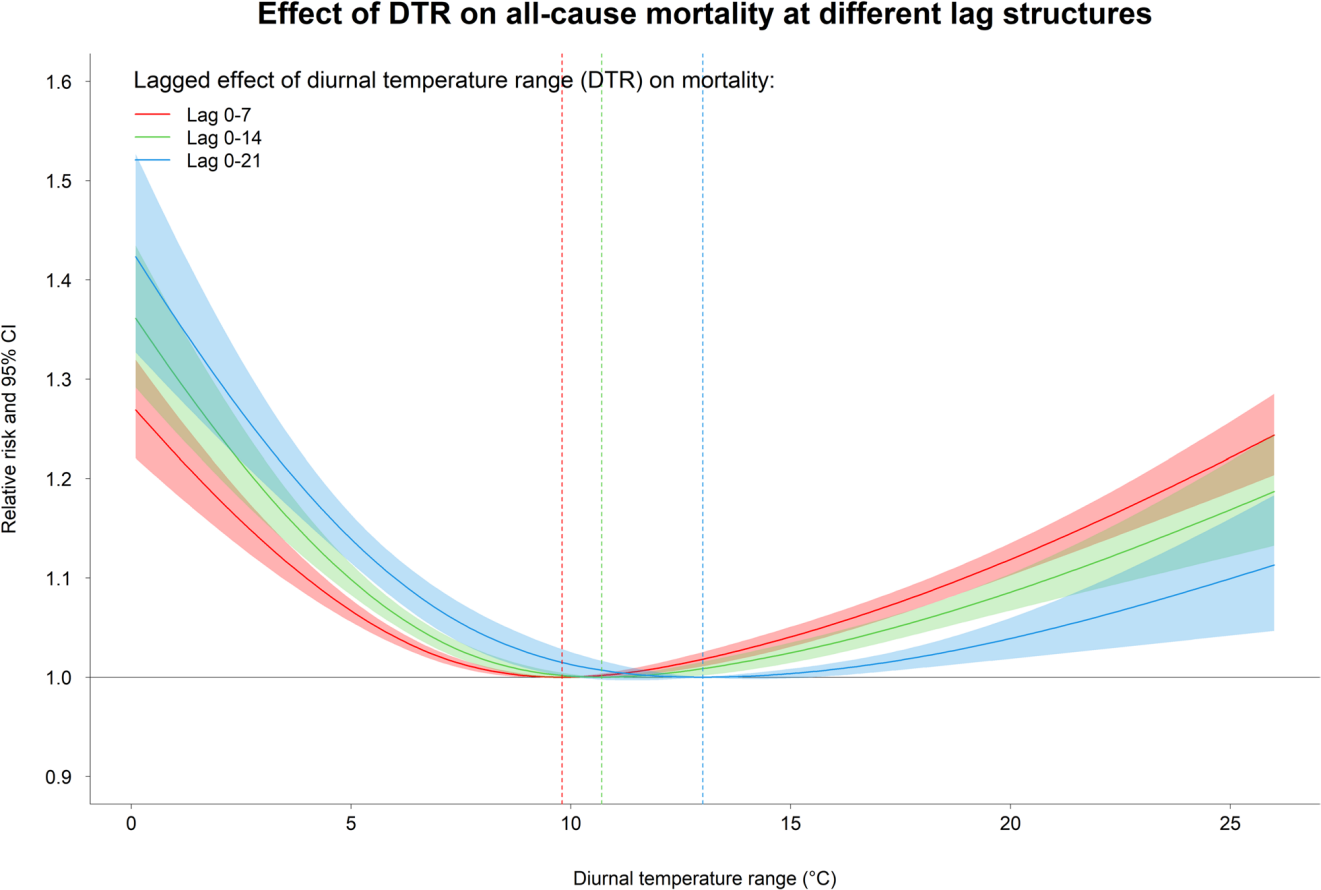
The pooled cumulative relative risk of diurnal temperature range (DTR) on all-cause mortality in Thailand at different lag structures. The vertical dotted lines highlighted in red, green, and blue indicate the MM-DTR for lag 0–7, 0–14, and 0–21, respectively

We observed non-linear, with U-shaped pattern, of the association between DTR and all-cause mortality in Thailand, and the estimates varied by lagged period (Fig. 2). We also found significant heterogeneity of the effect estimates among provinces, as indicated by an *I*^*2*^ statistics of 41.5% (*p*-value of Cochran *Q*-test < 0.001). The relative risk (RR) of mortality associated with specific DTRs at different lag periods is reported in Table 2. For cumulative lag 0–7 day, the national-level RRs of mortality associated with 1st (3.3 °C), 10th (6.4 °C), 90th (13.3 °C), and 99th (16.1 °C) DTR percentiles relative to the MM-DTR (9.8 °C) were, respectively, 1.1713 (95% confidence interval (CI) 1.1433–1.2000), 1.0512 (95% CI 1.0409–1.0616), 1.0107 (95% CI 1.0032–1.0183), and 1.0356 (95% CI 1.0245–1.0468). For cumulative lag 0–14 day, the national-level RRs of mortality associated with 1st, 10th, 90th, and 99th DTR percentiles relative to the MM-DTR (10.7 °C) were 1.1231 (95% CI 1.1027–1.1439), 1.0313 (95% CI 1.0249–1.0378), 1.0212 (95% CI 1.0135–1.0289), and 1.0552 (95% CI 1.0441–1.0664), respectively. For cumulative lag 0–21 day, the national-level RRs of mortality associated with the 1st, 10th, 90th, and 99th DTR percentiles relative to the MM-DTR (13.0 °C) were 1.2196 (95% CI 1.1815–1.2589), 1.0850 (95% CI 1.0642–1.1063), 1.0001 (95% CI 0.9991–1.0010), and 1.0085 (95% CI 1.0019–1.0152), respectively. The lag–response curve illustrating the pooled RR of mortality by lag at 1st, 10th, 90th and 99th percentiles of DTR is shown in Fig. S5. The result indicates that lower DTR levels (a small difference between daily maximum and minimum temperatures), represented by the 1st and 10th percentiles, were associated with a delayed but prolonged increase in mortality risk, peaking a few days after exposure. In contrast, higher DTR levels, represented by the 90th and 99th percentiles, were associated with an immediate increase in mortality risk, with the peak occurring on the same day or within a few days after exposure. The province-specific BLUP estimates of all-cause mortality associated with different DTR percentiles and lag periods can be found in Table S3.

**Table 2:** The relative risk (RR) of mortality associated with specific DTRs relative to the minimum mortality DTR (mm-DTR) at different lag structures

Lag (days)	MM-DTR (°C)	Percentiles	DTR (°C)	RR and 95% CI		
				RR	Lower	Upper
0–7	9.8	1st	3.3	1.1713	1.1433	1.2000
0–7	9.8	10th	6.4	1.0512	1.0409	1.0616
0–7	9.8	90th	13.3	1.0107	1.0032	1.0183
0–7	9.8	99th	16.1	1.0356	1.0245	1.0468
0–14	10.7	1st	3.3	1.1231	1.1027	1.1439
0–14	10.7	10th	6.4	1.0313	1.0249	1.0378
0–14	10.7	90th	13.3	1.0212	1.0135	1.0289
0–14	10.7	99th	16.1	1.0552	1.0441	1.0664
0–21	13.0	1st	3.3	1.2196	1.1815	1.2589
0–21	13.0	10th	6.4	1.0850	1.0642	1.1063
0–21	13.0	90th	13.3	1.0001	0.9991	1.0010
0–21	13.0	99th	16.1	1.0085	1.0019	1.0152

The mortality fraction and number attributable to non-MM-DTR at different lag periods are reported in Table 3. In particular, the fraction of all-cause mortality attributable to non-MM-DTR was 1.88% (95% empirical confidence interval (eCI) 0.69–3.03) at cumulative lag 0–7 day. For cumulative lag 0–14 day, the mortality fraction attributable to non-MM-DTR was 2.39% (95% eCI 0.75–3.99), and that at cumulative lag 0–21 day was 4.67 (95% eCI: − 1.14–9.87). The province-specific all-cause mortality fractions attributable to non-MM-DTR at different lag structures are shown in Table S4.

**Table 3:** The mortality fraction (%) and number attributable to non-minimum mortality DTR (non-MM-DTR) at different lag periods

Lag	Total deaths	AF (%) and 95% eCI			AN (cases) and 95% eCI		
		**AF**	**Lower**	**Upper**	**AN**	**Lower**	**Upper**
0–7	5,574,850	1.88	0.69	3.03	104,807	38,466	168,918
0–14	5,574,850	2.39	0.75	3.99	133,239	41,811	222,437
0–21	5,574,850	4.67	-1.14	9.87	260,345	-63,553	550,238

*AF* attributable fraction, *AN* attributable number, *eCI* empirical confidence interval

The results from the sensitivity analyses indicated that the national-level effect estimates of DTR on mortality were generally consistent. In particular, after accounting for autocorrelation in the conditional Poisson model by incorporating a 1-day lag of the model residuals, as well as adjusting for different DFs of the *ns* function for the DTR variable and its lag in the cross-basis matrix of the DLNM function, the DTR–mortality association curve remained similar (Figs. S7–S12). These adjustments did not substantially alter the overall findings, suggesting the robustness of the effect estimates.

## Discussion

This nationwide case-crossover analysis examined the burden of all-cause mortality attributable to DTR in Thailand over a 15-year period (2007–2021). Our findings indicate a significant, non-linear, U-shaped association between DTR and all-cause mortality. This suggests that both extreme low and high DTR values are associated with increased risks of mortality, especially when DTR deviates significantly from the MM-DTR. These results contribute to the growing body of evidence linking temperature variability to health outcomes, and they highlight the importance of considering DTR, in addition to average temperature, when evaluating the impacts of climate change on public health.

Our analysis revealed that the effect of DTR on mortality in Thailand was not uniform across all levels of DTR. The relationship followed a U-shaped pattern, where both lower and higher levels of DTR were associated with increased risk of mortality. This pattern is consistent with many previous studies from other regions [19, 35, 36], which suggest that both cold and hot temperature conditions, particularly when combined with large temperature variability between day and night, may exert adverse effects on human health [12, 37]. Specifically, the RR of mortality for specific DTR percentiles (relative to the MM-DTR) was higher during certain cumulative lag periods, with cumulative lag 0–21 days showing the most pronounced estimated effects. These findings suggest that the impact of DTR on mortality may be delayed, possibly due to the cumulative physiological stress caused by temperature fluctuations over an extended period [38].

The fraction of mortality attributable to non-MM-DTR at different lag periods was also assessed, with notable findings. Specifically, at a cumulative lag of 0–7 day, 1.88% of all-cause mortality was attributed to non-MM-DTR. This fraction increased to 2.39% at a cumulative lag of 0–14 day and further to 4.67% at a cumulative lag of 0–21 day. While the latter estimate showed a wide confidence interval, indicating some uncertainty, the general trend suggests that the mortality burden associated with DTR increases with longer cumulative lag periods [38]. This finding supports the hypothesis that prolonged exposure to abnormal DTR levels may lead to higher mortality risks, particularly in vulnerable populations [39, 40]. It is important to note that mortality fraction attributable to non-MM-DTR is relatively low compared to other well-established risk factors for mortality, such as non-communicable diseases [41].

The estimated risk of both lower and higher levels of DTR on mortality can be explained by different physiological and environmental mechanisms. In particular, a low DTR, often observed in climates with consistent temperatures or overcast conditions, might indicate reduced weather variability, which could lead to prolonged exposure to unfavorable conditions, such as persistent cold or heat, increasing the risk of both cardiovascular and respiratory diseases [42, 43]. On the other hand, a high DTR, which refers to significant temperature fluctuations between day and night, can stress the body due to its inability to adapt quickly to rapid changes in temperature, leading to greater health risk, particularly for vulnerable populations such as the elderly or those with pre-existing health conditions [12, 14, 15]. Both extremes of DTR can disrupt the thermoregulation processes of human body, potentially contributing to increased mortality rates from heat-related or cold-related issues, cardiovascular events, and respiratory disorders [44, 45].

We also found substantial heterogeneity in the mortality effects of DTR across provinces, as indicated by the *I*^*2*^ statistics and Cochran *Q*-test, suggesting that the relationship between DTR and mortality may vary geographically, possibly due to regional differences in socioeconomic status, health infrastructure, air pollution exposure, adaptive capacity (e.g., air conditioning prevalence or housing condition), or population vulnerabilities [46–49]. These differences highlight the importance of considering the local context when assessing climate-related health risks. For instance, provinces with higher levels of air pollution, or populations more susceptible to extreme weather may experience different mortality risks associated with DTR, where further studies could explore these regional disparities to guide targeted interventions.

Our sensitivity analyses revealed that even when we incorporated autocorrelation into the model and adjusted for different DFs in the ns function for the DTR variable and lag, the DTR–mortality association curve remained stable. This suggests that our results were not overly sensitive to model assumptions or the choice of statistical methods specified.

A notable strength of this manuscript is its focus on Thailand, a tropical country that experiences unique temperature patterns and environmental conditions. The nationwide analysis of DTR and its association with all-cause mortality over 15 years offers important insights into the health impacts of temperature fluctuations in tropical regions, which are often underrepresented in global climate-health studies. The study utilizes a robust time-stratified case-crossover design to capture the relationship between DTR and mortality across different parts of Thailand, while accounting for varying lag periods and regional heterogeneity across 72 provinces. The non-linear, U-shaped association between DTR and mortality, along with the significant variability in the effect estimates across provinces, provides valuable insights into the complex relationship between temperature fluctuations and public health. While some previous studies have explored the effects of DTR on cause-specific mortality and have identified the vulnerable subpopulations based on age and sex, such studies have often been limited to specific regions [14, 40, 50, 51]. In contrast, our study offers comprehensive and consistent assessment of DTR-related mortality burden across a diverse range of geographic and socioeconomic contexts within Thailand. This large-scale approach enhances the generalizability of the findings and offers critical baseline evidence that can support national-level public health planning and adaptation strategies. By focusing on all-cause mortality, our study also provides a holistic estimate of DTR’s overall health impact, capturing a broader range of potential effects, including those not attributed to a single specific cause. This comprehensive perspective is particularly valuable for informing public health interventions and resource allocation in the context of climate variability and provides a comprehensive initial assessment of DTR impacts that can serve as a foundation for future analyses. While our study provides important insights into health impacts of temperature fluctuation in Thailand, some limitations should be acknowledged. First, the study relied on aggregated data from provinces, which may bias the effect estimate towards the null. Second, we did not adjust for the effects of air pollution, which could confound the DTR–mortality association. However, a previous study has revealed that air pollution did not significantly alter the DTR–mortality association [14]. Third, a key limitation of this study is the use of all-cause mortality as the outcome variable. Due to data access restrictions, the individual ICD-10 codes were not available, which limited an analysis of cause-specific mortality. In addition, detailed demographic information such as age and sex were not accessible, which precluded stratified analyses to examine potential differences in vulnerability across population subgroups. As a result, the findings may not reflect disease-specific or demographic-specific vulnerabilities to DTR, limiting the ability to inform targeted public health interventions. Future research with access to more detailed mortality records should explore both cause-specific and subgroup-specific associations to understand the mechanisms and disparities underlying DTR-related mortality. Moreover, one limitation of this study is the presence of heterogeneity in the pooled effect estimates that was not addressed through the inclusion of meta-predictors in the meta-analysis. While we applied a consistent modeling framework across provinces, regional differences in contextual and environmental factors likely contributed to variation in effect estimates. In the Thai context, these may include local climate variables, socioeconomic status, access to healthcare services, and adaptive capacity such as housing conditions. Future studies should consider adjusting for such meta-predictors in meta-regression models to more accurately identify and explain sources of heterogeneity in DTR-related mortality association.

## Conclusions

Our study highlights the significant burden of mortality attributable to DTR in Thailand, with a clear, non-linear, U-shaped association observed across different lag periods. This finding highlights the significance of DTR as a climatic variable with potential health implications in Thailand. Although no early warning system related to DTR currently exists, the Thai Meteorological Department provides daily forecasts of the minimum and maximum temperature, which can serve as a foundation for DTR monitoring. The localized interventions, such as heat-health action plans, urban greening projects, and community-level health advisories, are already in place and can be strengthened further. Incorporating DTR-specific alerts into existing meteorological and public health frameworks could enhance early warning capacities and improve public resilience to temperature-related health risks in Thailand. This is particularly relevant in the context of climate change, where increasing temperature extremes could amplify the health risks associated with DTR. Policy makers should consider DTR as a potential risk factor in public health planning and climate adaptation strategies.

## Supplementary Information

Additional file 1.

## Data Availability

The data that support findings of this study are available in supporting file.
