# Supplementary material for: Burden of mortality attributable to diurnal temperature range in Thailand: a nationwide case-crossover analysis from 2007 to 2021
Source: Trop Med Health. 2025 Jun 3;53:78. doi: 10.1186/s41182-025-00761-1 (PMC12131491; doi:10.1186/s41182-025-00761-1)
Supplement: Supplementary file 1 — Additional file 1. [file 41182_2025_761_MOESM1_ESM.docx]

**The burden of non-accidental mortality attributable to diurnal temperature range in Thailand: A nationwide case-crossover analysis from 2007 to 2021**

Chittamon Sritong-aon^1^, Arthit Phosri^1,2,*^, Tanasri Sihabut^1,2^, Tawach Prechthai ^1,2^

^1^Department of Environmental Health Sciences, Faculty of Public Health, Mahidol University, Bangkok, Thailand

^2^Center of Excellence on Environmental Health and Toxicology (EHT), Office of the Permanent Secretary (OPS), Ministry of Higher Education, Science, Research and Innovation (MHESI), Bangkok, Thailand

*Address correspondence to Arthit Phosri, Department of Environmental Health Sciences, 4th Floor, 2nd Building, Faculty of Public Health, Mahidol University, Rajvithi Road, Bangkok 10400, Thailand. Telephone: +66 2354 8525.

E-mail: [arthit.pho@mahidol.ac.th](mailto:arthit.pho@mahidol.ac.th)

**Lists of Tables**

**Table S1** The quasi-Akaike Information Criterion (Q-AIC) obtained from a model that used different degrees of freedom for DTR variable and its lag in the cross-basis matrix

**Table S2** Summary statistics of DTR, mean temperature, relative humidity, and number of deaths during 2007–2021 in 72 provinces of Thailand

**Table S3** The province-specific BLUP estimates of all-cause mortality associated with different DTR percentiles and lag periods relative to the MM-DTR

**Table S4** The province-specific mortality fractions attributable to non-minimum mortality DTR (non-MM-DTR) at different lag structures

**Lists of Figures**

**Fig. S1** Daily number of deaths over time for each province included in this study

**Fig. S2** Daily mean temperature over time for each province included in this study

**Fig. S3** Daily minimum temperature over time for each province included in this study

**Fig. S4** Daily maximum temperature over time for each province included in this study

**Fig. S5** The pooled relative risk (RR) of all-cause mortality by lag at 1st, 10th, 90th and 99th percentiles of diurnal temperature range (DTR)

**Fig. S6** The Pearson correlation coefficients among independent variables

**Fig. S7** The curve of DTR-mortality association at the national-level obtained from the model with and without adjustment for autocorrelation at cumulative lag 0-7 day.

**Fig. S8** The curve of DTR-mortality association at the national-level obtained from the model with and without adjustment for autocorrelation at cumulative lag 0-14 day.

**Fig. S9** The curve of DTR-mortality association at the national-level obtained from the model with and without adjustment for autocorrelation at cumulative lag 0-21 day.

**Fig. S10** The curve of DTR-mortality association at the national-level, adjusted for different DFs of the *ns* function for the DTR variable and its lag in the cross-basis matrix at the cumulative lag 0-7 day

**Fig. S11** The curve of DTR-mortality association at the national-level, adjusted for different DFs of the *ns* function for the DTR variable and its lag in the cross-basis matrix at the cumulative lag 0-14 day

**Fig. S12** The curve of DTR-mortality association at the national-level, adjusted for different DFs of the *ns* function for the DTR variable and its lag in the cross-basis matrix at the cumulative lag 0-21 day

**Table S1** The quasi-Akaike Information Criterion (Q-AIC) obtained from a model that used different degrees of freedom for DTR variable and its lag in the cross-basis matrix

| **Provinces** | **Degrees of freedom (DTR, Lag)** | | | | | | |
| --- | --- | --- | --- | --- | --- | --- | --- |
|  | **(3,3)** | **(4,3)** | **(5,3)** | **(6,3)** | **(3,6)** | **(3,5)** | **(3,4)** |
| Amnat Charoen | 3222.16 | 3227.53 | 3233.00 | 3233.22 | 3239.07 | 3232.01 | 3227.42 |
| Bangkok | 40502.09 | 40507.72 | 40511.64 | 40513.02 | 40498.88 | 40492.63 | 40497.69 |
| Bueng Kan | 2755.08 | 2758.74 | 2760.28 | 2763.57 | 2769.12 | 2763.72 | 2758.53 |
| Buri Ram | 31807.23 | 31810.43 | 31814.71 | 31814.05 | 31754.46 | 31751.53 | 31754.49 |
| Chachoengsao | 27219.69 | 27222.29 | 27216.11 | 27222.52 | 27218.64 | 27217.97 | 27213.04 |
| Chai Nat | 24611.50 | 24617.61 | 24623.98 | 24630.31 | 24621.59 | 24618.46 | 24613.21 |
| Chaiyaphum | 27559.29 | 27563.17 | 27565.79 | 27568.88 | 27539.35 | 27534.72 | 27530.17 |
| Chanthaburi | 25350.79 | 25355.19 | 25358.86 | 25364.30 | 25353.98 | 25349.58 | 25344.53 |
| Chiang Mai | 34238.30 | 34238.24 | 34235.30 | 34242.39 | 34236.55 | 34231.90 | 34232.24 |
| Chiang Rai | 31112.10 | 31116.64 | 31106.12 | 31110.49 | 31087.02 | 31083.59 | 31083.56 |
| Chon Buri | 31902.91 | 31888.17 | 31895.55 | 31898.53 | 31915.53 | 31909.22 | 31905.68 |
| Chumphon | 24774.89 | 24771.07 | 24765.54 | 24769.08 | 24771.41 | 24769.16 | 24767.62 |
| Kalasin | 28262.70 | 28266.22 | 28270.39 | 28275.43 | 28240.56 | 28241.13 | 28249.17 |
| Kamphaeng Phet | 27663.92 | 27664.04 | 27667.97 | 27675.53 | 27653.41 | 27647.37 | 27646.04 |
| Kanchanaburi | 27638.86 | 27638.90 | 27642.28 | 27647.85 | 27642.14 | 27639.77 | 27637.16 |
| Khon Kaen | 33909.62 | 33909.91 | 33912.14 | 33919.13 | 33864.41 | 33863.87 | 33869.84 |
| Krabi | 22235.69 | 22233.19 | 22238.87 | 22241.94 | 22242.73 | 22238.48 | 22239.14 |
| Lampang | 30252.27 | 30254.74 | 30251.05 | 30255.29 | 30244.40 | 30241.24 | 30239.02 |
| Lamphun | 25775.36 | 25778.70 | 25783.99 | 25787.72 | 25772.52 | 25767.84 | 25766.11 |
| Loei | 26765.95 | 26769.86 | 26773.42 | 26775.80 | 26744.30 | 26740.06 | 26740.22 |
| Lop Buri | 29147.73 | 29149.16 | 29155.51 | 29156.96 | 29135.82 | 29132.29 | 29140.23 |
| Mae Hong Son | 18226.12 | 18229.82 | 18234.51 | 18238.99 | 18232.13 | 18229.82 | 18225.96 |
| Maha Sarakham | 27457.25 | 27460.19 | 27464.07 | 27468.91 | 27445.83 | 27439.34 | 27446.20 |
| Mukdahan | 21169.20 | 21169.69 | 21171.04 | 21176.92 | 21168.89 | 21166.02 | 21161.73 |
| Nakhon Nayok | 3998.94 | 4001.23 | 3997.15 | 3998.37 | 4006.19 | 4009.59 | 4003.49 |
| Nakhon Pathom | 27595.39 | 27598.58 | 27601.03 | 27606.41 | 27583.24 | 27579.68 | 27577.97 |
| Nakhon Phanom | 27390.64 | 27395.90 | 27400.11 | 27402.72 | 27364.04 | 27360.37 | 27358.10 |
| Nakhon Ratchasima | 36312.53 | 36313.29 | 36320.81 | 36326.34 | 36270.29 | 36264.42 | 36263.53 |
| Nakhon Sawan | 30850.71 | 30853.40 | 30856.23 | 30851.74 | 30813.55 | 30813.01 | 30817.46 |
| Nakhon Si Thammarat | 31177.54 | 31178.46 | 31182.08 | 31185.61 | 31184.48 | 31184.22 | 31182.91 |
| Nan | 25434.68 | 25423.11 | 25429.77 | 25433.04 | 25442.29 | 25437.48 | 25434.47 |
| Narathiwat | 21102.63 | 21107.06 | 21110.03 | 21112.47 | 21114.50 | 21108.59 | 21106.29 |
| Nong Bua Lam Phu | 12353.59 | 12349.59 | 12353.99 | 12358.68 | 12346.88 | 12341.27 | 12342.91 |
| Nong Khai | 24169.61 | 24172.22 | 24177.00 | 24182.93 | 24168.68 | 24165.76 | 24163.37 |
| Pathum Thani | 28814.95 | 28820.09 | 28825.31 | 28829.19 | 28797.52 | 28804.88 | 28810.98 |
| Pattani | 25649.62 | 25653.59 | 25657.24 | 25662.86 | 25659.64 | 25655.56 | 25652.34 |
| Phangnga | 19266.42 | 19268.22 | 19268.00 | 19269.29 | 19271.38 | 19268.40 | 19265.32 |
| Phatthalung | 24486.69 | 24492.15 | 24493.84 | 24496.03 | 24499.64 | 24493.44 | 24492.13 |
| Phayao | 24327.93 | 24330.87 | 24332.78 | 24337.34 | 24337.88 | 24333.78 | 24331.89 |
| Phetchabun | 29985.53 | 29990.41 | 29992.57 | 29992.05 | 29963.15 | 29963.78 | 29961.34 |
| Phetchaburi | 24964.89 | 24966.23 | 24971.52 | 24975.90 | 24958.88 | 24967.96 | 24962.34 |
| Phichit | 25695.07 | 25690.85 | 25697.98 | 25692.43 | 25694.20 | 25697.70 | 25696.56 |
| Phitsanulok | 27701.85 | 27706.89 | 27712.10 | 27718.37 | 27703.33 | 27700.54 | 27695.30 |
| Phra Nakhon Si Ayutthaya | 28284.04 | 28288.66 | 28289.74 | 28289.51 | 28274.89 | 28272.58 | 28270.94 |
| Phrae | 23913.10 | 23919.22 | 23918.38 | 23922.73 | 23913.91 | 23908.14 | 23903.65 |
| Phuket | 22050.82 | 22052.00 | 22051.18 | 22053.85 | 22051.64 | 22055.30 | 22054.12 |
| Prachin Buri | 25185.05 | 25190.15 | 25188.29 | 25191.13 | 25182.57 | 25177.40 | 25174.56 |
| Prachuab Khiri Khan | 24499.93 | 24504.00 | 24507.59 | 24512.09 | 24501.81 | 24494.94 | 24494.96 |
| Ranong | 14797.24 | 14800.51 | 14798.53 | 14803.58 | 14802.85 | 14800.06 | 14797.90 |
| Ratchaburi | 26962.25 | 26953.46 | 26962.92 | 26959.70 | 26965.06 | 26958.22 | 26960.42 |
| Rayong | 26909.56 | 26908.87 | 26914.23 | 26918.92 | 26911.94 | 26909.28 | 26906.06 |
| Roi Et | 31475.96 | 31476.78 | 31476.16 | 31481.26 | 31446.25 | 31444.06 | 31450.05 |
| Sa Kaeo | 25046.10 | 25050.85 | 25050.69 | 25055.64 | 25053.74 | 25048.49 | 25044.07 |
| Sakon Nakhon | 29884.90 | 29886.17 | 29884.66 | 29889.05 | 29854.40 | 29854.76 | 29868.61 |
| Samut Prakan | 30136.92 | 30135.26 | 30134.70 | 30133.61 | 30152.52 | 30146.31 | 30140.64 |
| Samut Songkhram | 3936.42 | 3941.07 | 3943.44 | 3949.47 | 3951.19 | 3945.06 | 3942.60 |
| Satun | 18975.02 | 18978.25 | 18982.49 | 18986.31 | 18982.99 | 18979.00 | 18975.04 |
| Si Sa Ket | 29426.49 | 29420.15 | 29422.96 | 29427.37 | 29439.17 | 29432.95 | 29429.58 |
| Songkhla | 31449.24 | 31420.42 | 31416.64 | 31422.26 | 31451.12 | 31445.50 | 31444.22 |
| Sukhothai | 27044.28 | 27047.43 | 27051.58 | 27053.09 | 27045.76 | 27040.11 | 27034.99 |
| Suphan Buri | 29584.47 | 29589.74 | 29590.31 | 29593.80 | 29574.54 | 29573.64 | 29568.56 |
| Surat Thani | 28613.10 | 28615.29 | 28621.46 | 28624.50 | 28614.61 | 28607.87 | 28608.77 |
| Surin | 31805.78 | 31808.85 | 31806.23 | 31813.79 | 31793.95 | 31793.34 | 31791.03 |
| Tak | 24918.07 | 24919.70 | 24921.01 | 24924.07 | 24923.44 | 24925.54 | 24920.44 |
| Trang | 24543.15 | 24543.71 | 24542.99 | 24544.67 | 24547.88 | 24547.87 | 24546.67 |
| Trat | 17242.65 | 17247.64 | 17250.76 | 17251.94 | 17252.62 | 17249.05 | 17247.43 |
| Ubon Ratchathani | 32947.66 | 32952.43 | 32952.63 | 32959.68 | 32923.35 | 32921.34 | 32920.05 |
| Udon Thani | 30987.53 | 30993.16 | 30991.22 | 30991.03 | 30962.41 | 30960.11 | 30960.34 |
| Uthai Thani | 4306.67 | 4311.35 | 4316.65 | 4323.46 | 4311.43 | 4305.26 | 4302.82 |
| Uttaradit | 25687.40 | 25692.40 | 25697.14 | 25695.93 | 25695.08 | 25689.73 | 25687.65 |
| Yala | 22506.16 | 22512.00 | 22517.62 | 22517.71 | 22513.00 | 22511.02 | 22510.49 |
| Yasothon | 3344.21 | 3347.44 | 3342.51 | 3342.68 | 3347.55 | 3343.46 | 3348.16 |
| **Average** | 24879.22 | 24880.84 | 24883.00 | 24886.32 | 24875.20 | 24872.12 | **24871.01** |

**Table S2** Summary statistics of DTR, mean temperature, relative humidity, and number of deaths during 2007–2021 in 72 provinces of Thailand

| **Provinces** | **Parameters** | **Mean** | **S.D.** | **Min** | **P25** | **P50** | **P75** | **Max** | **Total** |
| --- | --- | --- | --- | --- | --- | --- | --- | --- | --- |
| Amnat Charoen | DTR (^o^C) | 9.6 | 3.0 | 0.4 | 7.5 | 9.4 | 11.7 | 18.1 | - |
| Amnat Charoen | Mean temperature (^o^C) | 27.1 | 3.0 | 16.2 | 25.2 | 27.7 | 29.3 | 34.3 | - |
| Amnat Charoen | Relative Humidity (%) | 75.5 | 10.6 | 52.0 | 67.0 | 75.0 | 85.0 | 100.0 | - |
| Amnat Charoen | All deaths | 7 | 3 | 1 | 5 | 6 | 8 | 18 | 4787 |
| Bangkok | DTR (^o^C) | 7.8 | 1.6 | 1.6 | 6.9 | 7.8 | 8.8 | 14.7 | - |
| Bangkok | Mean temperature (^o^C) | 29.4 | 1.8 | 18.1 | 28.5 | 29.5 | 30.5 | 34.7 | - |
| Bangkok | Relative Humidity (%) | 71.3 | 8.2 | 45.0 | 66.3 | 71.8 | 76.8 | 95.8 | - |
| Bangkok | All deaths | 104 | 15 | 28 | 94 | 104 | 114 | 171 | 572161 |
| Bueng Kan | DTR (^o^C) | 10.1 | 3.6 | 0.2 | 7.8 | 9.8 | 12.5 | 19.8 | - |
| Bueng Kan | Mean temperature (^o^C) | 27.2 | 3.1 | 15.9 | 25.3 | 27.8 | 29.4 | 34.2 | - |
| Bueng Kan | Relative Humidity (%) | 78.2 | 9.8 | 48.0 | 72.0 | 78.0 | 85.0 | 99.0 | - |
| Bueng Kan | All deaths | 7 | 3 | 1 | 5 | 7 | 9 | 17 | 5210 |
| Buri Ram | DTR (^o^C) | 10.8 | 2.8 | 1.1 | 8.9 | 10.6 | 12.8 | 20.8 | - |
| Buri Ram | Mean temperature (^o^C) | 27.2 | 2.7 | 15.6 | 25.9 | 27.6 | 29.0 | 34.5 | - |
| Buri Ram | Relative Humidity (%) | 75.4 | 9.5 | 40.5 | 69.0 | 76.5 | 82.5 | 98.5 | - |
| Buri Ram | All deaths | 23 | 6 | 1 | 19 | 23 | 27 | 53 | 126666 |
| Chachoengsao | DTR (^o^C) | 10.4 | 2.7 | 1.1 | 8.5 | 10.1 | 12.2 | 21.1 | - |
| Chachoengsao | Mean temperature (^o^C) | 27.4 | 1.9 | 18.4 | 26.4 | 27.5 | 28.6 | 33.6 | - |
| Chachoengsao | Relative Humidity (%) | 79.3 | 8.1 | 46.0 | 74.0 | 80.0 | 85.0 | 98.0 | - |
| Chachoengsao | All deaths | 12 | 4 | 1 | 9 | 12 | 14 | 28 | 65590 |
| Chai Nat | DTR (^o^C) | 9.8 | 2.4 | 1.2 | 8.2 | 9.8 | 11.4 | 18.1 | - |
| Chai Nat | Mean temperature (^o^C) | 28.3 | 2.3 | 16.2 | 27.2 | 28.5 | 29.6 | 34.8 | - |
| Chai Nat | Relative Humidity (%) | 74.1 | 7.6 | 43.0 | 69.0 | 75.0 | 79.0 | 97.0 | - |
| Chai Nat | All deaths | 7 | 3 | 1 | 5 | 7 | 9 | 21 | 40054 |
| Chaiyaphum | DTR (^o^C) | 9.8 | 2.7 | 0.4 | 8.0 | 9.6 | 11.5 | 18.9 | - |
| Chaiyaphum | Mean temperature (^o^C) | 27.8 | 2.7 | 13.8 | 26.5 | 28.0 | 29.5 | 35.4 | - |
| Chaiyaphum | Relative Humidity (%) | 69.0 | 10.8 | 33.0 | 61.0 | 69.0 | 77.0 | 97.0 | - |
| Chaiyaphum | All deaths | 20 | 6 | 1 | 16 | 19 | 23 | 50 | 107456 |
| Chanthaburi | DTR (^o^C) | 8.4 | 2.3 | 0.7 | 6.9 | 8.4 | 9.8 | 18.2 | - |
| Chanthaburi | Mean temperature (^o^C) | 27.7 | 1.4 | 20.7 | 27.0 | 27.9 | 28.7 | 31.8 | - |
| Chanthaburi | Relative Humidity (%) | 80.4 | 8.5 | 45.5 | 77.5 | 81.5 | 86.0 | 97.5 | - |
| Chanthaburi | All deaths | 10 | 3 | 1 | 7 | 9 | 12 | 24 | 52392 |
| Chiang Mai | DTR (^o^C) | 8.8 | 2.8 | 0.7 | 6.8 | 8.6 | 10.8 | 19.3 | - |
| Chiang Mai | Mean temperature (^o^C) | 23.3 | 2.8 | 7.0 | 21.8 | 23.6 | 25.0 | 31.3 | - |
| Chiang Mai | Relative Humidity (%) | 73.5 | 14.7 | 31.0 | 65.0 | 78.0 | 84.5 | 98.5 | - |
| Chiang Mai | All deaths | 35 | 7 | 1 | 30 | 35 | 39 | 72 | 190405 |
| Chiang Rai | DTR (^o^C) | 11.4 | 4.2 | 0.9 | 8.3 | 10.8 | 14.4 | 22.9 | - |
| Chiang Rai | Mean temperature (^o^C) | 25.1 | 3.2 | 8.9 | 23.0 | 25.8 | 27.4 | 33.3 | - |
| Chiang Rai | Relative Humidity (%) | 77.8 | 8.1 | 46.5 | 73.0 | 78.5 | 83.5 | 96.5 | - |
| Chiang Rai | All deaths | 22 | 5 | 1 | 18 | 21 | 25 | 45 | 118834 |
| Chon Buri | DTR (^o^C) | 7.3 | 1.6 | 1.9 | 6.2 | 7.2 | 8.3 | 13.4 | - |
| Chon Buri | Mean temperature (^o^C) | 28.6 | 1.5 | 20.0 | 27.8 | 28.8 | 29.6 | 33.0 | - |
| Chon Buri | Relative Humidity (%) | 76.0 | 7.3 | 45.6 | 72.4 | 76.6 | 80.8 | 96.2 | - |
| Chon Buri | All deaths | 24 | 6 | 1 | 20 | 24 | 28 | 53 | 131238 |
| Chumphon | DTR (^o^C) | 8.4 | 2.1 | 0.9 | 7.1 | 8.6 | 9.8 | 15.6 | - |
| Chumphon | Mean temperature (^o^C) | 27.4 | 1.4 | 20.7 | 26.5 | 27.5 | 28.4 | 33.2 | - |
| Chumphon | Relative Humidity (%) | 81.1 | 5.0 | 62.5 | 78.0 | 80.5 | 84.0 | 97.0 | - |
| Chumphon | All deaths | 8 | 3 | 1 | 6 | 7 | 9 | 23 | 41354 |
| Kalasin | DTR (^o^C) | 9.8 | 3.0 | 0.5 | 7.8 | 9.7 | 12.0 | 18.4 | - |
| Kalasin | Mean temperature (^o^C) | 27.6 | 3.0 | 13.6 | 25.9 | 27.9 | 29.6 | 36.6 | - |
| Kalasin | Relative Humidity (%) | 74.4 | 10.0 | 42.0 | 67.0 | 74.0 | 82.0 | 99.0 | - |
| Kalasin | All deaths | 17 | 5 | 1 | 14 | 17 | 20 | 39 | 92945 |
| Kamphaeng Phet | DTR (^o^C) | 10.1 | 3.0 | 0.9 | 8.0 | 9.8 | 12.0 | 19.7 | - |
| Kamphaeng Phet | Mean temperature (^o^C) | 28.0 | 2.5 | 13.8 | 26.8 | 28.1 | 29.4 | 36.1 | - |
| Kamphaeng Phet | Relative Humidity (%) | 75.0 | 10.1 | 36.0 | 68.0 | 76.0 | 83.0 | 99.0 | - |
| Kamphaeng Phet | All deaths | 12 | 4 | 1 | 9 | 12 | 14.25 | 37 | 65578 |
| Kanchanaburi | DTR (^o^C) | 11.3 | 3.1 | 2.6 | 9.0 | 11.2 | 13.7 | 22.4 | - |
| Kanchanaburi | Mean temperature (^o^C) | 28.0 | 2.2 | 17.8 | 26.9 | 28.0 | 29.4 | 34.5 | - |
| Kanchanaburi | Relative Humidity (%) | 73.9 | 8.9 | 41.0 | 67.5 | 74.5 | 81.0 | 95.5 | - |
| Kanchanaburi | All deaths | 12 | 4 | 1 | 9 | 12 | 15 | 32 | 66705 |
| Khon Kaen | DTR (^o^C) | 10.5 | 2.9 | 0.7 | 8.6 | 10.5 | 12.6 | 21.2 | - |
| Khon Kaen | Mean temperature (^o^C) | 27.3 | 2.9 | 12.8 | 25.9 | 27.6 | 29.2 | 35.9 | - |
| Khon Kaen | Relative Humidity (%) | 73.2 | 10.1 | 37.5 | 66.0 | 73.0 | 81.0 | 97.0 | - |
| Khon Kaen | All deaths | 33 | 8 | 1 | 28 | 33 | 38 | 77 | 180625 |
| Krabi | DTR (^o^C) | 7.9 | 2.2 | 1.7 | 6.5 | 7.9 | 9.4 | 20.4 | - |
| Krabi | Mean temperature (^o^C) | 27.7 | 1.1 | 21.1 | 27.0 | 27.7 | 28.5 | 31.7 | - |
| Krabi | Relative Humidity (%) | 81.9 | 6.6 | 60.0 | 78.0 | 82.5 | 86.5 | 98.5 | - |
| Krabi | All deaths | 5 | 2 | 1 | 3 | 5 | 7 | 16 | 28650 |
| Lampang | DTR (^o^C) | 12.0 | 3.9 | 1.0 | 9.1 | 11.4 | 14.8 | 23.9 | - |
| Lampang | Mean temperature (^o^C) | 27.0 | 2.9 | 10.8 | 25.6 | 27.3 | 28.8 | 35.3 | - |
| Lampang | Relative Humidity (%) | 73.2 | 12.0 | 34.7 | 66.3 | 76.0 | 82.0 | 95.7 | - |
| Lampang | All deaths | 18 | 5 | 1 | 14 | 17 | 21 | 47 | 96275 |
| Lamphun | DTR (^o^C) | 11.8 | 4.1 | 0.7 | 8.8 | 11.0 | 14.6 | 24.7 | - |
| Lamphun | Mean temperature (^o^C) | 26.9 | 3.1 | 10.9 | 25.1 | 27.2 | 28.8 | 35.6 | - |
| Lamphun | Relative Humidity (%) | 72.6 | 11.7 | 35.0 | 66.0 | 75.0 | 81.0 | 98.0 | - |
| Lamphun | All deaths | 10 | 3 | 1 | 7 | 9 | 12 | 26 | 52424 |
| Loei | DTR (^o^C) | 11.3 | 3.9 | 0.4 | 8.7 | 10.9 | 13.9 | 23.4 | - |
| Loei | Mean temperature (^o^C) | 26.1 | 3.0 | 12.0 | 24.7 | 26.6 | 28.1 | 33.7 | - |
| Loei | Relative Humidity (%) | 76.6 | 9.3 | 44.5 | 70.5 | 77.5 | 83.5 | 96.0 | - |
| Loei | All deaths | 11 | 4 | 1 | 8 | 10 | 13 | 30 | 57890 |
| Lop Buri | DTR (^o^C) | 10.3 | 2.5 | 1.1 | 8.7 | 10.2 | 11.8 | 20.5 | - |
| Lop Buri | Mean temperature (^o^C) | 28.5 | 2.3 | 15.9 | 27.4 | 28.6 | 29.9 | 35.2 | - |
| Lop Buri | Relative Humidity (%) | 72.8 | 9.5 | 34.5 | 66.0 | 73.5 | 80.0 | 97.0 | - |
| Lop Buri | All deaths | 15 | 4 | 1 | 12 | 15 | 18 | 37 | 81014 |
| Mae Hong Son | DTR (^o^C) | 12.7 | 4.4 | 1.4 | 9.3 | 12.0 | 15.8 | 26.1 | - |
| Mae Hong Son | Mean temperature (^o^C) | 26.3 | 3.1 | 15.2 | 24.4 | 26.7 | 28.1 | 35.2 | - |
| Mae Hong Son | Relative Humidity (%) | 76.3 | 10.7 | 42.5 | 70.5 | 80.0 | 84.0 | 95.5 | - |
| Mae Hong Son | All deaths | 3 | 2 | 1 | 2 | 3 | 4 | 14 | 16637 |
| Maha Sarakham | DTR (^o^C) | 10.8 | 2.8 | 0.6 | 8.9 | 10.6 | 12.6 | 20.8 | - |
| Maha Sarakham | Mean temperature (^o^C) | 27.8 | 2.9 | 13.1 | 26.3 | 28.2 | 29.6 | 36.1 | - |
| Maha Sarakham | Relative Humidity (%) | 73.5 | 8.5 | 45.0 | 68.0 | 73.0 | 79.0 | 99.0 | - |
| Maha Sarakham | All deaths | 16 | 5 | 1 | 13 | 16 | 19 | 36 | 89456 |
| Mukdahan | DTR (^o^C) | 10.1 | 2.9 | 0.7 | 8.2 | 9.9 | 12.0 | 21.2 | - |
| Mukdahan | Mean temperature (^o^C) | 27.2 | 3.2 | 13.7 | 25.6 | 27.6 | 29.2 | 36.6 | - |
| Mukdahan | Relative Humidity (%) | 72.2 | 10.0 | 36.0 | 65.0 | 71.0 | 80.0 | 98.0 | - |
| Mukdahan | All deaths | 5 | 2 | 1 | 4 | 5 | 7 | 16 | 28791 |
| Nakhon Nayok | DTR (^o^C) | 6.9 | 2.2 | 1.1 | 5.7 | 7.2 | 8.5 | 14.2 | - |
| Nakhon Nayok | Mean temperature (^o^C) | 21.0 | 1.8 | 12.2 | 20.0 | 21.2 | 22.2 | 24.9 | - |
| Nakhon Nayok | Relative Humidity (%) | 88.0 | 10.2 | 45.0 | 83.0 | 91.0 | 96.0 | 100.0 | - |
| Nakhon Nayok | All deaths | 6 | 3 | 1 | 5 | 6 | 8 | 16 | 7031 |
| Nakhon Pathom | DTR (^o^C) | 10.3 | 2.5 | 1.7 | 8.7 | 10.2 | 11.8 | 20.1 | - |
| Nakhon Pathom | Mean temperature (^o^C) | 28.0 | 2.3 | 16.2 | 27.0 | 28.3 | 29.5 | 33.8 | - |
| Nakhon Pathom | Relative Humidity (%) | 78.2 | 6.6 | 41.0 | 74.0 | 78.0 | 83.0 | 99.0 | - |
| Nakhon Pathom | All deaths | 14 | 4 | 1 | 11 | 14 | 17 | 35 | 79145 |
| Nakhon Phanom | DTR (^o^C) | 10.1 | 3.4 | 0.8 | 7.7 | 9.9 | 12.6 | 20.5 | - |
| Nakhon Phanom | Mean temperature (^o^C) | 26.4 | 3.1 | 13.3 | 24.9 | 27.0 | 28.5 | 34.8 | - |
| Nakhon Phanom | Relative Humidity (%) | 74.6 | 10.2 | 38.0 | 67.0 | 74.0 | 83.0 | 97.5 | - |
| Nakhon Phanom | All deaths | 11 | 4 | 1 | 9 | 11 | 14 | 29 | 61780 |
| Nakhon Ratchasima | DTR (^o^C) | 9.9 | 2.6 | 1.3 | 8.2 | 9.7 | 11.6 | 20.5 | - |
| Nakhon Ratchasima | Mean temperature (^o^C) | 27.2 | 2.5 | 13.9 | 26.0 | 27.5 | 28.8 | 33.4 | - |
| Nakhon Ratchasima | Relative Humidity (%) | 73.0 | 8.9 | 41.0 | 66.7 | 73.0 | 79.7 | 97.7 | - |
| Nakhon Ratchasima | All deaths | 44 | 10 | 1 | 37 | 43 | 50 | 88 | 238325 |
| Nakhon Sawan | DTR (^o^C) | 10.2 | 2.5 | 0.8 | 8.6 | 10.1 | 11.7 | 20.8 | - |
| Nakhon Sawan | Mean temperature (^o^C) | 28.5 | 2.4 | 15.7 | 27.3 | 28.6 | 29.9 | 35.7 | - |
| Nakhon Sawan | Relative Humidity (%) | 72.8 | 9.9 | 34.5 | 65.5 | 73.5 | 81.0 | 97.0 | - |
| Nakhon Sawan | All deaths | 20 | 6 | 1 | 16 | 20 | 24 | 51 | 111512 |
| Nakhon Si Thammarat | DTR (^o^C) | 9.6 | 2.5 | 1.2 | 8.3 | 9.7 | 10.9 | 22.8 | - |
| Nakhon Si Thammarat | Mean temperature (^o^C) | 27.4 | 1.3 | 23.6 | 26.6 | 27.4 | 28.3 | 31.5 | - |
| Nakhon Si Thammarat | Relative Humidity (%) | 83.6 | 4.9 | 67.0 | 80.0 | 83.3 | 86.7 | 98.7 | - |
| Nakhon Si Thammarat | All deaths | 23 | 6 | 1 | 19 | 23 | 27 | 46 | 124851 |
| Nan | DTR (^o^C) | 12.0 | 4.3 | 1.0 | 8.8 | 11.5 | 15.2 | 25.3 | - |
| Nan | Mean temperature (^o^C) | 26.1 | 3.0 | 9.3 | 24.3 | 26.7 | 28.1 | 33.4 | - |
| Nan | Relative Humidity (%) | 78.1 | 7.6 | 52.5 | 73.5 | 79.0 | 83.5 | 97.3 | - |
| Nan | All deaths | 8 | 3 | 1 | 6 | 8 | 10 | 25 | 44907 |
| Narathiwat | DTR (^o^C) | 8.7 | 2.0 | 1.1 | 7.5 | 8.8 | 10.1 | 16.0 | - |
| Narathiwat | Mean temperature (^o^C) | 26.9 | 2.6 | 13.1 | 26.5 | 27.4 | 28.3 | 31.1 | - |
| Narathiwat | Relative Humidity (%) | 80.5 | 5.0 | 58.0 | 77.0 | 80.0 | 83.0 | 97.0 | - |
| Narathiwat | All deaths | 11 | 4 | 1 | 8 | 11 | 14 | 28 | 60713 |
| Nong Bua Lam Phu | DTR (^o^C) | 10.8 | 3.4 | 1.2 | 8.5 | 10.5 | 13.0 | 22.3 | - |
| Nong Bua Lam Phu | Mean temperature (^o^C) | 27.2 | 3.0 | 11.5 | 25.6 | 27.4 | 29.1 | 35.9 | - |
| Nong Bua Lam Phu | Relative Humidity (%) | 73.7 | 11.1 | 38.0 | 65.0 | 74.5 | 82.0 | 98.0 | - |
| Nong Bua Lam Phu | All deaths | 8 | 3 | 1 | 6 | 8 | 10 | 22 | 23451 |
| Nong Khai | DTR (^o^C) | 10.0 | 2.9 | 0.4 | 8.1 | 10.0 | 12.1 | 19.7 | - |
| Nong Khai | Mean temperature (^o^C) | 27.3 | 3.1 | 11.8 | 25.8 | 27.7 | 29.3 | 35.3 | - |
| Nong Khai | Relative Humidity (%) | 72.7 | 10.2 | 42.0 | 65.0 | 72.0 | 81.0 | 98.0 | - |
| Nong Khai | All deaths | 10 | 4 | 1 | 7 | 9 | 12 | 25 | 52639 |
| Pathum Thani | DTR (^o^C) | 9.8 | 2.1 | 0.8 | 8.5 | 9.8 | 11.2 | 16.8 | - |
| Pathum Thani | Mean temperature (^o^C) | 29.1 | 2.0 | 16.5 | 28.1 | 29.3 | 30.4 | 35.6 | - |
| Pathum Thani | Relative Humidity (%) | 74.2 | 7.9 | 45.0 | 69.0 | 74.0 | 80.0 | 97.0 | - |
| Pathum Thani | All deaths | 15 | 5 | 1 | 12 | 15 | 19 | 37 | 84603 |
| Pattani | DTR (^o^C) | 9.0 | 2.0 | 0.8 | 8.0 | 9.2 | 10.3 | 16.0 | - |
| Pattani | Mean temperature (^o^C) | 27.7 | 1.3 | 23.4 | 26.9 | 27.8 | 28.6 | 31.9 | - |
| Pattani | Relative Humidity (%) | 81.3 | 5.2 | 59.0 | 78.0 | 81.0 | 85.0 | 98.0 | - |
| Pattani | All deaths | 10 | 4 | 1 | 8 | 10 | 13 | 27 | 57143 |
| Phangnga | DTR (^o^C) | 7.8 | 2.0 | 1.2 | 6.6 | 7.8 | 8.9 | 16.1 | - |
| Phangnga | Mean temperature (^o^C) | 27.4 | 1.1 | 22.2 | 26.7 | 27.4 | 28.2 | 30.7 | - |
| Phangnga | Relative Humidity (%) | 83.5 | 6.6 | 50.0 | 80.0 | 84.0 | 88.0 | 99.0 | - |
| Phangnga | All deaths | 4 | 2 | 1 | 2 | 4 | 5 | 16 | 20113 |
| Phatthalung | DTR (^o^C) | 7.9 | 2.1 | 0.4 | 6.6 | 8.1 | 9.4 | 15.7 | - |
| Phatthalung | Mean temperature (^o^C) | 27.9 | 1.3 | 23.4 | 27.1 | 27.9 | 28.7 | 32.7 | - |
| Phatthalung | Relative Humidity (%) | 82.5 | 5.6 | 62.0 | 79.0 | 82.0 | 86.0 | 100.0 | - |
| Phatthalung | All deaths | 8 | 3 | 1 | 5 | 7 | 9 | 21 | 41026 |
| Phayao | DTR (^o^C) | 11.3 | 3.9 | 0.7 | 8.5 | 10.6 | 14.0 | 23.1 | - |
| Phayao | Mean temperature (^o^C) | 25.8 | 3.2 | 9.0 | 23.9 | 26.3 | 27.9 | 34.9 | - |
| Phayao | Relative Humidity (%) | 76.6 | 10.0 | 37.0 | 71.0 | 79.0 | 83.0 | 98.0 | - |
| Phayao | All deaths | 10 | 3 | 1 | 8 | 10 | 12 | 27 | 54750 |
| Phetchabun | DTR (^o^C) | 11.1 | 3.0 | 1.1 | 9.0 | 10.9 | 13.2 | 21.4 | - |
| Phetchabun | Mean temperature (^o^C) | 27.9 | 2.4 | 15.2 | 26.8 | 28.0 | 29.3 | 34.8 | - |
| Phetchabun | Relative Humidity (%) | 72.3 | 9.7 | 39.7 | 64.7 | 72.7 | 80.3 | 96.0 | - |
| Phetchabun | All deaths | 18 | 5 | 1 | 14 | 17 | 21 | 52 | 95728 |
| Phetchaburi | DTR (^o^C) | 8.0 | 1.8 | 0.7 | 6.9 | 7.9 | 9.0 | 15.6 | - |
| Phetchaburi | Mean temperature (^o^C) | 28.5 | 1.8 | 18.9 | 27.6 | 28.7 | 29.7 | 33.8 | - |
| Phetchaburi | Relative Humidity (%) | 76.6 | 6.2 | 49.0 | 73.0 | 77.0 | 80.0 | 97.0 | - |
| Phetchaburi | All deaths | 8 | 3 | 1 | 6 | 8 | 10 | 22 | 45310 |
| Phichit | DTR (^o^C) | 10.0 | 2.6 | 1.3 | 8.2 | 9.9 | 11.8 | 19.0 | - |
| Phichit | Mean temperature (^o^C) | 28.1 | 2.4 | 14.7 | 27.0 | 28.3 | 29.6 | 35.4 | - |
| Phichit | Relative Humidity (%) | 76.8 | 7.7 | 44.0 | 72.0 | 78.0 | 82.0 | 96.0 | - |
| Phichit | All deaths | 10 | 4 | 1 | 8 | 10 | 13 | 33 | 57161 |
| Phitsanulok | DTR (^o^C) | 10.1 | 2.5 | 1.8 | 8.5 | 10.0 | 11.7 | 18.6 | - |
| Phitsanulok | Mean temperature (^o^C) | 28.1 | 2.4 | 14.3 | 27.0 | 28.3 | 29.6 | 35.7 | - |
| Phitsanulok | Relative Humidity (%) | 74.1 | 8.8 | 42.0 | 68.0 | 74.0 | 81.0 | 97.0 | - |
| Phitsanulok | All deaths | 17 | 5 | 1 | 13 | 16 | 20 | 44 | 90958 |
| Phra Nakhon Si Ayutthaya | DTR (^o^C) | 10.9 | 2.4 | 1.3 | 9.4 | 10.8 | 12.3 | 20.8 | - |
| Phra Nakhon Si Ayutthaya | Mean temperature (^o^C) | 28.5 | 2.1 | 16.3 | 27.5 | 28.7 | 29.7 | 34.4 | - |
| Phra Nakhon Si Ayutthaya | Relative Humidity (%) | 75.1 | 8.1 | 36.0 | 70.0 | 76.0 | 81.0 | 96.0 | - |
| Phra Nakhon Si Ayutthaya | All deaths | 15 | 4 | 1 | 12 | 15 | 18 | 36 | 82514 |
| Phrae | DTR (^o^C) | 11.3 | 3.8 | 0.9 | 8.5 | 10.9 | 14.0 | 22.7 | - |
| Phrae | Mean temperature (^o^C) | 27.1 | 3.0 | 10.9 | 25.7 | 27.4 | 28.9 | 36.2 | - |
| Phrae | Relative Humidity (%) | 75.3 | 9.6 | 43.0 | 70.0 | 77.0 | 82.0 | 97.0 | - |
| Phrae | All deaths | 11 | 4 | 1 | 8 | 11 | 13 | 30 | 59988 |
| Phuket | DTR (^o^C) | 8.0 | 1.7 | 1.7 | 7.0 | 8.1 | 9.0 | 13.6 | - |
| Phuket | Mean temperature (^o^C) | 28.5 | 1.1 | 23.9 | 27.8 | 28.5 | 29.3 | 32.3 | - |
| Phuket | Relative Humidity (%) | 78.2 | 6.1 | 57.0 | 74.0 | 78.5 | 82.5 | 95.5 | - |
| Phuket | All deaths | 5 | 2 | 1 | 3 | 5 | 6 | 16 | 26048 |
| Prachin Buri | DTR (^o^C) | 9.8 | 2.4 | 1.1 | 8.3 | 9.7 | 11.5 | 18.1 | - |
| Prachin Buri | Mean temperature (^o^C) | 28.5 | 1.9 | 18.3 | 27.5 | 28.5 | 29.7 | 33.9 | - |
| Prachin Buri | Relative Humidity (%) | 75.7 | 9.0 | 42.5 | 69.5 | 76.5 | 82.5 | 96.0 | - |
| Prachin Buri | All deaths | 8 | 3 | 1 | 6 | 8 | 10 | 21 | 45629 |
| Prachuab Khiri Khan | DTR (^o^C) | 8.9 | 1.9 | 0.8 | 7.7 | 9.0 | 10.2 | 15.8 | - |
| Prachuab Khiri Khan | Mean temperature (^o^C) | 27.8 | 1.7 | 19.6 | 26.9 | 27.9 | 28.9 | 32.6 | - |
| Prachuab Khiri Khan | Relative Humidity (%) | 75.9 | 6.5 | 44.0 | 72.0 | 76.0 | 80.0 | 98.0 | - |
| Prachuab Khiri Khan | All deaths | 8 | 3 | 1 | 6 | 7 | 10 | 22 | 42254 |
| Ranong | DTR (^o^C) | 8.2 | 2.5 | 0.6 | 6.5 | 8.3 | 10.0 | 21.4 | - |
| Ranong | Mean temperature (^o^C) | 27.5 | 1.3 | 21.7 | 26.6 | 27.5 | 28.4 | 32.1 | - |
| Ranong | Relative Humidity (%) | 79.1 | 7.9 | 56.0 | 73.0 | 80.0 | 85.0 | 95.0 | - |
| Ranong | All deaths | 2 | 1 | 1 | 1 | 2 | 3 | 10 | 11601 |
| Ratchaburi | DTR (^o^C) | 9.4 | 2.6 | 0.1 | 7.6 | 9.4 | 11.1 | 18.9 | - |
| Ratchaburi | Mean temperature (^o^C) | 27.9 | 2.0 | 17.0 | 26.9 | 28.1 | 29.2 | 33.9 | - |
| Ratchaburi | Relative Humidity (%) | 78.1 | 7.1 | 53.0 | 73.0 | 78.0 | 83.0 | 100.0 | - |
| Ratchaburi | All deaths | 16 | 5 | 1 | 13 | 16 | 19 | 40 | 88017 |
| Rayong | DTR (^o^C) | 6.9 | 2.1 | 0.6 | 5.3 | 6.7 | 8.3 | 16.7 | - |
| Rayong | Mean temperature (^o^C) | 28.3 | 1.6 | 21.0 | 27.4 | 28.4 | 29.4 | 33.1 | - |
| Rayong | Relative Humidity (%) | 77.4 | 7.1 | 43.0 | 74.5 | 78.0 | 82.0 | 95.5 | - |
| Rayong | All deaths | 10 | 4 | 1 | 8 | 10 | 13 | 27 | 55964 |
| Roi Et | DTR (^o^C) | 9.8 | 3.0 | 0.9 | 7.8 | 9.7 | 12.0 | 19.1 | - |
| Roi Et | Mean temperature (^o^C) | 27.4 | 3.0 | 14.0 | 25.9 | 27.8 | 29.3 | 34.9 | - |
| Roi Et | Relative Humidity (%) | 73.7 | 9.5 | 36.5 | 67.0 | 73.5 | 81.0 | 97.5 | - |
| Roi Et | All deaths | 22 | 6 | 1 | 18 | 22 | 26 | 48 | 122523 |
| Sa Kaeo | DTR (^o^C) | 9.8 | 2.4 | 1.3 | 8.2 | 9.7 | 11.5 | 22.6 | - |
| Sa Kaeo | Mean temperature (^o^C) | 28.3 | 2.0 | 19.2 | 27.2 | 28.3 | 29.5 | 34.1 | - |
| Sa Kaeo | Relative Humidity (%) | 76.5 | 8.7 | 49.5 | 70.0 | 77.0 | 83.5 | 96.5 | - |
| Sa Kaeo | All deaths | 8 | 3 | 1 | 6 | 8 | 10 | 24 | 44247 |
| Sakon Nakhon | DTR (^o^C) | 10.3 | 3.3 | 0.9 | 7.9 | 10.0 | 12.8 | 20.3 | - |
| Sakon Nakhon | Mean temperature (^o^C) | 26.5 | 3.3 | 13.0 | 24.9 | 27.1 | 28.6 | 35.8 | - |
| Sakon Nakhon | Relative Humidity (%) | 74.9 | 9.2 | 37.5 | 68.5 | 74.5 | 82.0 | 97.0 | - |
| Sakon Nakhon | All deaths | 18 | 5 | 1 | 15 | 18 | 21 | 39 | 99218 |
| Samut Prakan | DTR (^o^C) | 5.7 | 1.6 | 0.6 | 4.6 | 5.6 | 6.7 | 14.7 | - |
| Samut Prakan | Mean temperature (^o^C) | 28.8 | 1.7 | 18.5 | 28.1 | 29.1 | 29.8 | 33.4 | - |
| Samut Prakan | Relative Humidity (%) | 72.1 | 7.1 | 36.0 | 68.0 | 73.0 | 77.0 | 93.7 | - |
| Samut Prakan | All deaths | 18 | 6 | 1 | 14 | 18 | 22 | 48 | 100721 |
| Samut Songkhram | DTR (^o^C) | 7.3 | 2.2 | 1.3 | 5.7 | 7.1 | 8.7 | 14.8 | - |
| Samut Songkhram | Mean temperature (^o^C) | 28.4 | 1.8 | 20.5 | 27.4 | 28.6 | 29.6 | 32.7 | - |
| Samut Songkhram | Relative Humidity (%) | 75.8 | 6.6 | 57.0 | 72.0 | 76.0 | 80.0 | 95.0 | - |
| Samut Songkhram | All deaths | 4 | 2 | 1 | 3 | 4 | 6 | 13 | 4673 |
| Satun | DTR (^o^C) | 8.8 | 2.1 | 1.4 | 7.7 | 8.8 | 9.9 | 17.1 | - |
| Satun | Mean temperature (^o^C) | 28.0 | 1.2 | 23.8 | 27.3 | 28.1 | 28.8 | 32.0 | - |
| Satun | Relative Humidity (%) | 79.5 | 7.2 | 55.0 | 75.0 | 80.0 | 84.0 | 96.0 | - |
| Satun | All deaths | 4 | 2 | 1 | 2 | 4 | 5 | 13 | 20544 |
| Si Sa Ket | DTR (^o^C) | 10.1 | 3.1 | 0.2 | 7.8 | 9.8 | 12.3 | 20.0 | - |
| Si Sa Ket | Mean temperature (^o^C) | 27.5 | 2.7 | 16.8 | 26.3 | 27.9 | 29.2 | 34.4 | - |
| Si Sa Ket | Relative Humidity (%) | 74.4 | 8.6 | 46.0 | 68.0 | 74.0 | 81.0 | 98.0 | - |
| Si Sa Ket | All deaths | 22 | 6 | 1 | 18 | 22 | 26 | 43 | 121547 |
| Songkhla | DTR (^o^C) | 8.8 | 1.8 | 1.2 | 7.8 | 9.0 | 10.0 | 17.0 | - |
| Songkhla | Mean temperature (^o^C) | 27.7 | 1.2 | 23.3 | 27.0 | 27.8 | 28.6 | 31.7 | - |
| Songkhla | Relative Humidity (%) | 80.2 | 5.3 | 63.0 | 76.3 | 80.0 | 83.8 | 97.5 | - |
| Songkhla | All deaths | 22 | 5 | 1 | 18 | 22 | 26 | 44 | 121129 |
| Sukhothai | DTR (^o^C) | 10.3 | 2.8 | 1.3 | 8.4 | 10.1 | 12.3 | 19.3 | - |
| Sukhothai | Mean temperature (^o^C) | 28.1 | 2.6 | 13.1 | 26.8 | 28.3 | 29.6 | 36.3 | - |
| Sukhothai | Relative Humidity (%) | 76.9 | 8.6 | 44.0 | 72.0 | 77.5 | 83.0 | 99.0 | - |
| Sukhothai | All deaths | 12 | 4 | 1 | 9 | 11 | 14 | 31 | 63074 |
| Suphan Buri | DTR (^o^C) | 10.3 | 2.3 | 1.6 | 8.8 | 10.3 | 11.8 | 21.3 | - |
| Suphan Buri | Mean temperature (^o^C) | 28.5 | 2.3 | 16.5 | 27.4 | 28.7 | 29.9 | 34.8 | - |
| Suphan Buri | Relative Humidity (%) | 74.8 | 7.0 | 41.5 | 70.0 | 74.5 | 79.5 | 97.5 | - |
| Suphan Buri | All deaths | 17 | 5 | 1 | 14 | 17 | 20 | 37 | 92186 |
| Surat Thani | DTR (^o^C) | 8.9 | 1.9 | 1.3 | 7.9 | 9.1 | 10.2 | 17.0 | - |
| Surat Thani | Mean temperature (^o^C) | 27.5 | 1.2 | 23.2 | 26.7 | 27.5 | 28.3 | 31.5 | - |
| Surat Thani | Relative Humidity (%) | 82.8 | 4.8 | 67.3 | 79.3 | 82.8 | 86.0 | 97.3 | - |
| Surat Thani | All deaths | 14 | 4 | 1 | 11 | 14 | 17 | 35 | 78117 |
| Surin | DTR (^o^C) | 10.5 | 2.7 | 1.4 | 8.6 | 10.3 | 12.4 | 21.3 | - |
| Surin | Mean temperature (^o^C) | 27.6 | 2.7 | 16.8 | 26.4 | 28.0 | 29.3 | 34.5 | - |
| Surin | Relative Humidity (%) | 73.7 | 8.3 | 47.7 | 67.7 | 73.7 | 80.0 | 96.3 | - |
| Surin | All deaths | 22 | 6 | 1 | 18 | 22 | 26 | 49 | 120784 |
| Tak | DTR (^o^C) | 11.1 | 3.8 | 2.0 | 8.1 | 10.5 | 14.0 | 23.2 | - |
| Tak | Mean temperature (^o^C) | 25.9 | 2.6 | 12.7 | 24.7 | 26.0 | 27.4 | 33.3 | - |
| Tak | Relative Humidity (%) | 74.6 | 10.4 | 42.0 | 68.6 | 76.8 | 82.2 | 95.2 | - |
| Tak | All deaths | 7 | 3 | 1 | 5 | 7 | 9 | 20 | 40711 |
| Trang | DTR (^o^C) | 9.5 | 2.4 | 0.5 | 8.1 | 9.6 | 11.0 | 18.3 | - |
| Trang | Mean temperature (^o^C) | 27.7 | 1.3 | 23.5 | 26.8 | 27.8 | 28.6 | 32.1 | - |
| Trang | Relative Humidity (%) | 81.3 | 7.5 | 58.0 | 76.0 | 82.0 | 87.0 | 98.0 | - |
| Trang | All deaths | 9 | 3 | 1 | 7 | 9 | 11 | 26 | 49401 |
| Trat | DTR (^o^C) | 8.1 | 2.1 | 1.0 | 7.1 | 8.3 | 9.4 | 16.5 | - |
| Trat | Mean temperature (^o^C) | 27.7 | 1.2 | 22.2 | 26.9 | 27.8 | 28.6 | 31.4 | - |
| Trat | Relative Humidity (%) | 81.7 | 8.5 | 45.0 | 78.0 | 83.0 | 87.0 | 98.0 | - |
| Trat | All deaths | 4 | 2 | 1 | 2 | 3 | 5 | 14 | 18728 |
| Ubon Ratchathani | DTR (^o^C) | 10.9 | 3.1 | 0.6 | 8.7 | 10.7 | 13.1 | 19.9 | - |
| Ubon Ratchathani | Mean temperature (^o^C) | 27.5 | 2.6 | 16.5 | 26.2 | 27.7 | 29.2 | 34.8 | - |
| Ubon Ratchathani | Relative Humidity (%) | 73.3 | 9.3 | 43.0 | 66.3 | 73.0 | 80.5 | 98.0 | - |
| Ubon Ratchathani | All deaths | 29 | 7 | 1 | 25 | 29 | 33 | 59 | 159424 |
| Udon Thani | DTR (^o^C) | 10.5 | 3.3 | 0.3 | 8.1 | 10.3 | 12.9 | 21.3 | - |
| Udon Thani | Mean temperature (^o^C) | 27.2 | 3.1 | 11.8 | 25.7 | 27.7 | 29.2 | 35.9 | - |
| Udon Thani | Relative Humidity (%) | 71.6 | 10.1 | 38.0 | 65.0 | 71.0 | 79.0 | 95.0 | - |
| Udon Thani | All deaths | 25 | 6 | 1 | 20 | 24 | 29 | 55 | 134737 |
| Uthai Thani | DTR (^o^C) | 10.6 | 2.7 | 1.5 | 9.0 | 10.7 | 12.4 | 18.4 | - |
| Uthai Thani | Mean temperature (^o^C) | 29.6 | 2.4 | 20.4 | 28.2 | 29.7 | 31.2 | 35.1 | - |
| Uthai Thani | Relative Humidity (%) | 72.1 | 10.2 | 45.0 | 65.0 | 72.0 | 80.0 | 98.0 | - |
| Uthai Thani | All deaths | 7 | 3 | 1 | 5 | 7 | 9 | 17 | 7433 |
| Uttaradit | DTR (^o^C) | 10.8 | 2.9 | 1.4 | 8.9 | 10.8 | 12.7 | 19.9 | - |
| Uttaradit | Mean temperature (^o^C) | 28.2 | 2.7 | 12.1 | 26.9 | 28.3 | 29.8 | 36.8 | - |
| Uttaradit | Relative Humidity (%) | 71.4 | 9.6 | 39.0 | 64.0 | 72.0 | 79.0 | 95.0 | - |
| Uttaradit | All deaths | 10 | 4 | 1 | 7 | 10 | 12 | 26 | 53426 |
| Yala | DTR (^o^C) | 9.7 | 2.4 | 0.8 | 8.4 | 9.9 | 11.2 | 19.0 | - |
| Yala | Mean temperature (^o^C) | 27.4 | 1.4 | 22.3 | 26.5 | 27.4 | 28.3 | 32.0 | - |
| Yala | Relative Humidity (%) | 81.2 | 5.9 | 54.0 | 77.0 | 81.0 | 85.0 | 99.0 | - |
| Yala | All deaths | 7 | 3 | 1 | 5 | 7 | 9 | 20 | 37815 |
| Yasothon | DTR (^o^C) | 10.7 | 3.0 | 2.0 | 8.8 | 10.6 | 12.8 | 18.6 | - |
| Yasothon | Mean temperature (^o^C) | 27.6 | 2.9 | 16.4 | 25.7 | 28.2 | 29.6 | 35.0 | - |
| Yasothon | Relative Humidity (%) | 76.0 | 10.4 | 52.0 | 67.0 | 75.0 | 85.0 | 100.0 | - |
| Yasothon | All deaths | 11 | 4 | 1 | 9 | 11 | 13 | 23 | 8114 |

**Table S3** The province-specific BLUP estimates of all-cause mortality associated with different DTR percentiles and lag periods relative to the MM-DTR

| **Provinces** | **Lag** | **MM-DTR** | **Percentile** | **DTR** | **RR and 95% CI** | | |
| --- | --- | --- | --- | --- | --- | --- | --- |
|  |  |  |  |  | **RR** | **Lower** | **Upper** |
| Amnat Charoen | 0-7 | 10.2 | 1st | 2.1 | 1.1864 | 1.0415 | 1.3515 |
| Amnat Charoen | 0-7 | 10.2 | 10th | 6.2 | 1.0430 | 1.0009 | 1.0869 |
| Amnat Charoen | 0-7 | 10.2 | 90th | 13.6 | 1.0177 | 0.9783 | 1.0586 |
| Amnat Charoen | 0-7 | 10.2 | 99th | 16.3 | 1.0471 | 0.9916 | 1.1057 |
| Amnat Charoen | 0-14 | 11.4 | 1st | 2.1 | 1.2641 | 1.0756 | 1.4856 |
| Amnat Charoen | 0-14 | 11.4 | 10th | 6.2 | 1.0716 | 1.0038 | 1.1440 |
| Amnat Charoen | 0-14 | 11.4 | 90th | 13.6 | 1.0073 | 0.9775 | 1.0381 |
| Amnat Charoen | 0-14 | 11.4 | 99th | 16.3 | 1.0292 | 0.9873 | 1.0728 |
| Amnat Charoen | 0-21 | 13.8 | 1st | 2.1 | 1.3366 | 1.0636 | 1.6796 |
| Amnat Charoen | 0-21 | 13.8 | 10th | 6.2 | 1.1163 | 0.9900 | 1.2586 |
| Amnat Charoen | 0-21 | 13.8 | 90th | 13.6 | 1.0000 | 0.9975 | 1.0026 |
| Amnat Charoen | 0-21 | 13.8 | 99th | 16.3 | 1.0068 | 0.9901 | 1.0238 |
| Bangkok | 0-7 | 9.5 | 1st | 3.4 | 1.0731 | 1.0288 | 1.1193 |
| Bangkok | 0-7 | 9.5 | 10th | 5.9 | 1.0262 | 1.0098 | 1.0427 |
| Bangkok | 0-7 | 9.5 | 90th | 9.7 | 1.0000 | 0.9992 | 1.0009 |
| Bangkok | 0-7 | 9.5 | 99th | 11.7 | 1.0070 | 0.9963 | 1.0179 |
| Bangkok | 0-14 | 10.4 | 1st | 3.4 | 1.1106 | 1.0543 | 1.1700 |
| Bangkok | 0-14 | 10.4 | 10th | 5.9 | 1.0441 | 1.0216 | 1.0671 |
| Bangkok | 0-14 | 10.4 | 90th | 9.7 | 1.0007 | 0.9962 | 1.0054 |
| Bangkok | 0-14 | 10.4 | 99th | 11.7 | 1.0027 | 0.9937 | 1.0118 |
| Bangkok | 0-21 | 12.2 | 1st | 3.4 | 1.1351 | 1.0556 | 1.2205 |
| Bangkok | 0-21 | 12.2 | 10th | 5.9 | 1.0659 | 1.0258 | 1.1074 |
| Bangkok | 0-21 | 12.2 | 90th | 9.7 | 1.0085 | 0.9859 | 1.0317 |
| Bangkok | 0-21 | 12.2 | 99th | 11.7 | 1.0003 | 0.9959 | 1.0047 |
| Bueng Kan | 0-7 | 9.9 | 1st | 2.2 | 1.2243 | 1.0779 | 1.3906 |
| Bueng Kan | 0-7 | 9.9 | 10th | 5.7 | 1.0628 | 1.0160 | 1.1118 |
| Bueng Kan | 0-7 | 9.9 | 90th | 15.3 | 1.0474 | 0.9929 | 1.1049 |
| Bueng Kan | 0-7 | 9.9 | 99th | 18.6 | 1.0982 | 1.0290 | 1.1721 |
| Bueng Kan | 0-14 | 10.4 | 1st | 2.2 | 1.3009 | 1.1086 | 1.5264 |
| Bueng Kan | 0-14 | 10.4 | 10th | 5.7 | 1.0883 | 1.0261 | 1.1541 |
| Bueng Kan | 0-14 | 10.4 | 90th | 15.3 | 1.0323 | 0.9763 | 1.0914 |
| Bueng Kan | 0-14 | 10.4 | 99th | 18.6 | 1.0656 | 1.0055 | 1.1292 |
| Bueng Kan | 0-21 | 11.8 | 1st | 2.2 | 1.3825 | 1.0977 | 1.7411 |
| Bueng Kan | 0-21 | 11.8 | 10th | 5.7 | 1.1255 | 1.0257 | 1.2349 |
| Bueng Kan | 0-21 | 11.8 | 90th | 15.3 | 1.0078 | 0.9610 | 1.0569 |
| Bueng Kan | 0-21 | 11.8 | 99th | 18.6 | 1.0200 | 0.9747 | 1.0674 |
| Buri Ram | 0-7 | 9.7 | 1st | 4.1 | 1.1439 | 1.0855 | 1.2054 |
| Buri Ram | 0-7 | 9.7 | 10th | 7.6 | 1.0170 | 1.0033 | 1.0309 |
| Buri Ram | 0-7 | 9.7 | 90th | 14.5 | 1.0596 | 1.0260 | 1.0942 |
| Buri Ram | 0-7 | 9.7 | 99th | 17.6 | 1.1351 | 1.0923 | 1.1797 |
| Buri Ram | 0-14 | 11.2 | 1st | 4.1 | 1.1750 | 1.1023 | 1.2526 |
| Buri Ram | 0-14 | 11.2 | 10th | 7.6 | 1.0391 | 1.0066 | 1.0727 |
| Buri Ram | 0-14 | 11.2 | 90th | 14.5 | 1.0236 | 0.9978 | 1.0501 |
| Buri Ram | 0-14 | 11.2 | 99th | 17.6 | 1.0813 | 1.0486 | 1.1151 |
| Buri Ram | 0-21 | 14.5 | 1st | 4.1 | 1.2590 | 1.1459 | 1.3831 |
| Buri Ram | 0-21 | 14.5 | 10th | 7.6 | 1.1247 | 1.0439 | 1.2118 |
| Buri Ram | 0-21 | 14.5 | 90th | 14.5 | 1.0000 | 1.0000 | 1.0000 |
| Buri Ram | 0-21 | 14.5 | 99th | 17.6 | 1.0243 | 1.0055 | 1.0435 |
| Chachoengsao | 0-7 | 10.8 | 1st | 4.5 | 1.1260 | 1.0668 | 1.1885 |
| Chachoengsao | 0-7 | 10.8 | 10th | 7.2 | 1.0365 | 1.0090 | 1.0647 |
| Chachoengsao | 0-7 | 10.8 | 90th | 14.0 | 1.0173 | 0.9914 | 1.0440 |
| Chachoengsao | 0-7 | 10.8 | 99th | 17.3 | 1.0610 | 1.0230 | 1.1004 |
| Chachoengsao | 0-14 | 11.2 | 1st | 4.5 | 1.1693 | 1.0958 | 1.2476 |
| Chachoengsao | 0-14 | 11.2 | 10th | 7.2 | 1.0495 | 1.0093 | 1.0913 |
| Chachoengsao | 0-14 | 11.2 | 90th | 14.0 | 1.0119 | 0.9843 | 1.0402 |
| Chachoengsao | 0-14 | 11.2 | 99th | 17.3 | 1.0407 | 1.0053 | 1.0773 |
| Chachoengsao | 0-21 | 12.0 | 1st | 4.5 | 1.2254 | 1.1200 | 1.3408 |
| Chachoengsao | 0-21 | 12.0 | 10th | 7.2 | 1.0699 | 1.0055 | 1.1385 |
| Chachoengsao | 0-21 | 12.0 | 90th | 14.0 | 1.0030 | 0.9782 | 1.0285 |
| Chachoengsao | 0-21 | 12.0 | 99th | 17.3 | 1.0113 | 0.9795 | 1.0441 |
| Chai Nat | 0-7 | 9.6 | 1st | 4.0 | 1.0943 | 1.0241 | 1.1694 |
| Chai Nat | 0-7 | 9.6 | 10th | 6.9 | 1.0204 | 0.9982 | 1.0431 |
| Chai Nat | 0-7 | 9.6 | 90th | 12.9 | 1.0198 | 0.9879 | 1.0528 |
| Chai Nat | 0-7 | 9.6 | 99th | 15.6 | 1.0540 | 1.0070 | 1.1032 |
| Chai Nat | 0-14 | 10.2 | 1st | 4.0 | 1.1286 | 1.0418 | 1.2227 |
| Chai Nat | 0-14 | 10.2 | 10th | 6.9 | 1.0321 | 0.9982 | 1.0671 |
| Chai Nat | 0-14 | 10.2 | 90th | 12.9 | 1.0128 | 0.9783 | 1.0484 |
| Chai Nat | 0-14 | 10.2 | 99th | 15.6 | 1.0387 | 0.9895 | 1.0903 |
| Chai Nat | 0-21 | 11.7 | 1st | 4.0 | 1.1617 | 1.0383 | 1.2996 |
| Chai Nat | 0-21 | 11.7 | 10th | 6.9 | 1.0526 | 0.9869 | 1.1225 |
| Chai Nat | 0-21 | 11.7 | 90th | 12.9 | 1.0015 | 0.9820 | 1.0215 |
| Chai Nat | 0-21 | 11.7 | 99th | 15.6 | 1.0125 | 0.9735 | 1.0530 |
| Chaiyaphum | 0-7 | 9.9 | 1st | 3.5 | 1.1474 | 1.0746 | 1.2251 |
| Chaiyaphum | 0-7 | 9.9 | 10th | 6.6 | 1.0350 | 1.0125 | 1.0580 |
| Chaiyaphum | 0-7 | 9.9 | 90th | 13.4 | 1.0222 | 0.9953 | 1.0499 |
| Chaiyaphum | 0-7 | 9.9 | 99th | 16.5 | 1.0572 | 1.0185 | 1.0973 |
| Chaiyaphum | 0-14 | 10.3 | 1st | 3.5 | 1.2105 | 1.1182 | 1.3104 |
| Chaiyaphum | 0-14 | 10.3 | 10th | 6.6 | 1.0523 | 1.0211 | 1.0845 |
| Chaiyaphum | 0-14 | 10.3 | 90th | 13.4 | 1.0149 | 0.9846 | 1.0461 |
| Chaiyaphum | 0-14 | 10.3 | 99th | 16.5 | 1.0320 | 0.9924 | 1.0732 |
| Chaiyaphum | 0-21 | 26.0 | 1st | 3.5 | 1.3815 | 1.0990 | 1.7366 |
| Chaiyaphum | 0-21 | 26.0 | 10th | 6.6 | 1.1516 | 0.9925 | 1.3362 |
| Chaiyaphum | 0-21 | 26.0 | 90th | 13.4 | 1.0707 | 0.9017 | 1.2713 |
| Chaiyaphum | 0-21 | 26.0 | 99th | 16.5 | 1.0684 | 0.9019 | 1.2656 |
| Chanthaburi | 0-7 | 9.7 | 1st | 3.4 | 1.1145 | 1.0339 | 1.2013 |
| Chanthaburi | 0-7 | 9.7 | 10th | 5.6 | 1.0479 | 1.0123 | 1.0847 |
| Chanthaburi | 0-7 | 9.7 | 90th | 11.5 | 1.0060 | 0.9887 | 1.0236 |
| Chanthaburi | 0-7 | 9.7 | 99th | 14.3 | 1.0324 | 0.9933 | 1.0730 |
| Chanthaburi | 0-14 | 10.4 | 1st | 3.4 | 1.1648 | 1.0646 | 1.2744 |
| Chanthaburi | 0-14 | 10.4 | 10th | 5.6 | 1.0730 | 1.0266 | 1.1215 |
| Chanthaburi | 0-14 | 10.4 | 90th | 11.5 | 1.0022 | 0.9875 | 1.0172 |
| Chanthaburi | 0-14 | 10.4 | 99th | 14.3 | 1.0205 | 0.9791 | 1.0635 |
| Chanthaburi | 0-21 | 11.7 | 1st | 3.4 | 1.2111 | 1.0681 | 1.3732 |
| Chanthaburi | 0-21 | 11.7 | 10th | 5.6 | 1.1011 | 1.0279 | 1.1796 |
| Chanthaburi | 0-21 | 11.7 | 90th | 11.5 | 1.0001 | 0.9964 | 1.0038 |
| Chanthaburi | 0-21 | 11.7 | 99th | 14.3 | 1.0046 | 0.9704 | 1.0399 |
| Chiang Mai | 0-7 | 8.8 | 1st | 2.7 | 1.0931 | 1.0259 | 1.1647 |
| Chiang Mai | 0-7 | 8.8 | 10th | 5.3 | 1.0309 | 1.0061 | 1.0563 |
| Chiang Mai | 0-7 | 8.8 | 90th | 12.6 | 1.0223 | 0.9959 | 1.0495 |
| Chiang Mai | 0-7 | 8.8 | 99th | 14.8 | 1.0436 | 1.0069 | 1.0816 |
| Chiang Mai | 0-14 | 11.1 | 1st | 2.7 | 1.1299 | 1.0458 | 1.2208 |
| Chiang Mai | 0-14 | 11.1 | 10th | 5.3 | 1.0598 | 1.0203 | 1.1009 |
| Chiang Mai | 0-14 | 11.1 | 90th | 12.6 | 1.0031 | 0.9897 | 1.0167 |
| Chiang Mai | 0-14 | 11.1 | 99th | 14.8 | 1.0167 | 0.9922 | 1.0418 |
| Chiang Mai | 0-21 | 14.2 | 1st | 2.7 | 1.1594 | 1.0415 | 1.2907 |
| Chiang Mai | 0-21 | 14.2 | 10th | 5.3 | 1.1211 | 1.0477 | 1.1996 |
| Chiang Mai | 0-21 | 14.2 | 90th | 12.6 | 1.0064 | 0.9935 | 1.0195 |
| Chiang Mai | 0-21 | 14.2 | 99th | 14.8 | 1.0007 | 0.9980 | 1.0034 |
| Chiang Rai | 0-7 | 9.0 | 1st | 2.8 | 1.1835 | 1.1098 | 1.2621 |
| Chiang Rai | 0-7 | 9.0 | 10th | 6.4 | 1.0296 | 1.0137 | 1.0458 |
| Chiang Rai | 0-7 | 9.0 | 90th | 17.4 | 1.0721 | 1.0326 | 1.1132 |
| Chiang Rai | 0-7 | 9.0 | 99th | 20.6 | 1.0849 | 1.0388 | 1.1330 |
| Chiang Rai | 0-14 | 9.0 | 1st | 2.8 | 1.2603 | 1.1667 | 1.3615 |
| Chiang Rai | 0-14 | 9.0 | 10th | 6.4 | 1.0402 | 1.0207 | 1.0601 |
| Chiang Rai | 0-14 | 9.0 | 90th | 17.4 | 1.0720 | 1.0267 | 1.1192 |
| Chiang Rai | 0-14 | 9.0 | 99th | 20.6 | 1.0595 | 1.0125 | 1.1087 |
| Chiang Rai | 0-21 | 26.0 | 1st | 2.8 | 1.4078 | 1.1674 | 1.6977 |
| Chiang Rai | 0-21 | 26.0 | 10th | 6.4 | 1.1061 | 0.9881 | 1.2381 |
| Chiang Rai | 0-21 | 26.0 | 90th | 17.4 | 1.1286 | 0.9936 | 1.2818 |
| Chiang Rai | 0-21 | 26.0 | 99th | 20.6 | 1.0963 | 1.0000 | 1.2019 |
| Chon Buri | 0-7 | 8.7 | 1st | 3.8 | 1.0503 | 0.9955 | 1.1081 |
| Chon Buri | 0-7 | 8.7 | 10th | 5.3 | 1.0236 | 0.9960 | 1.0520 |
| Chon Buri | 0-7 | 8.7 | 90th | 9.5 | 1.0014 | 0.9964 | 1.0064 |
| Chon Buri | 0-7 | 8.7 | 99th | 11.5 | 1.0128 | 0.9945 | 1.0315 |
| Chon Buri | 0-14 | 9.5 | 1st | 3.8 | 1.0616 | 0.9948 | 1.1330 |
| Chon Buri | 0-14 | 9.5 | 10th | 5.3 | 1.0323 | 0.9979 | 1.0680 |
| Chon Buri | 0-14 | 9.5 | 90th | 9.5 | 1.0000 | 0.9997 | 1.0004 |
| Chon Buri | 0-14 | 9.5 | 99th | 11.5 | 1.0063 | 0.9870 | 1.0259 |
| Chon Buri | 0-21 | 11.8 | 1st | 3.8 | 1.0473 | 0.9594 | 1.1432 |
| Chon Buri | 0-21 | 11.8 | 10th | 5.3 | 1.0346 | 0.9795 | 1.0928 |
| Chon Buri | 0-21 | 11.8 | 90th | 9.5 | 1.0061 | 0.9760 | 1.0372 |
| Chon Buri | 0-21 | 11.8 | 99th | 11.5 | 1.0002 | 0.9955 | 1.0049 |
| Chumphon | 0-7 | 12.3 | 1st | 2.7 | 1.2012 | 1.1007 | 1.3109 |
| Chumphon | 0-7 | 12.3 | 10th | 5.6 | 1.0941 | 1.0453 | 1.1451 |
| Chumphon | 0-7 | 12.3 | 90th | 10.8 | 1.0041 | 0.9898 | 1.0186 |
| Chumphon | 0-7 | 12.3 | 99th | 13.2 | 1.0012 | 0.9941 | 1.0083 |
| Chumphon | 0-14 | 12.9 | 1st | 2.7 | 1.3086 | 1.1788 | 1.4526 |
| Chumphon | 0-14 | 12.9 | 10th | 5.6 | 1.1375 | 1.0696 | 1.2096 |
| Chumphon | 0-14 | 12.9 | 90th | 10.8 | 1.0071 | 0.9815 | 1.0334 |
| Chumphon | 0-14 | 12.9 | 99th | 13.2 | 1.0001 | 0.9974 | 1.0029 |
| Chumphon | 0-21 | 16.4 | 1st | 2.7 | 1.4334 | 1.2294 | 1.6712 |
| Chumphon | 0-21 | 16.4 | 10th | 5.6 | 1.1812 | 1.0737 | 1.2995 |
| Chumphon | 0-21 | 16.4 | 90th | 10.8 | 1.0108 | 0.9584 | 1.0662 |
| Chumphon | 0-21 | 16.4 | 99th | 13.2 | 1.0018 | 0.9835 | 1.0204 |
| Kalasin | 0-7 | 9.6 | 1st | 2.6 | 1.1859 | 1.0948 | 1.2846 |
| Kalasin | 0-7 | 9.6 | 10th | 6.1 | 1.0441 | 1.0194 | 1.0694 |
| Kalasin | 0-7 | 9.6 | 90th | 14.0 | 1.0359 | 1.0032 | 1.0698 |
| Kalasin | 0-7 | 9.6 | 99th | 16.4 | 1.0717 | 1.0307 | 1.1144 |
| Kalasin | 0-14 | 9.5 | 1st | 2.6 | 1.2427 | 1.1277 | 1.3693 |
| Kalasin | 0-14 | 9.5 | 10th | 6.1 | 1.0537 | 1.0245 | 1.0839 |
| Kalasin | 0-14 | 9.5 | 90th | 14.0 | 1.0358 | 0.9931 | 1.0805 |
| Kalasin | 0-14 | 9.5 | 99th | 16.4 | 1.0572 | 1.0089 | 1.1078 |
| Kalasin | 0-21 | 26.0 | 1st | 2.6 | 1.3541 | 1.0555 | 1.7372 |
| Kalasin | 0-21 | 26.0 | 10th | 6.1 | 1.0981 | 0.9467 | 1.2737 |
| Kalasin | 0-21 | 26.0 | 90th | 14.0 | 1.0484 | 0.8831 | 1.2446 |
| Kalasin | 0-21 | 26.0 | 99th | 16.4 | 1.0558 | 0.8922 | 1.2494 |
| Kamphaeng Phet | 0-7 | 10.0 | 1st | 3.2 | 1.0628 | 0.9871 | 1.1443 |
| Kamphaeng Phet | 0-7 | 10.0 | 10th | 6.6 | 1.0158 | 0.9914 | 1.0408 |
| Kamphaeng Phet | 0-7 | 10.0 | 90th | 14.2 | 1.0240 | 0.9900 | 1.0593 |
| Kamphaeng Phet | 0-7 | 10.0 | 99th | 17.3 | 1.0702 | 1.0266 | 1.1158 |
| Kamphaeng Phet | 0-14 | 12.5 | 1st | 3.2 | 1.1051 | 1.0116 | 1.2072 |
| Kamphaeng Phet | 0-14 | 12.5 | 10th | 6.6 | 1.0506 | 0.9947 | 1.1097 |
| Kamphaeng Phet | 0-14 | 12.5 | 90th | 14.2 | 1.0065 | 0.9932 | 1.0200 |
| Kamphaeng Phet | 0-14 | 12.5 | 99th | 17.3 | 1.0489 | 1.0237 | 1.0748 |
| Kamphaeng Phet | 0-21 | 14.4 | 1st | 3.2 | 1.1345 | 1.0022 | 1.2842 |
| Kamphaeng Phet | 0-21 | 14.4 | 10th | 6.6 | 1.1110 | 1.0187 | 1.2117 |
| Kamphaeng Phet | 0-21 | 14.4 | 90th | 14.2 | 1.0001 | 0.9988 | 1.0015 |
| Kamphaeng Phet | 0-21 | 14.4 | 99th | 17.3 | 1.0221 | 1.0049 | 1.0395 |
| Kanchanaburi | 0-7 | 9.1 | 1st | 4.7 | 1.0696 | 1.0291 | 1.1117 |
| Kanchanaburi | 0-7 | 9.1 | 10th | 7.4 | 1.0093 | 0.9973 | 1.0214 |
| Kanchanaburi | 0-7 | 9.1 | 90th | 15.5 | 1.0571 | 1.0106 | 1.1057 |
| Kanchanaburi | 0-7 | 9.1 | 99th | 18.0 | 1.0845 | 1.0337 | 1.1377 |
| Kanchanaburi | 0-14 | 9.4 | 1st | 4.7 | 1.1010 | 1.0503 | 1.1541 |
| Kanchanaburi | 0-14 | 9.4 | 10th | 7.4 | 1.0156 | 0.9975 | 1.0340 |
| Kanchanaburi | 0-14 | 9.4 | 90th | 15.5 | 1.0449 | 0.9914 | 1.1013 |
| Kanchanaburi | 0-14 | 9.4 | 99th | 18.0 | 1.0559 | 1.0050 | 1.1094 |
| Kanchanaburi | 0-21 | 26.0 | 1st | 4.7 | 1.1571 | 0.9511 | 1.4078 |
| Kanchanaburi | 0-21 | 26.0 | 10th | 7.4 | 1.0423 | 0.8976 | 1.2103 |
| Kanchanaburi | 0-21 | 26.0 | 90th | 15.5 | 1.0270 | 0.8432 | 1.2508 |
| Kanchanaburi | 0-21 | 26.0 | 99th | 18.0 | 1.0278 | 0.8608 | 1.2273 |
| Khon Kaen | 0-7 | 9.8 | 1st | 3.5 | 1.1167 | 1.0551 | 1.1819 |
| Khon Kaen | 0-7 | 9.8 | 10th | 7.0 | 1.0207 | 1.0050 | 1.0366 |
| Khon Kaen | 0-7 | 9.8 | 90th | 14.3 | 1.0414 | 1.0119 | 1.0718 |
| Khon Kaen | 0-7 | 9.8 | 99th | 17.1 | 1.0991 | 1.0617 | 1.1378 |
| Khon Kaen | 0-14 | 11.6 | 1st | 3.5 | 1.1455 | 1.0711 | 1.2250 |
| Khon Kaen | 0-14 | 11.6 | 10th | 7.0 | 1.0442 | 1.0087 | 1.0811 |
| Khon Kaen | 0-14 | 11.6 | 90th | 14.3 | 1.0152 | 0.9957 | 1.0350 |
| Khon Kaen | 0-14 | 11.6 | 99th | 17.1 | 1.0602 | 1.0339 | 1.0873 |
| Khon Kaen | 0-21 | 14.3 | 1st | 3.5 | 1.1986 | 1.0901 | 1.3180 |
| Khon Kaen | 0-21 | 14.3 | 10th | 7.0 | 1.1156 | 1.0418 | 1.1948 |
| Khon Kaen | 0-21 | 14.3 | 90th | 14.3 | 1.0000 | 1.0000 | 1.0000 |
| Khon Kaen | 0-21 | 14.3 | 99th | 17.1 | 1.0190 | 1.0042 | 1.0341 |
| Krabi | 0-7 | 9.2 | 1st | 2.9 | 1.1444 | 1.0572 | 1.2389 |
| Krabi | 0-7 | 9.2 | 10th | 5.2 | 1.0593 | 1.0196 | 1.1005 |
| Krabi | 0-7 | 9.2 | 90th | 10.9 | 1.0074 | 0.9885 | 1.0266 |
| Krabi | 0-7 | 9.2 | 99th | 13.3 | 1.0343 | 0.9905 | 1.0800 |
| Krabi | 0-14 | 10.3 | 1st | 2.9 | 1.1834 | 1.0710 | 1.3075 |
| Krabi | 0-14 | 10.3 | 10th | 5.2 | 1.0852 | 1.0269 | 1.1469 |
| Krabi | 0-14 | 10.3 | 90th | 10.9 | 1.0007 | 0.9913 | 1.0103 |
| Krabi | 0-14 | 10.3 | 99th | 13.3 | 1.0154 | 0.9741 | 1.0583 |
| Krabi | 0-21 | 13.5 | 1st | 2.9 | 1.2171 | 1.0495 | 1.4115 |
| Krabi | 0-21 | 13.5 | 10th | 5.2 | 1.1304 | 1.0115 | 1.2633 |
| Krabi | 0-21 | 13.5 | 90th | 10.9 | 1.0108 | 0.9623 | 1.0617 |
| Krabi | 0-21 | 13.5 | 99th | 13.3 | 1.0001 | 0.9966 | 1.0035 |
| Lampang | 0-7 | 8.6 | 1st | 4.0 | 1.1180 | 1.0623 | 1.1766 |
| Lampang | 0-7 | 8.6 | 10th | 7.4 | 1.0071 | 0.9990 | 1.0152 |
| Lampang | 0-7 | 8.6 | 90th | 17.6 | 1.1516 | 1.0989 | 1.2068 |
| Lampang | 0-7 | 8.6 | 99th | 21.2 | 1.2181 | 1.1530 | 1.2869 |
| Lampang | 0-14 | 9.0 | 1st | 4.0 | 1.1337 | 1.0649 | 1.2069 |
| Lampang | 0-14 | 9.0 | 10th | 7.4 | 1.0120 | 0.9989 | 1.0252 |
| Lampang | 0-14 | 9.0 | 90th | 17.6 | 1.1203 | 1.0664 | 1.1769 |
| Lampang | 0-14 | 9.0 | 99th | 21.2 | 1.1786 | 1.1128 | 1.2483 |
| Lampang | 0-21 | 10.6 | 1st | 4.0 | 1.1306 | 1.0342 | 1.2360 |
| Lampang | 0-21 | 10.6 | 10th | 7.4 | 1.0260 | 0.9911 | 1.0621 |
| Lampang | 0-21 | 10.6 | 90th | 17.6 | 1.0513 | 1.0060 | 1.0986 |
| Lampang | 0-21 | 10.6 | 99th | 21.2 | 1.0993 | 1.0418 | 1.1600 |
| Lamphun | 0-7 | 8.3 | 1st | 3.4 | 1.1008 | 1.0284 | 1.1783 |
| Lamphun | 0-7 | 8.3 | 10th | 7.0 | 1.0070 | 0.9965 | 1.0175 |
| Lamphun | 0-7 | 8.3 | 90th | 17.8 | 1.1190 | 1.0591 | 1.1822 |
| Lamphun | 0-7 | 8.3 | 99th | 21.4 | 1.1594 | 1.0865 | 1.2370 |
| Lamphun | 0-14 | 9.0 | 1st | 3.4 | 1.1212 | 1.0294 | 1.2211 |
| Lamphun | 0-14 | 9.0 | 10th | 7.0 | 1.0144 | 0.9957 | 1.0334 |
| Lamphun | 0-14 | 9.0 | 90th | 17.8 | 1.0936 | 1.0342 | 1.1565 |
| Lamphun | 0-14 | 9.0 | 99th | 21.4 | 1.1416 | 1.0670 | 1.2215 |
| Lamphun | 0-21 | 11.3 | 1st | 3.4 | 1.1074 | 0.9820 | 1.2488 |
| Lamphun | 0-21 | 11.3 | 10th | 7.0 | 1.0287 | 0.9749 | 1.0855 |
| Lamphun | 0-21 | 11.3 | 90th | 17.8 | 1.0405 | 0.9990 | 1.0837 |
| Lamphun | 0-21 | 11.3 | 99th | 21.4 | 1.0890 | 1.0190 | 1.1639 |
| Loei | 0-7 | 9.8 | 1st | 2.8 | 1.0966 | 1.0133 | 1.1868 |
| Loei | 0-7 | 9.8 | 10th | 6.8 | 1.0173 | 0.9966 | 1.0385 |
| Loei | 0-7 | 9.8 | 90th | 16.9 | 1.0674 | 1.0236 | 1.1129 |
| Loei | 0-7 | 9.8 | 99th | 20.6 | 1.1462 | 1.0889 | 1.2065 |
| Loei | 0-14 | 10.9 | 1st | 2.8 | 1.1323 | 1.0300 | 1.2447 |
| Loei | 0-14 | 10.9 | 10th | 6.8 | 1.0319 | 0.9941 | 1.0712 |
| Loei | 0-14 | 10.9 | 90th | 16.9 | 1.0478 | 1.0103 | 1.0866 |
| Loei | 0-14 | 10.9 | 99th | 20.6 | 1.1180 | 1.0595 | 1.1797 |
| Loei | 0-21 | 12.9 | 1st | 2.8 | 1.1456 | 1.0061 | 1.3044 |
| Loei | 0-21 | 12.9 | 10th | 6.8 | 1.0514 | 0.9757 | 1.1330 |
| Loei | 0-21 | 12.9 | 90th | 16.9 | 1.0181 | 0.9965 | 1.0401 |
| Loei | 0-21 | 12.9 | 99th | 20.6 | 1.0632 | 1.0028 | 1.1273 |
| Lop Buri | 0-7 | 11.7 | 1st | 4.1 | 1.1078 | 1.0439 | 1.1756 |
| Lop Buri | 0-7 | 11.7 | 10th | 7.5 | 1.0342 | 1.0015 | 1.0681 |
| Lop Buri | 0-7 | 11.7 | 90th | 13.5 | 1.0057 | 0.9920 | 1.0196 |
| Lop Buri | 0-7 | 11.7 | 99th | 16.7 | 1.0458 | 1.0190 | 1.0733 |
| Lop Buri | 0-14 | 13.2 | 1st | 4.1 | 1.1804 | 1.0954 | 1.2721 |
| Lop Buri | 0-14 | 13.2 | 10th | 7.5 | 1.0740 | 1.0138 | 1.1378 |
| Lop Buri | 0-14 | 13.2 | 90th | 13.5 | 1.0002 | 0.9982 | 1.0021 |
| Lop Buri | 0-14 | 13.2 | 99th | 16.7 | 1.0266 | 1.0088 | 1.0448 |
| Lop Buri | 0-21 | 15.0 | 1st | 4.1 | 1.2733 | 1.1453 | 1.4157 |
| Lop Buri | 0-21 | 15.0 | 10th | 7.5 | 1.1407 | 1.0446 | 1.2457 |
| Lop Buri | 0-21 | 15.0 | 90th | 13.5 | 1.0061 | 0.9952 | 1.0171 |
| Lop Buri | 0-21 | 15.0 | 99th | 16.7 | 1.0058 | 0.9954 | 1.0164 |
| Mae Hong Son | 0-7 | 9.5 | 1st | 4.9 | 1.0280 | 0.9773 | 1.0813 |
| Mae Hong Son | 0-7 | 9.5 | 10th | 7.6 | 1.0051 | 0.9871 | 1.0234 |
| Mae Hong Son | 0-7 | 9.5 | 90th | 19.2 | 1.1221 | 1.0516 | 1.1973 |
| Mae Hong Son | 0-7 | 9.5 | 99th | 22.7 | 1.2162 | 1.1109 | 1.3315 |
| Mae Hong Son | 0-14 | 12.3 | 1st | 4.9 | 1.0589 | 0.9801 | 1.1440 |
| Mae Hong Son | 0-14 | 12.3 | 10th | 7.6 | 1.0311 | 0.9710 | 1.0950 |
| Mae Hong Son | 0-14 | 12.3 | 90th | 19.2 | 1.1024 | 1.0479 | 1.1598 |
| Mae Hong Son | 0-14 | 12.3 | 99th | 22.7 | 1.2198 | 1.0876 | 1.3682 |
| Mae Hong Son | 0-21 | 14.3 | 1st | 4.9 | 1.1148 | 0.9954 | 1.2485 |
| Mae Hong Son | 0-21 | 14.3 | 10th | 7.6 | 1.1018 | 0.9932 | 1.2223 |
| Mae Hong Son | 0-21 | 14.3 | 90th | 19.2 | 1.0708 | 1.0189 | 1.1253 |
| Mae Hong Son | 0-21 | 14.3 | 99th | 22.7 | 1.1941 | 1.0440 | 1.3657 |
| Maha Sarakham | 0-7 | 9.6 | 1st | 3.8 | 1.0859 | 1.0204 | 1.1555 |
| Maha Sarakham | 0-7 | 9.6 | 10th | 7.4 | 1.0114 | 0.9960 | 1.0271 |
| Maha Sarakham | 0-7 | 9.6 | 90th | 14.5 | 1.0365 | 0.9991 | 1.0753 |
| Maha Sarakham | 0-7 | 9.6 | 99th | 17.7 | 1.0858 | 1.0399 | 1.1338 |
| Maha Sarakham | 0-14 | 10.6 | 1st | 3.8 | 1.1243 | 1.0430 | 1.2121 |
| Maha Sarakham | 0-14 | 10.6 | 10th | 7.4 | 1.0235 | 0.9932 | 1.0547 |
| Maha Sarakham | 0-14 | 10.6 | 90th | 14.5 | 1.0189 | 0.9829 | 1.0563 |
| Maha Sarakham | 0-14 | 10.6 | 99th | 17.7 | 1.0507 | 1.0105 | 1.0925 |
| Maha Sarakham | 0-21 | 13.9 | 1st | 3.8 | 1.1797 | 1.0600 | 1.3130 |
| Maha Sarakham | 0-21 | 13.9 | 10th | 7.4 | 1.0572 | 0.9744 | 1.1469 |
| Maha Sarakham | 0-21 | 13.9 | 90th | 14.5 | 1.0003 | 0.9961 | 1.0045 |
| Maha Sarakham | 0-21 | 13.9 | 99th | 17.7 | 1.0097 | 0.9871 | 1.0328 |
| Mukdahan | 0-7 | 9.7 | 1st | 3.0 | 1.1493 | 1.0497 | 1.2582 |
| Mukdahan | 0-7 | 9.7 | 10th | 6.5 | 1.0313 | 1.0024 | 1.0611 |
| Mukdahan | 0-7 | 9.7 | 90th | 13.9 | 1.0336 | 0.9913 | 1.0778 |
| Mukdahan | 0-7 | 9.7 | 99th | 16.6 | 1.0746 | 1.0201 | 1.1321 |
| Mukdahan | 0-14 | 10.9 | 1st | 3.0 | 1.1978 | 1.0715 | 1.3389 |
| Mukdahan | 0-14 | 10.9 | 10th | 6.5 | 1.0549 | 1.0048 | 1.1076 |
| Mukdahan | 0-14 | 10.9 | 90th | 13.9 | 1.0143 | 0.9778 | 1.0522 |
| Mukdahan | 0-14 | 10.9 | 99th | 16.6 | 1.0449 | 1.0006 | 1.0911 |
| Mukdahan | 0-21 | 13.9 | 1st | 3.0 | 1.2494 | 1.0681 | 1.4614 |
| Mukdahan | 0-21 | 13.9 | 10th | 6.5 | 1.1041 | 0.9932 | 1.2273 |
| Mukdahan | 0-21 | 13.9 | 90th | 13.9 | 1.0000 | 1.0000 | 1.0000 |
| Mukdahan | 0-21 | 13.9 | 99th | 16.6 | 1.0097 | 0.9930 | 1.0266 |
| Nakhon Nayok | 0-7 | 9.8 | 1st | 1.7 | 1.1665 | 1.0214 | 1.3321 |
| Nakhon Nayok | 0-7 | 9.8 | 10th | 3.7 | 1.0954 | 1.0129 | 1.1846 |
| Nakhon Nayok | 0-7 | 9.8 | 90th | 9.6 | 1.0001 | 0.9977 | 1.0024 |
| Nakhon Nayok | 0-7 | 9.8 | 99th | 11.9 | 1.0078 | 0.9822 | 1.0341 |
| Nakhon Nayok | 0-14 | 11.2 | 1st | 1.7 | 1.2279 | 1.0418 | 1.4472 |
| Nakhon Nayok | 0-14 | 11.2 | 10th | 3.7 | 1.1394 | 1.0319 | 1.2580 |
| Nakhon Nayok | 0-14 | 11.2 | 90th | 9.6 | 1.0050 | 0.9784 | 1.0323 |
| Nakhon Nayok | 0-14 | 11.2 | 99th | 11.9 | 1.0007 | 0.9896 | 1.0119 |
| Nakhon Nayok | 0-21 | 14.1 | 1st | 1.7 | 1.2804 | 1.0140 | 1.6166 |
| Nakhon Nayok | 0-21 | 14.1 | 10th | 3.7 | 1.1944 | 1.0317 | 1.3827 |
| Nakhon Nayok | 0-21 | 14.1 | 90th | 9.6 | 1.0329 | 0.9430 | 1.1314 |
| Nakhon Nayok | 0-21 | 14.1 | 99th | 11.9 | 1.0077 | 0.9699 | 1.0469 |
| Nakhon Pathom | 0-7 | 11.2 | 1st | 4.0 | 1.0892 | 1.0312 | 1.1505 |
| Nakhon Pathom | 0-7 | 11.2 | 10th | 7.3 | 1.0244 | 0.9968 | 1.0527 |
| Nakhon Pathom | 0-7 | 11.2 | 90th | 13.3 | 1.0061 | 0.9894 | 1.0232 |
| Nakhon Pathom | 0-7 | 11.2 | 99th | 16.5 | 1.0357 | 1.0063 | 1.0660 |
| Nakhon Pathom | 0-14 | 12.5 | 1st | 4.0 | 1.1463 | 1.0706 | 1.2273 |
| Nakhon Pathom | 0-14 | 12.5 | 10th | 7.3 | 1.0499 | 0.9986 | 1.1039 |
| Nakhon Pathom | 0-14 | 12.5 | 90th | 13.3 | 1.0011 | 0.9939 | 1.0083 |
| Nakhon Pathom | 0-14 | 12.5 | 99th | 16.5 | 1.0235 | 1.0026 | 1.0447 |
| Nakhon Pathom | 0-21 | 14.3 | 1st | 4.0 | 1.2060 | 1.0927 | 1.3311 |
| Nakhon Pathom | 0-21 | 14.3 | 10th | 7.3 | 1.0891 | 1.0024 | 1.1834 |
| Nakhon Pathom | 0-21 | 14.3 | 90th | 13.3 | 1.0017 | 0.9933 | 1.0101 |
| Nakhon Pathom | 0-21 | 14.3 | 99th | 16.5 | 1.0069 | 0.9949 | 1.0191 |
| Nakhon Phanom | 0-7 | 10.1 | 1st | 2.2 | 1.1613 | 1.0652 | 1.2660 |
| Nakhon Phanom | 0-7 | 10.1 | 10th | 5.8 | 1.0477 | 1.0167 | 1.0797 |
| Nakhon Phanom | 0-7 | 10.1 | 90th | 14.7 | 1.0371 | 1.0029 | 1.0725 |
| Nakhon Phanom | 0-7 | 10.1 | 99th | 17.6 | 1.0918 | 1.0499 | 1.1353 |
| Nakhon Phanom | 0-14 | 11.7 | 1st | 2.2 | 1.1976 | 1.0814 | 1.3263 |
| Nakhon Phanom | 0-14 | 11.7 | 10th | 5.8 | 1.0757 | 1.0236 | 1.1305 |
| Nakhon Phanom | 0-14 | 11.7 | 90th | 14.7 | 1.0151 | 0.9916 | 1.0392 |
| Nakhon Phanom | 0-14 | 11.7 | 99th | 17.6 | 1.0579 | 1.0280 | 1.0887 |
| Nakhon Phanom | 0-21 | 14.4 | 1st | 2.2 | 1.2432 | 1.0790 | 1.4325 |
| Nakhon Phanom | 0-21 | 14.4 | 10th | 5.8 | 1.1371 | 1.0411 | 1.2418 |
| Nakhon Phanom | 0-21 | 14.4 | 90th | 14.7 | 1.0001 | 0.9986 | 1.0016 |
| Nakhon Phanom | 0-21 | 14.4 | 99th | 17.6 | 1.0181 | 0.9993 | 1.0373 |
| Nakhon Ratchasima | 0-7 | 9.8 | 1st | 3.6 | 1.1354 | 1.0823 | 1.1910 |
| Nakhon Ratchasima | 0-7 | 9.8 | 10th | 6.9 | 1.0270 | 1.0121 | 1.0422 |
| Nakhon Ratchasima | 0-7 | 9.8 | 90th | 13.3 | 1.0277 | 1.0047 | 1.0513 |
| Nakhon Ratchasima | 0-7 | 9.8 | 99th | 16.4 | 1.0867 | 1.0541 | 1.1204 |
| Nakhon Ratchasima | 0-14 | 11.5 | 1st | 3.6 | 1.1779 | 1.1123 | 1.2475 |
| Nakhon Ratchasima | 0-14 | 11.5 | 10th | 6.9 | 1.0540 | 1.0211 | 1.0881 |
| Nakhon Ratchasima | 0-14 | 11.5 | 90th | 13.3 | 1.0071 | 0.9929 | 1.0215 |
| Nakhon Ratchasima | 0-14 | 11.5 | 99th | 16.4 | 1.0480 | 1.0247 | 1.0717 |
| Nakhon Ratchasima | 0-21 | 14.4 | 1st | 3.6 | 1.2550 | 1.1553 | 1.3633 |
| Nakhon Ratchasima | 0-21 | 14.4 | 10th | 6.9 | 1.1340 | 1.0629 | 1.2098 |
| Nakhon Ratchasima | 0-21 | 14.4 | 90th | 13.3 | 1.0031 | 0.9961 | 1.0101 |
| Nakhon Ratchasima | 0-21 | 14.4 | 99th | 16.4 | 1.0102 | 1.0010 | 1.0196 |
| Nakhon Sawan | 0-7 | 8.8 | 1st | 4.2 | 1.1093 | 1.0542 | 1.1674 |
| Nakhon Sawan | 0-7 | 8.8 | 10th | 7.3 | 1.0105 | 1.0000 | 1.0211 |
| Nakhon Sawan | 0-7 | 8.8 | 90th | 13.4 | 1.0554 | 1.0187 | 1.0935 |
| Nakhon Sawan | 0-7 | 8.8 | 99th | 16.7 | 1.1142 | 1.0656 | 1.1651 |
| Nakhon Sawan | 0-14 | 9.3 | 1st | 4.2 | 1.1313 | 1.0626 | 1.2043 |
| Nakhon Sawan | 0-14 | 9.3 | 10th | 7.3 | 1.0175 | 1.0005 | 1.0348 |
| Nakhon Sawan | 0-14 | 9.3 | 90th | 13.4 | 1.0373 | 0.9943 | 1.0822 |
| Nakhon Sawan | 0-14 | 9.3 | 99th | 16.7 | 1.0785 | 1.0263 | 1.1333 |
| Nakhon Sawan | 0-21 | 11.6 | 1st | 4.2 | 1.1650 | 1.0656 | 1.2736 |
| Nakhon Sawan | 0-21 | 11.6 | 10th | 7.3 | 1.0440 | 0.9923 | 1.0983 |
| Nakhon Sawan | 0-21 | 11.6 | 90th | 13.4 | 1.0030 | 0.9801 | 1.0263 |
| Nakhon Sawan | 0-21 | 11.6 | 99th | 16.7 | 1.0156 | 0.9810 | 1.0515 |
| Nakhon Si Thammarat | 0-7 | 10.3 | 1st | 3.3 | 1.2132 | 1.1465 | 1.2837 |
| Nakhon Si Thammarat | 0-7 | 10.3 | 10th | 6.6 | 1.0491 | 1.0249 | 1.0739 |
| Nakhon Si Thammarat | 0-7 | 10.3 | 90th | 12.2 | 1.0078 | 0.9913 | 1.0245 |
| Nakhon Si Thammarat | 0-7 | 10.3 | 99th | 19.2 | 1.0688 | 1.0196 | 1.1205 |
| Nakhon Si Thammarat | 0-14 | 10.3 | 1st | 3.3 | 1.3061 | 1.2199 | 1.3984 |
| Nakhon Si Thammarat | 0-14 | 10.3 | 10th | 6.6 | 1.0669 | 1.0346 | 1.1002 |
| Nakhon Si Thammarat | 0-14 | 10.3 | 90th | 12.2 | 1.0090 | 0.9868 | 1.0317 |
| Nakhon Si Thammarat | 0-14 | 10.3 | 99th | 19.2 | 1.0400 | 0.9965 | 1.0855 |
| Nakhon Si Thammarat | 0-21 | 26.0 | 1st | 3.3 | 1.4975 | 1.2098 | 1.8536 |
| Nakhon Si Thammarat | 0-21 | 26.0 | 10th | 6.6 | 1.1486 | 0.9970 | 1.3232 |
| Nakhon Si Thammarat | 0-21 | 26.0 | 90th | 12.2 | 1.0608 | 0.8952 | 1.2571 |
| Nakhon Si Thammarat | 0-21 | 26.0 | 99th | 19.2 | 1.0663 | 0.9183 | 1.2381 |
| Nan | 0-7 | 9.3 | 1st | 3.2 | 1.1002 | 1.0228 | 1.1834 |
| Nan | 0-7 | 9.3 | 10th | 6.9 | 1.0135 | 0.9958 | 1.0316 |
| Nan | 0-7 | 9.3 | 90th | 18.0 | 1.0877 | 1.0352 | 1.1427 |
| Nan | 0-7 | 9.3 | 99th | 21.6 | 1.1466 | 1.0762 | 1.2215 |
| Nan | 0-14 | 9.8 | 1st | 3.2 | 1.1408 | 1.0435 | 1.2472 |
| Nan | 0-14 | 9.8 | 10th | 6.9 | 1.0232 | 0.9961 | 1.0510 |
| Nan | 0-14 | 9.8 | 90th | 18.0 | 1.0757 | 1.0246 | 1.1293 |
| Nan | 0-14 | 9.8 | 99th | 21.6 | 1.1287 | 1.0538 | 1.2089 |
| Nan | 0-21 | 10.6 | 1st | 3.2 | 1.1494 | 1.0121 | 1.3053 |
| Nan | 0-21 | 10.6 | 10th | 6.9 | 1.0312 | 0.9860 | 1.0784 |
| Nan | 0-21 | 10.6 | 90th | 18.0 | 1.0489 | 0.9974 | 1.1031 |
| Nan | 0-21 | 10.6 | 99th | 21.6 | 1.0909 | 1.0204 | 1.1662 |
| Narathiwat | 0-7 | 9.1 | 1st | 3.5 | 1.1309 | 1.0481 | 1.2201 |
| Narathiwat | 0-7 | 9.1 | 10th | 6.1 | 1.0357 | 1.0077 | 1.0646 |
| Narathiwat | 0-7 | 9.1 | 90th | 11.0 | 1.0090 | 0.9901 | 1.0283 |
| Narathiwat | 0-7 | 9.1 | 99th | 12.8 | 1.0273 | 0.9905 | 1.0655 |
| Narathiwat | 0-14 | 26.0 | 1st | 3.5 | 1.1956 | 0.9430 | 1.5159 |
| Narathiwat | 0-14 | 26.0 | 10th | 6.1 | 1.0541 | 0.8793 | 1.2637 |
| Narathiwat | 0-14 | 26.0 | 90th | 11.0 | 1.0195 | 0.8460 | 1.2286 |
| Narathiwat | 0-14 | 26.0 | 99th | 12.8 | 1.0438 | 0.8529 | 1.2773 |
| Narathiwat | 0-21 | 26.0 | 1st | 3.5 | 1.3302 | 0.9837 | 1.7989 |
| Narathiwat | 0-21 | 26.0 | 10th | 6.1 | 1.1266 | 0.9168 | 1.3843 |
| Narathiwat | 0-21 | 26.0 | 90th | 11.0 | 1.0697 | 0.8725 | 1.3114 |
| Narathiwat | 0-21 | 26.0 | 99th | 12.8 | 1.0983 | 0.8734 | 1.3811 |
| Nong Bua Lam Phu | 0-7 | 9.5 | 1st | 3.1 | 1.1228 | 1.0302 | 1.2237 |
| Nong Bua Lam Phu | 0-7 | 9.5 | 10th | 6.6 | 1.0239 | 0.9989 | 1.0497 |
| Nong Bua Lam Phu | 0-7 | 9.5 | 90th | 15.6 | 1.0571 | 1.0050 | 1.1119 |
| Nong Bua Lam Phu | 0-7 | 9.5 | 99th | 19.1 | 1.1185 | 1.0562 | 1.1846 |
| Nong Bua Lam Phu | 0-14 | 11.0 | 1st | 3.1 | 1.1653 | 1.0501 | 1.2932 |
| Nong Bua Lam Phu | 0-14 | 11.0 | 10th | 6.6 | 1.0464 | 0.9968 | 1.0984 |
| Nong Bua Lam Phu | 0-14 | 11.0 | 90th | 15.6 | 1.0315 | 0.9896 | 1.0752 |
| Nong Bua Lam Phu | 0-14 | 11.0 | 99th | 19.1 | 1.0872 | 1.0358 | 1.1412 |
| Nong Bua Lam Phu | 0-21 | 13.8 | 1st | 3.1 | 1.2011 | 1.0376 | 1.3904 |
| Nong Bua Lam Phu | 0-21 | 13.8 | 10th | 6.6 | 1.0910 | 0.9831 | 1.2107 |
| Nong Bua Lam Phu | 0-21 | 13.8 | 90th | 15.6 | 1.0050 | 0.9929 | 1.0173 |
| Nong Bua Lam Phu | 0-21 | 13.8 | 99th | 19.1 | 1.0406 | 0.9936 | 1.0898 |
| Nong Khai | 0-7 | 9.5 | 1st | 2.7 | 1.1822 | 1.0786 | 1.2958 |
| Nong Khai | 0-7 | 9.5 | 10th | 6.3 | 1.0379 | 1.0110 | 1.0656 |
| Nong Khai | 0-7 | 9.5 | 90th | 13.7 | 1.0346 | 0.9954 | 1.0754 |
| Nong Khai | 0-7 | 9.5 | 99th | 16.2 | 1.0698 | 1.0197 | 1.1225 |
| Nong Khai | 0-14 | 9.8 | 1st | 2.7 | 1.2443 | 1.1098 | 1.3951 |
| Nong Khai | 0-14 | 9.8 | 10th | 6.3 | 1.0522 | 1.0166 | 1.0890 |
| Nong Khai | 0-14 | 9.8 | 90th | 13.7 | 1.0282 | 0.9817 | 1.0770 |
| Nong Khai | 0-14 | 9.8 | 99th | 16.2 | 1.0512 | 0.9968 | 1.1084 |
| Nong Khai | 0-21 | 10.5 | 1st | 2.7 | 1.3149 | 1.1121 | 1.5546 |
| Nong Khai | 0-21 | 10.5 | 10th | 6.3 | 1.0751 | 1.0208 | 1.1322 |
| Nong Khai | 0-21 | 10.5 | 90th | 13.7 | 1.0129 | 0.9622 | 1.0663 |
| Nong Khai | 0-21 | 10.5 | 99th | 16.2 | 1.0209 | 0.9614 | 1.0841 |
| Pathum Thani | 0-7 | 11.8 | 1st | 4.6 | 1.0950 | 1.0350 | 1.1585 |
| Pathum Thani | 0-7 | 11.8 | 10th | 7.1 | 1.0388 | 1.0035 | 1.0754 |
| Pathum Thani | 0-7 | 11.8 | 90th | 12.5 | 1.0009 | 0.9950 | 1.0067 |
| Pathum Thani | 0-7 | 11.8 | 99th | 14.7 | 1.0136 | 0.9946 | 1.0329 |
| Pathum Thani | 0-14 | 13.0 | 1st | 4.6 | 1.1504 | 1.0701 | 1.2367 |
| Pathum Thani | 0-14 | 13.0 | 10th | 7.1 | 1.0732 | 1.0145 | 1.1353 |
| Pathum Thani | 0-14 | 13.0 | 90th | 12.5 | 1.0005 | 0.9957 | 1.0052 |
| Pathum Thani | 0-14 | 13.0 | 99th | 14.7 | 1.0061 | 0.9953 | 1.0171 |
| Pathum Thani | 0-21 | 14.5 | 1st | 4.6 | 1.2103 | 1.0930 | 1.3403 |
| Pathum Thani | 0-21 | 14.5 | 10th | 7.1 | 1.1216 | 1.0303 | 1.2210 |
| Pathum Thani | 0-21 | 14.5 | 90th | 12.5 | 1.0090 | 0.9896 | 1.0287 |
| Pathum Thani | 0-21 | 14.5 | 99th | 14.7 | 1.0001 | 0.9989 | 1.0013 |
| Pattani | 0-7 | 13.0 | 1st | 2.7 | 1.1196 | 1.0214 | 1.2274 |
| Pattani | 0-7 | 13.0 | 10th | 6.4 | 1.0506 | 1.0014 | 1.1023 |
| Pattani | 0-7 | 13.0 | 90th | 11.3 | 1.0034 | 0.9884 | 1.0187 |
| Pattani | 0-7 | 13.0 | 99th | 13.4 | 1.0002 | 0.9973 | 1.0031 |
| Pattani | 0-14 | 13.1 | 1st | 2.7 | 1.2078 | 1.0817 | 1.3485 |
| Pattani | 0-14 | 13.1 | 10th | 6.4 | 1.0726 | 1.0064 | 1.1432 |
| Pattani | 0-14 | 13.1 | 90th | 11.3 | 1.0036 | 0.9828 | 1.0249 |
| Pattani | 0-14 | 13.1 | 99th | 13.4 | 1.0001 | 0.9975 | 1.0027 |
| Pattani | 0-21 | 13.1 | 1st | 2.7 | 1.2953 | 1.1094 | 1.5123 |
| Pattani | 0-21 | 13.1 | 10th | 6.4 | 1.0907 | 1.0027 | 1.1865 |
| Pattani | 0-21 | 13.1 | 90th | 11.3 | 1.0030 | 0.9731 | 1.0338 |
| Pattani | 0-21 | 13.1 | 99th | 13.4 | 1.0001 | 0.9963 | 1.0038 |
| Phangnga | 0-7 | 9.1 | 1st | 3.0 | 1.1494 | 1.0515 | 1.2563 |
| Phangnga | 0-7 | 9.1 | 10th | 5.1 | 1.0635 | 1.0193 | 1.1096 |
| Phangnga | 0-7 | 9.1 | 90th | 10.1 | 1.0025 | 0.9921 | 1.0130 |
| Phangnga | 0-7 | 9.1 | 99th | 13.1 | 1.0326 | 0.9895 | 1.0776 |
| Phangnga | 0-14 | 9.7 | 1st | 3.0 | 1.1921 | 1.0667 | 1.3322 |
| Phangnga | 0-14 | 9.7 | 10th | 5.1 | 1.0856 | 1.0281 | 1.1464 |
| Phangnga | 0-14 | 9.7 | 90th | 10.1 | 1.0006 | 0.9947 | 1.0065 |
| Phangnga | 0-14 | 9.7 | 99th | 13.1 | 1.0236 | 0.9750 | 1.0745 |
| Phangnga | 0-21 | 11.4 | 1st | 3.0 | 1.2297 | 1.0492 | 1.4413 |
| Phangnga | 0-21 | 11.4 | 10th | 5.1 | 1.1173 | 1.0256 | 1.2172 |
| Phangnga | 0-21 | 11.4 | 90th | 10.1 | 1.0031 | 0.9755 | 1.0315 |
| Phangnga | 0-21 | 11.4 | 99th | 13.1 | 1.0036 | 0.9714 | 1.0368 |
| Phatthalung | 0-7 | 12.2 | 1st | 2.1 | 1.2624 | 1.1385 | 1.3998 |
| Phatthalung | 0-7 | 12.2 | 10th | 5.1 | 1.1191 | 1.0631 | 1.1779 |
| Phatthalung | 0-7 | 12.2 | 90th | 10.3 | 1.0062 | 0.9877 | 1.0249 |
| Phatthalung | 0-7 | 12.2 | 99th | 12.0 | 1.0001 | 0.9982 | 1.0019 |
| Phatthalung | 0-14 | 11.7 | 1st | 2.1 | 1.4028 | 1.2349 | 1.5935 |
| Phatthalung | 0-14 | 11.7 | 10th | 5.1 | 1.1621 | 1.0944 | 1.2341 |
| Phatthalung | 0-14 | 11.7 | 90th | 10.3 | 1.0040 | 0.9850 | 1.0234 |
| Phatthalung | 0-14 | 11.7 | 99th | 12.0 | 1.0001 | 0.9962 | 1.0040 |
| Phatthalung | 0-21 | 26.0 | 1st | 2.1 | 1.6929 | 1.1851 | 2.4184 |
| Phatthalung | 0-21 | 26.0 | 10th | 5.1 | 1.2878 | 1.0281 | 1.6132 |
| Phatthalung | 0-21 | 26.0 | 90th | 10.3 | 1.0607 | 0.8788 | 1.2801 |
| Phatthalung | 0-21 | 26.0 | 99th | 12.0 | 1.0613 | 0.8592 | 1.3109 |
| Phayao | 0-7 | 9.0 | 1st | 3.1 | 1.1674 | 1.0819 | 1.2595 |
| Phayao | 0-7 | 9.0 | 10th | 6.9 | 1.0189 | 1.0027 | 1.0354 |
| Phayao | 0-7 | 9.0 | 90th | 16.9 | 1.0794 | 1.0281 | 1.1333 |
| Phayao | 0-7 | 9.0 | 99th | 20.5 | 1.1046 | 1.0447 | 1.1680 |
| Phayao | 0-14 | 9.3 | 1st | 3.1 | 1.2313 | 1.1213 | 1.3520 |
| Phayao | 0-14 | 9.3 | 10th | 6.9 | 1.0294 | 1.0067 | 1.0525 |
| Phayao | 0-14 | 9.3 | 90th | 16.9 | 1.0681 | 1.0121 | 1.1272 |
| Phayao | 0-14 | 9.3 | 99th | 20.5 | 1.0767 | 1.0164 | 1.1405 |
| Phayao | 0-21 | 9.8 | 1st | 3.1 | 1.2814 | 1.1188 | 1.4677 |
| Phayao | 0-21 | 9.8 | 10th | 6.9 | 1.0427 | 1.0075 | 1.0790 |
| Phayao | 0-21 | 9.8 | 90th | 16.9 | 1.0445 | 0.9781 | 1.1153 |
| Phayao | 0-21 | 9.8 | 99th | 20.5 | 1.0429 | 0.9851 | 1.1042 |
| Phetchabun | 0-7 | 8.8 | 1st | 4.5 | 1.0588 | 1.0119 | 1.1078 |
| Phetchabun | 0-7 | 8.8 | 10th | 7.5 | 1.0047 | 0.9960 | 1.0135 |
| Phetchabun | 0-7 | 8.8 | 90th | 15.3 | 1.0623 | 1.0178 | 1.1086 |
| Phetchabun | 0-7 | 8.8 | 99th | 18.3 | 1.1057 | 1.0558 | 1.1579 |
| Phetchabun | 0-14 | 10.2 | 1st | 4.5 | 1.0819 | 1.0218 | 1.1455 |
| Phetchabun | 0-14 | 10.2 | 10th | 7.5 | 1.0157 | 0.9922 | 1.0398 |
| Phetchabun | 0-14 | 10.2 | 90th | 15.3 | 1.0334 | 0.9928 | 1.0756 |
| Phetchabun | 0-14 | 10.2 | 99th | 18.3 | 1.0731 | 1.0301 | 1.1179 |
| Phetchabun | 0-21 | 13.7 | 1st | 4.5 | 1.1153 | 1.0212 | 1.2181 |
| Phetchabun | 0-21 | 13.7 | 10th | 7.5 | 1.0551 | 0.9814 | 1.1342 |
| Phetchabun | 0-21 | 13.7 | 90th | 15.3 | 1.0036 | 0.9945 | 1.0128 |
| Phetchabun | 0-21 | 13.7 | 99th | 18.3 | 1.0291 | 1.0013 | 1.0577 |
| Phetchaburi | 0-7 | 9.3 | 1st | 3.4 | 1.0849 | 0.9988 | 1.1785 |
| Phetchaburi | 0-7 | 9.3 | 10th | 5.8 | 1.0293 | 0.9958 | 1.0640 |
| Phetchaburi | 0-7 | 9.3 | 90th | 10.2 | 1.0014 | 0.9935 | 1.0095 |
| Phetchaburi | 0-7 | 9.3 | 99th | 12.3 | 1.0143 | 0.9868 | 1.0424 |
| Phetchaburi | 0-14 | 10.7 | 1st | 3.4 | 1.1251 | 1.0179 | 1.2434 |
| Phetchaburi | 0-14 | 10.7 | 10th | 5.8 | 1.0531 | 1.0050 | 1.1036 |
| Phetchaburi | 0-14 | 10.7 | 90th | 10.2 | 1.0004 | 0.9939 | 1.0070 |
| Phetchaburi | 0-14 | 10.7 | 99th | 12.3 | 1.0039 | 0.9836 | 1.0246 |
| Phetchaburi | 0-21 | 13.4 | 1st | 3.4 | 1.1551 | 1.0070 | 1.3250 |
| Phetchaburi | 0-21 | 13.4 | 10th | 5.8 | 1.0892 | 0.9992 | 1.1873 |
| Phetchaburi | 0-21 | 13.4 | 90th | 10.2 | 1.0156 | 0.9633 | 1.0707 |
| Phetchaburi | 0-21 | 13.4 | 99th | 12.3 | 1.0018 | 0.9864 | 1.0175 |
| Phichit | 0-7 | 9.0 | 1st | 4.0 | 1.0890 | 1.0251 | 1.1569 |
| Phichit | 0-7 | 9.0 | 10th | 6.9 | 1.0146 | 0.9978 | 1.0317 |
| Phichit | 0-7 | 9.0 | 90th | 13.5 | 1.0403 | 1.0006 | 1.0816 |
| Phichit | 0-7 | 9.0 | 99th | 16.0 | 1.0773 | 1.0265 | 1.1306 |
| Phichit | 0-14 | 9.5 | 1st | 4.0 | 1.1081 | 1.0289 | 1.1935 |
| Phichit | 0-14 | 9.5 | 10th | 6.9 | 1.0222 | 0.9975 | 1.0475 |
| Phichit | 0-14 | 9.5 | 90th | 13.5 | 1.0287 | 0.9829 | 1.0767 |
| Phichit | 0-14 | 9.5 | 99th | 16.0 | 1.0598 | 1.0048 | 1.1179 |
| Phichit | 0-21 | 11.8 | 1st | 4.0 | 1.1256 | 1.0136 | 1.2500 |
| Phichit | 0-21 | 11.8 | 10th | 6.9 | 1.0448 | 0.9820 | 1.1116 |
| Phichit | 0-21 | 11.8 | 90th | 13.5 | 1.0036 | 0.9798 | 1.0280 |
| Phichit | 0-21 | 11.8 | 99th | 16.0 | 1.0201 | 0.9845 | 1.0571 |
| Phitsanulok | 0-7 | 8.7 | 1st | 4.1 | 1.0895 | 1.0342 | 1.1477 |
| Phitsanulok | 0-7 | 8.7 | 10th | 7.0 | 1.0117 | 0.9992 | 1.0243 |
| Phitsanulok | 0-7 | 8.7 | 90th | 13.4 | 1.0460 | 1.0078 | 1.0856 |
| Phitsanulok | 0-7 | 8.7 | 99th | 16.3 | 1.0847 | 1.0352 | 1.1366 |
| Phitsanulok | 0-14 | 9.1 | 1st | 4.1 | 1.1156 | 1.0462 | 1.1897 |
| Phitsanulok | 0-14 | 9.1 | 10th | 7.0 | 1.0178 | 0.9994 | 1.0364 |
| Phitsanulok | 0-14 | 9.1 | 90th | 13.4 | 1.0396 | 0.9937 | 1.0876 |
| Phitsanulok | 0-14 | 9.1 | 99th | 16.3 | 1.0702 | 1.0149 | 1.1285 |
| Phitsanulok | 0-21 | 9.8 | 1st | 4.1 | 1.1333 | 1.0324 | 1.2441 |
| Phitsanulok | 0-21 | 9.8 | 10th | 7.0 | 1.0275 | 0.9962 | 1.0597 |
| Phitsanulok | 0-21 | 9.8 | 90th | 13.4 | 1.0198 | 0.9686 | 1.0738 |
| Phitsanulok | 0-21 | 9.8 | 99th | 16.3 | 1.0372 | 0.9748 | 1.1037 |
| Phra Nakhon Si Ayutthaya | 0-7 | 9.5 | 1st | 5.0 | 1.0773 | 1.0325 | 1.1241 |
| Phra Nakhon Si Ayutthaya | 0-7 | 9.5 | 10th | 8.0 | 1.0074 | 0.9962 | 1.0187 |
| Phra Nakhon Si Ayutthaya | 0-7 | 9.5 | 90th | 13.9 | 1.0422 | 1.0064 | 1.0792 |
| Phra Nakhon Si Ayutthaya | 0-7 | 9.5 | 99th | 17.0 | 1.0992 | 1.0536 | 1.1468 |
| Phra Nakhon Si Ayutthaya | 0-14 | 10.3 | 1st | 5.0 | 1.1013 | 1.0447 | 1.1610 |
| Phra Nakhon Si Ayutthaya | 0-14 | 10.3 | 10th | 8.0 | 1.0156 | 0.9921 | 1.0396 |
| Phra Nakhon Si Ayutthaya | 0-14 | 10.3 | 90th | 13.9 | 1.0243 | 0.9867 | 1.0634 |
| Phra Nakhon Si Ayutthaya | 0-14 | 10.3 | 99th | 17.0 | 1.0654 | 1.0216 | 1.1109 |
| Phra Nakhon Si Ayutthaya | 0-21 | 12.7 | 1st | 5.0 | 1.1376 | 1.0461 | 1.2371 |
| Phra Nakhon Si Ayutthaya | 0-21 | 12.7 | 10th | 8.0 | 1.0410 | 0.9730 | 1.1138 |
| Phra Nakhon Si Ayutthaya | 0-21 | 12.7 | 90th | 13.9 | 1.0016 | 0.9883 | 1.0150 |
| Phra Nakhon Si Ayutthaya | 0-21 | 12.7 | 99th | 17.0 | 1.0174 | 0.9930 | 1.0425 |
| Phrae | 0-7 | 8.6 | 1st | 2.8 | 1.2017 | 1.1088 | 1.3024 |
| Phrae | 0-7 | 8.6 | 10th | 6.8 | 1.0180 | 1.0037 | 1.0325 |
| Phrae | 0-7 | 8.6 | 90th | 16.5 | 1.1185 | 1.0625 | 1.1774 |
| Phrae | 0-7 | 8.6 | 99th | 20.0 | 1.1586 | 1.0953 | 1.2256 |
| Phrae | 0-14 | 8.4 | 1st | 2.8 | 1.2487 | 1.1314 | 1.3783 |
| Phrae | 0-14 | 8.4 | 10th | 6.8 | 1.0192 | 1.0036 | 1.0350 |
| Phrae | 0-14 | 8.4 | 90th | 16.5 | 1.1311 | 1.0630 | 1.2036 |
| Phrae | 0-14 | 8.4 | 99th | 20.0 | 1.1392 | 1.0734 | 1.2090 |
| Phrae | 0-21 | 8.5 | 1st | 2.8 | 1.2883 | 1.1133 | 1.4909 |
| Phrae | 0-21 | 8.5 | 10th | 6.8 | 1.0231 | 1.0013 | 1.0453 |
| Phrae | 0-21 | 8.5 | 90th | 16.5 | 1.1260 | 1.0370 | 1.2226 |
| Phrae | 0-21 | 8.5 | 99th | 20.0 | 1.1167 | 1.0443 | 1.1941 |
| Phuket | 0-7 | 9.2 | 1st | 3.7 | 1.1028 | 1.0220 | 1.1900 |
| Phuket | 0-7 | 9.2 | 10th | 5.8 | 1.0393 | 1.0036 | 1.0762 |
| Phuket | 0-7 | 9.2 | 90th | 10.0 | 1.0016 | 0.9941 | 1.0091 |
| Phuket | 0-7 | 9.2 | 99th | 11.6 | 1.0118 | 0.9871 | 1.0370 |
| Phuket | 0-14 | 9.0 | 1st | 3.7 | 1.1534 | 1.0503 | 1.2668 |
| Phuket | 0-14 | 9.0 | 10th | 5.8 | 1.0561 | 1.0145 | 1.0995 |
| Phuket | 0-14 | 9.0 | 90th | 10.0 | 1.0031 | 0.9903 | 1.0160 |
| Phuket | 0-14 | 9.0 | 99th | 11.6 | 1.0187 | 0.9821 | 1.0567 |
| Phuket | 0-21 | 26.0 | 1st | 3.7 | 1.2222 | 0.8943 | 1.6703 |
| Phuket | 0-21 | 26.0 | 10th | 5.8 | 1.0894 | 0.8658 | 1.3707 |
| Phuket | 0-21 | 26.0 | 90th | 10.0 | 1.0164 | 0.8308 | 1.2435 |
| Phuket | 0-21 | 26.0 | 99th | 11.6 | 1.0354 | 0.8263 | 1.2975 |
| Prachin Buri | 0-7 | 10.8 | 1st | 4.2 | 1.1006 | 1.0320 | 1.1737 |
| Prachin Buri | 0-7 | 10.8 | 10th | 7.0 | 1.0313 | 1.0000 | 1.0636 |
| Prachin Buri | 0-7 | 10.8 | 90th | 12.9 | 1.0072 | 0.9870 | 1.0277 |
| Prachin Buri | 0-7 | 10.8 | 99th | 15.5 | 1.0338 | 0.9991 | 1.0698 |
| Prachin Buri | 0-14 | 12.4 | 1st | 4.2 | 1.1505 | 1.0612 | 1.2473 |
| Prachin Buri | 0-14 | 12.4 | 10th | 7.0 | 1.0614 | 1.0015 | 1.1248 |
| Prachin Buri | 0-14 | 12.4 | 90th | 12.9 | 1.0005 | 0.9950 | 1.0061 |
| Prachin Buri | 0-14 | 12.4 | 99th | 15.5 | 1.0179 | 0.9967 | 1.0394 |
| Prachin Buri | 0-21 | 14.4 | 1st | 4.2 | 1.2126 | 1.0808 | 1.3606 |
| Prachin Buri | 0-21 | 14.4 | 10th | 7.0 | 1.1144 | 1.0114 | 1.2280 |
| Prachin Buri | 0-21 | 14.4 | 90th | 12.9 | 1.0048 | 0.9893 | 1.0206 |
| Prachin Buri | 0-21 | 14.4 | 99th | 15.5 | 1.0024 | 0.9959 | 1.0088 |
| Prachuab Khiri Khan | 0-7 | 10.0 | 1st | 3.7 | 1.1381 | 1.0540 | 1.2288 |
| Prachuab Khiri Khan | 0-7 | 10.0 | 10th | 6.4 | 1.0415 | 1.0108 | 1.0731 |
| Prachuab Khiri Khan | 0-7 | 10.0 | 90th | 11.1 | 1.0024 | 0.9917 | 1.0132 |
| Prachuab Khiri Khan | 0-7 | 10.0 | 99th | 13.0 | 1.0156 | 0.9874 | 1.0446 |
| Prachuab Khiri Khan | 0-14 | 11.0 | 1st | 3.7 | 1.1973 | 1.0925 | 1.3122 |
| Prachuab Khiri Khan | 0-14 | 11.0 | 10th | 6.4 | 1.0681 | 1.0213 | 1.1171 |
| Prachuab Khiri Khan | 0-14 | 11.0 | 90th | 11.1 | 1.0000 | 0.9986 | 1.0014 |
| Prachuab Khiri Khan | 0-14 | 11.0 | 99th | 13.0 | 1.0068 | 0.9822 | 1.0320 |
| Prachuab Khiri Khan | 0-21 | 13.4 | 1st | 3.7 | 1.2627 | 1.1116 | 1.4343 |
| Prachuab Khiri Khan | 0-21 | 13.4 | 10th | 6.4 | 1.1113 | 1.0185 | 1.2125 |
| Prachuab Khiri Khan | 0-21 | 13.4 | 90th | 11.1 | 1.0068 | 0.9699 | 1.0451 |
| Prachuab Khiri Khan | 0-21 | 13.4 | 99th | 13.0 | 1.0001 | 0.9956 | 1.0046 |
| Ranong | 0-7 | 9.5 | 1st | 2.3 | 1.1937 | 1.0739 | 1.3268 |
| Ranong | 0-7 | 9.5 | 10th | 4.9 | 1.0779 | 1.0276 | 1.1307 |
| Ranong | 0-7 | 9.5 | 90th | 11.4 | 1.0081 | 0.9858 | 1.0309 |
| Ranong | 0-7 | 9.5 | 99th | 13.8 | 1.0325 | 0.9857 | 1.0815 |
| Ranong | 0-14 | 9.8 | 1st | 2.3 | 1.2615 | 1.1057 | 1.4393 |
| Ranong | 0-14 | 9.8 | 10th | 4.9 | 1.1062 | 1.0421 | 1.1741 |
| Ranong | 0-14 | 9.8 | 90th | 11.4 | 1.0062 | 0.9805 | 1.0326 |
| Ranong | 0-14 | 9.8 | 99th | 13.8 | 1.0274 | 0.9715 | 1.0865 |
| Ranong | 0-21 | 10.6 | 1st | 2.3 | 1.3279 | 1.0916 | 1.6154 |
| Ranong | 0-21 | 10.6 | 10th | 4.9 | 1.1404 | 1.0453 | 1.2442 |
| Ranong | 0-21 | 10.6 | 90th | 11.4 | 1.0012 | 0.9829 | 1.0199 |
| Ranong | 0-21 | 10.6 | 99th | 13.8 | 1.0126 | 0.9510 | 1.0782 |
| Ratchaburi | 0-7 | 11.5 | 1st | 2.8 | 1.0910 | 1.0061 | 1.1831 |
| Ratchaburi | 0-7 | 11.5 | 10th | 6.2 | 1.0364 | 1.0021 | 1.0719 |
| Ratchaburi | 0-7 | 11.5 | 90th | 12.7 | 1.0024 | 0.9927 | 1.0122 |
| Ratchaburi | 0-7 | 11.5 | 99th | 15.6 | 1.0274 | 1.0034 | 1.0520 |
| Ratchaburi | 0-14 | 13.1 | 1st | 2.8 | 1.1605 | 1.0552 | 1.2765 |
| Ratchaburi | 0-14 | 13.1 | 10th | 6.2 | 1.0840 | 1.0256 | 1.1457 |
| Ratchaburi | 0-14 | 13.1 | 90th | 12.7 | 1.0004 | 0.9969 | 1.0040 |
| Ratchaburi | 0-14 | 13.1 | 99th | 15.6 | 1.0137 | 1.0006 | 1.0271 |
| Ratchaburi | 0-21 | 14.8 | 1st | 2.8 | 1.2335 | 1.0770 | 1.4126 |
| Ratchaburi | 0-21 | 14.8 | 10th | 6.2 | 1.1622 | 1.0701 | 1.2622 |
| Ratchaburi | 0-21 | 14.8 | 90th | 12.7 | 1.0129 | 0.9951 | 1.0310 |
| Ratchaburi | 0-21 | 14.8 | 99th | 15.6 | 1.0020 | 0.9978 | 1.0063 |
| Rayong | 0-7 | 9.9 | 1st | 2.8 | 1.0793 | 0.9950 | 1.1707 |
| Rayong | 0-7 | 9.9 | 10th | 4.5 | 1.0471 | 0.9992 | 1.0972 |
| Rayong | 0-7 | 9.9 | 90th | 9.9 | 1.0000 | 1.0000 | 1.0000 |
| Rayong | 0-7 | 9.9 | 99th | 12.2 | 1.0075 | 0.9867 | 1.0288 |
| Rayong | 0-14 | 12.2 | 1st | 2.8 | 1.1166 | 1.0169 | 1.2261 |
| Rayong | 0-14 | 12.2 | 10th | 4.5 | 1.0818 | 1.0177 | 1.1499 |
| Rayong | 0-14 | 12.2 | 90th | 9.9 | 1.0083 | 0.9796 | 1.0379 |
| Rayong | 0-14 | 12.2 | 99th | 12.2 | 1.0000 | 0.9994 | 1.0006 |
| Rayong | 0-21 | 14.7 | 1st | 2.8 | 1.1782 | 1.0301 | 1.3474 |
| Rayong | 0-21 | 14.7 | 10th | 4.5 | 1.1574 | 1.0540 | 1.2711 |
| Rayong | 0-21 | 14.7 | 90th | 9.9 | 1.0568 | 0.9892 | 1.1290 |
| Rayong | 0-21 | 14.7 | 99th | 12.2 | 1.0173 | 0.9898 | 1.0455 |
| Roi Et | 0-7 | 10.4 | 1st | 2.7 | 1.1430 | 1.0681 | 1.2232 |
| Roi Et | 0-7 | 10.4 | 10th | 6.2 | 1.0414 | 1.0160 | 1.0674 |
| Roi Et | 0-7 | 10.4 | 90th | 13.7 | 1.0175 | 0.9942 | 1.0412 |
| Roi Et | 0-7 | 10.4 | 99th | 16.6 | 1.0569 | 1.0241 | 1.0908 |
| Roi Et | 0-14 | 12.1 | 1st | 2.7 | 1.2013 | 1.1082 | 1.3023 |
| Roi Et | 0-14 | 12.1 | 10th | 6.2 | 1.0749 | 1.0287 | 1.1232 |
| Roi Et | 0-14 | 12.1 | 90th | 13.7 | 1.0038 | 0.9917 | 1.0160 |
| Roi Et | 0-14 | 12.1 | 99th | 16.6 | 1.0299 | 1.0084 | 1.0518 |
| Roi Et | 0-21 | 14.8 | 1st | 2.7 | 1.2824 | 1.1439 | 1.4377 |
| Roi Et | 0-21 | 14.8 | 10th | 6.2 | 1.1449 | 1.0606 | 1.2359 |
| Roi Et | 0-21 | 14.8 | 90th | 13.7 | 1.0024 | 0.9955 | 1.0094 |
| Roi Et | 0-21 | 14.8 | 99th | 16.6 | 1.0053 | 0.9965 | 1.0142 |
| Sa Kaeo | 0-7 | 10.1 | 1st | 4.0 | 1.0750 | 1.0076 | 1.1468 |
| Sa Kaeo | 0-7 | 10.1 | 10th | 7.0 | 1.0193 | 0.9928 | 1.0465 |
| Sa Kaeo | 0-7 | 10.1 | 90th | 13.0 | 1.0138 | 0.9846 | 1.0439 |
| Sa Kaeo | 0-7 | 10.1 | 99th | 15.9 | 1.0509 | 1.0056 | 1.0981 |
| Sa Kaeo | 0-14 | 12.6 | 1st | 4.0 | 1.1319 | 1.0400 | 1.2320 |
| Sa Kaeo | 0-14 | 12.6 | 10th | 7.0 | 1.0616 | 0.9962 | 1.1312 |
| Sa Kaeo | 0-14 | 12.6 | 90th | 13.0 | 1.0003 | 0.9960 | 1.0047 |
| Sa Kaeo | 0-14 | 12.6 | 99th | 15.9 | 1.0237 | 1.0025 | 1.0453 |
| Sa Kaeo | 0-21 | 14.7 | 1st | 4.0 | 1.2074 | 1.0683 | 1.3647 |
| Sa Kaeo | 0-21 | 14.7 | 10th | 7.0 | 1.1490 | 1.0349 | 1.2758 |
| Sa Kaeo | 0-21 | 14.7 | 90th | 13.0 | 1.0097 | 0.9929 | 1.0268 |
| Sa Kaeo | 0-21 | 14.7 | 99th | 15.9 | 1.0040 | 0.9970 | 1.0111 |
| Sakon Nakhon | 0-7 | 9.2 | 1st | 2.6 | 1.2281 | 1.1446 | 1.3177 |
| Sakon Nakhon | 0-7 | 9.2 | 10th | 6.2 | 1.0432 | 1.0232 | 1.0635 |
| Sakon Nakhon | 0-7 | 9.2 | 90th | 14.7 | 1.0682 | 1.0291 | 1.1088 |
| Sakon Nakhon | 0-7 | 9.2 | 99th | 17.4 | 1.1126 | 1.0676 | 1.1595 |
| Sakon Nakhon | 0-14 | 9.4 | 1st | 2.6 | 1.2825 | 1.1758 | 1.3989 |
| Sakon Nakhon | 0-14 | 9.4 | 10th | 6.2 | 1.0558 | 1.0299 | 1.0823 |
| Sakon Nakhon | 0-14 | 9.4 | 90th | 14.7 | 1.0558 | 1.0087 | 1.1050 |
| Sakon Nakhon | 0-14 | 9.4 | 99th | 17.4 | 1.0835 | 1.0355 | 1.1336 |
| Sakon Nakhon | 0-21 | 10.2 | 1st | 2.6 | 1.3380 | 1.1806 | 1.5163 |
| Sakon Nakhon | 0-21 | 10.2 | 10th | 6.2 | 1.0785 | 1.0368 | 1.1219 |
| Sakon Nakhon | 0-21 | 10.2 | 90th | 14.7 | 1.0248 | 0.9733 | 1.0790 |
| Sakon Nakhon | 0-21 | 10.2 | 99th | 17.4 | 1.0342 | 0.9831 | 1.0880 |
| Samut Prakan | 0-7 | 6.9 | 1st | 2.2 | 1.0233 | 0.9493 | 1.1030 |
| Samut Prakan | 0-7 | 6.9 | 10th | 3.9 | 1.0107 | 0.9703 | 1.0527 |
| Samut Prakan | 0-7 | 6.9 | 90th | 7.9 | 1.0012 | 0.9950 | 1.0075 |
| Samut Prakan | 0-7 | 6.9 | 99th | 9.7 | 1.0101 | 0.9937 | 1.0267 |
| Samut Prakan | 0-14 | 10.3 | 1st | 2.2 | 1.0302 | 0.9333 | 1.1371 |
| Samut Prakan | 0-14 | 10.3 | 10th | 3.9 | 1.0213 | 0.9634 | 1.0828 |
| Samut Prakan | 0-14 | 10.3 | 90th | 7.9 | 1.0043 | 0.9814 | 1.0277 |
| Samut Prakan | 0-14 | 10.3 | 99th | 9.7 | 1.0003 | 0.9934 | 1.0073 |
| Samut Prakan | 0-21 | 0.1 | 1st | 2.2 | 1.0442 | 0.9453 | 1.1533 |
| Samut Prakan | 0-21 | 0.1 | 10th | 3.9 | 1.0745 | 0.9075 | 1.2721 |
| Samut Prakan | 0-21 | 0.1 | 90th | 7.9 | 1.0967 | 0.8490 | 1.4166 |
| Samut Prakan | 0-21 | 0.1 | 99th | 9.7 | 1.0773 | 0.8383 | 1.3844 |
| Samut Songkhram | 0-7 | 9.6 | 1st | 3.3 | 1.1173 | 1.0137 | 1.2314 |
| Samut Songkhram | 0-7 | 9.6 | 10th | 4.7 | 1.0703 | 1.0058 | 1.1389 |
| Samut Songkhram | 0-7 | 9.6 | 90th | 10.5 | 1.0020 | 0.9910 | 1.0130 |
| Samut Songkhram | 0-7 | 9.6 | 99th | 13.0 | 1.0218 | 0.9808 | 1.0645 |
| Samut Songkhram | 0-14 | 10.5 | 1st | 3.3 | 1.1586 | 1.0270 | 1.3071 |
| Samut Songkhram | 0-14 | 10.5 | 10th | 4.7 | 1.1003 | 1.0170 | 1.1904 |
| Samut Songkhram | 0-14 | 10.5 | 90th | 10.5 | 1.0000 | 1.0000 | 1.0000 |
| Samut Songkhram | 0-14 | 10.5 | 99th | 13.0 | 1.0100 | 0.9709 | 1.0508 |
| Samut Songkhram | 0-21 | 13.2 | 1st | 3.3 | 1.1997 | 1.0141 | 1.4193 |
| Samut Songkhram | 0-21 | 13.2 | 10th | 4.7 | 1.1406 | 1.0090 | 1.2894 |
| Samut Songkhram | 0-21 | 13.2 | 90th | 10.5 | 1.0101 | 0.9501 | 1.0738 |
| Samut Songkhram | 0-21 | 13.2 | 99th | 13.0 | 1.0000 | 0.9966 | 1.0034 |
| Satun | 0-7 | 11.8 | 1st | 3.3 | 1.1578 | 1.0634 | 1.2605 |
| Satun | 0-7 | 11.8 | 10th | 6.3 | 1.0626 | 1.0142 | 1.1134 |
| Satun | 0-7 | 11.8 | 90th | 11.2 | 1.0006 | 0.9939 | 1.0073 |
| Satun | 0-7 | 11.8 | 99th | 14.4 | 1.0115 | 0.9892 | 1.0344 |
| Satun | 0-14 | 13.1 | 1st | 3.3 | 1.2489 | 1.1237 | 1.3882 |
| Satun | 0-14 | 13.1 | 10th | 6.3 | 1.1124 | 1.0320 | 1.1991 |
| Satun | 0-14 | 13.1 | 90th | 11.2 | 1.0077 | 0.9822 | 1.0338 |
| Satun | 0-14 | 13.1 | 99th | 14.4 | 1.0035 | 0.9931 | 1.0140 |
| Satun | 0-21 | 14.8 | 1st | 3.3 | 1.3429 | 1.1562 | 1.5598 |
| Satun | 0-21 | 14.8 | 10th | 6.3 | 1.1763 | 1.0499 | 1.3180 |
| Satun | 0-21 | 14.8 | 90th | 11.2 | 1.0283 | 0.9747 | 1.0849 |
| Satun | 0-21 | 14.8 | 99th | 14.4 | 1.0003 | 0.9974 | 1.0031 |
| Si Sa Ket | 0-7 | 11.3 | 1st | 3.1 | 1.0565 | 0.9873 | 1.1304 |
| Si Sa Ket | 0-7 | 11.3 | 10th | 6.4 | 1.0255 | 0.9948 | 1.0572 |
| Si Sa Ket | 0-7 | 11.3 | 90th | 14.2 | 1.0149 | 0.9953 | 1.0348 |
| Si Sa Ket | 0-7 | 11.3 | 99th | 17.3 | 1.0666 | 1.0363 | 1.0977 |
| Si Sa Ket | 0-14 | 13.2 | 1st | 3.1 | 1.1158 | 1.0288 | 1.2101 |
| Si Sa Ket | 0-14 | 13.2 | 10th | 6.4 | 1.0789 | 1.0235 | 1.1373 |
| Si Sa Ket | 0-14 | 13.2 | 90th | 14.2 | 1.0029 | 0.9968 | 1.0090 |
| Si Sa Ket | 0-14 | 13.2 | 99th | 17.3 | 1.0476 | 1.0272 | 1.0685 |
| Si Sa Ket | 0-21 | 14.8 | 1st | 3.1 | 1.1736 | 1.0476 | 1.3148 |
| Si Sa Ket | 0-21 | 14.8 | 10th | 6.4 | 1.1690 | 1.0821 | 1.2628 |
| Si Sa Ket | 0-21 | 14.8 | 90th | 14.2 | 1.0012 | 0.9979 | 1.0045 |
| Si Sa Ket | 0-21 | 14.8 | 99th | 17.3 | 1.0234 | 1.0085 | 1.0385 |
| Songkhla | 0-7 | 12.7 | 1st | 3.3 | 1.2292 | 1.1538 | 1.3095 |
| Songkhla | 0-7 | 12.7 | 10th | 6.4 | 1.0920 | 1.0517 | 1.1340 |
| Songkhla | 0-7 | 12.7 | 90th | 10.9 | 1.0063 | 0.9921 | 1.0207 |
| Songkhla | 0-7 | 12.7 | 99th | 12.8 | 1.0000 | 0.9994 | 1.0006 |
| Songkhla | 0-14 | 13.1 | 1st | 3.3 | 1.3375 | 1.2383 | 1.4447 |
| Songkhla | 0-14 | 13.1 | 10th | 6.4 | 1.1243 | 1.0669 | 1.1848 |
| Songkhla | 0-14 | 13.1 | 90th | 10.9 | 1.0082 | 0.9860 | 1.0309 |
| Songkhla | 0-14 | 13.1 | 99th | 12.8 | 1.0001 | 0.9976 | 1.0026 |
| Songkhla | 0-21 | 16.4 | 1st | 3.3 | 1.4697 | 1.3103 | 1.6486 |
| Songkhla | 0-21 | 16.4 | 10th | 6.4 | 1.1568 | 1.0666 | 1.2546 |
| Songkhla | 0-21 | 16.4 | 90th | 10.9 | 1.0095 | 0.9663 | 1.0546 |
| Songkhla | 0-21 | 16.4 | 99th | 12.8 | 1.0015 | 0.9818 | 1.0216 |
| Sukhothai | 0-7 | 10.7 | 1st | 3.8 | 1.1320 | 1.0614 | 1.2074 |
| Sukhothai | 0-7 | 10.7 | 10th | 7.0 | 1.0345 | 1.0066 | 1.0631 |
| Sukhothai | 0-7 | 10.7 | 90th | 14.1 | 1.0198 | 0.9923 | 1.0480 |
| Sukhothai | 0-7 | 10.7 | 99th | 16.7 | 1.0539 | 1.0171 | 1.0921 |
| Sukhothai | 0-14 | 12.3 | 1st | 3.8 | 1.1991 | 1.1081 | 1.2975 |
| Sukhothai | 0-14 | 12.3 | 10th | 7.0 | 1.0706 | 1.0169 | 1.1273 |
| Sukhothai | 0-14 | 12.3 | 90th | 14.1 | 1.0060 | 0.9912 | 1.0211 |
| Sukhothai | 0-14 | 12.3 | 99th | 16.7 | 1.0336 | 1.0094 | 1.0584 |
| Sukhothai | 0-21 | 14.5 | 1st | 3.8 | 1.2746 | 1.1395 | 1.4257 |
| Sukhothai | 0-21 | 14.5 | 10th | 7.0 | 1.1325 | 1.0386 | 1.2350 |
| Sukhothai | 0-21 | 14.5 | 90th | 14.1 | 1.0003 | 0.9976 | 1.0030 |
| Sukhothai | 0-21 | 14.5 | 99th | 16.7 | 1.0094 | 0.9972 | 1.0217 |
| Suphan Buri | 0-7 | 9.4 | 1st | 4.3 | 1.0644 | 1.0127 | 1.1187 |
| Suphan Buri | 0-7 | 9.4 | 10th | 7.5 | 1.0079 | 0.9952 | 1.0209 |
| Suphan Buri | 0-7 | 9.4 | 90th | 13.1 | 1.0245 | 0.9940 | 1.0560 |
| Suphan Buri | 0-7 | 9.4 | 99th | 15.9 | 1.0651 | 1.0227 | 1.1093 |
| Suphan Buri | 0-14 | 11.9 | 1st | 4.3 | 1.1023 | 1.0350 | 1.1740 |
| Suphan Buri | 0-14 | 11.9 | 10th | 7.5 | 1.0372 | 0.9936 | 1.0827 |
| Suphan Buri | 0-14 | 11.9 | 90th | 13.1 | 1.0029 | 0.9911 | 1.0149 |
| Suphan Buri | 0-14 | 11.9 | 99th | 15.9 | 1.0344 | 1.0102 | 1.0592 |
| Suphan Buri | 0-21 | 14.2 | 1st | 4.3 | 1.1515 | 1.0480 | 1.2651 |
| Suphan Buri | 0-21 | 14.2 | 10th | 7.5 | 1.1140 | 1.0277 | 1.2076 |
| Suphan Buri | 0-21 | 14.2 | 90th | 13.1 | 1.0048 | 0.9950 | 1.0147 |
| Suphan Buri | 0-21 | 14.2 | 99th | 15.9 | 1.0089 | 1.0000 | 1.0178 |
| Surat Thani | 0-7 | 11.7 | 1st | 3.6 | 1.1641 | 1.0939 | 1.2387 |
| Surat Thani | 0-7 | 11.7 | 10th | 6.5 | 1.0609 | 1.0259 | 1.0970 |
| Surat Thani | 0-7 | 11.7 | 90th | 11.2 | 1.0005 | 0.9959 | 1.0052 |
| Surat Thani | 0-7 | 11.7 | 99th | 13.0 | 1.0022 | 0.9920 | 1.0125 |
| Surat Thani | 0-14 | 11.3 | 1st | 3.6 | 1.2444 | 1.1552 | 1.3406 |
| Surat Thani | 0-14 | 11.3 | 10th | 6.5 | 1.0795 | 1.0373 | 1.1235 |
| Surat Thani | 0-14 | 11.3 | 90th | 11.2 | 1.0000 | 0.9983 | 1.0018 |
| Surat Thani | 0-14 | 11.3 | 99th | 13.0 | 1.0040 | 0.9862 | 1.0222 |
| Surat Thani | 0-21 | 26.0 | 1st | 3.6 | 1.3718 | 1.0694 | 1.7599 |
| Surat Thani | 0-21 | 26.0 | 10th | 6.5 | 1.1260 | 0.9492 | 1.3358 |
| Surat Thani | 0-21 | 26.0 | 90th | 11.2 | 1.0268 | 0.8596 | 1.2267 |
| Surat Thani | 0-21 | 26.0 | 99th | 13.0 | 1.0357 | 0.8499 | 1.2622 |
| Surin | 0-7 | 9.1 | 1st | 4.1 | 1.1013 | 1.0474 | 1.1580 |
| Surin | 0-7 | 9.1 | 10th | 7.3 | 1.0118 | 0.9999 | 1.0239 |
| Surin | 0-7 | 9.1 | 90th | 14.1 | 1.0539 | 1.0155 | 1.0937 |
| Surin | 0-7 | 9.1 | 99th | 16.9 | 1.1080 | 1.0609 | 1.1572 |
| Surin | 0-14 | 10.5 | 1st | 4.1 | 1.1204 | 1.0531 | 1.1920 |
| Surin | 0-14 | 10.5 | 10th | 7.3 | 1.0266 | 0.9984 | 1.0556 |
| Surin | 0-14 | 10.5 | 90th | 14.1 | 1.0255 | 0.9919 | 1.0602 |
| Surin | 0-14 | 10.5 | 99th | 16.9 | 1.0739 | 1.0345 | 1.1148 |
| Surin | 0-21 | 13.6 | 1st | 4.1 | 1.1499 | 1.0496 | 1.2598 |
| Surin | 0-21 | 13.6 | 10th | 7.3 | 1.0798 | 1.0023 | 1.1633 |
| Surin | 0-21 | 13.6 | 90th | 14.1 | 1.0006 | 0.9969 | 1.0043 |
| Surin | 0-21 | 13.6 | 99th | 16.9 | 1.0267 | 1.0094 | 1.0443 |
| Tak | 0-7 | 9.8 | 1st | 4.0 | 1.0874 | 1.0292 | 1.1488 |
| Tak | 0-7 | 9.8 | 10th | 6.4 | 1.0279 | 1.0023 | 1.0541 |
| Tak | 0-7 | 9.8 | 90th | 16.5 | 1.0502 | 1.0043 | 1.0982 |
| Tak | 0-7 | 9.8 | 99th | 19.5 | 1.0883 | 1.0332 | 1.1464 |
| Tak | 0-14 | 11.3 | 1st | 4.0 | 1.1444 | 1.0680 | 1.2263 |
| Tak | 0-14 | 11.3 | 10th | 6.4 | 1.0572 | 1.0086 | 1.1081 |
| Tak | 0-14 | 11.3 | 90th | 16.5 | 1.0258 | 0.9920 | 1.0608 |
| Tak | 0-14 | 11.3 | 99th | 19.5 | 1.0550 | 1.0083 | 1.1038 |
| Tak | 0-21 | 14.5 | 1st | 4.0 | 1.2177 | 1.0945 | 1.3547 |
| Tak | 0-21 | 14.5 | 10th | 6.4 | 1.1119 | 1.0100 | 1.2241 |
| Tak | 0-21 | 14.5 | 90th | 16.5 | 1.0039 | 0.9928 | 1.0150 |
| Tak | 0-21 | 14.5 | 99th | 19.5 | 1.0222 | 0.9753 | 1.0714 |
| Trang | 0-7 | 12.0 | 1st | 2.9 | 1.2423 | 1.1419 | 1.3514 |
| Trang | 0-7 | 12.0 | 10th | 6.5 | 1.0785 | 1.0355 | 1.1233 |
| Trang | 0-7 | 12.0 | 90th | 12.4 | 1.0003 | 0.9967 | 1.0039 |
| Trang | 0-7 | 12.0 | 99th | 15.0 | 1.0148 | 0.9948 | 1.0352 |
| Trang | 0-14 | 12.3 | 1st | 2.9 | 1.3420 | 1.2120 | 1.4860 |
| Trang | 0-14 | 12.3 | 10th | 6.5 | 1.1032 | 1.0426 | 1.1674 |
| Trang | 0-14 | 12.3 | 90th | 12.4 | 1.0000 | 0.9989 | 1.0012 |
| Trang | 0-14 | 12.3 | 99th | 15.0 | 1.0090 | 0.9894 | 1.0289 |
| Trang | 0-21 | 12.9 | 1st | 2.9 | 1.4694 | 1.2729 | 1.6962 |
| Trang | 0-21 | 12.9 | 10th | 6.5 | 1.1325 | 1.0437 | 1.2289 |
| Trang | 0-21 | 12.9 | 90th | 12.4 | 1.0002 | 0.9925 | 1.0079 |
| Trang | 0-21 | 12.9 | 99th | 15.0 | 1.0016 | 0.9832 | 1.0202 |
| Trat | 0-7 | 10.2 | 1st | 2.7 | 1.0988 | 0.9929 | 1.2161 |
| Trat | 0-7 | 10.2 | 10th | 5.3 | 1.0423 | 0.9950 | 1.0918 |
| Trat | 0-7 | 10.2 | 90th | 10.5 | 1.0001 | 0.9967 | 1.0035 |
| Trat | 0-7 | 10.2 | 99th | 12.9 | 1.0088 | 0.9794 | 1.0391 |
| Trat | 0-14 | 12.0 | 1st | 2.7 | 1.1693 | 1.0351 | 1.3209 |
| Trat | 0-14 | 12.0 | 10th | 5.3 | 1.0846 | 1.0114 | 1.1631 |
| Trat | 0-14 | 12.0 | 90th | 10.5 | 1.0035 | 0.9806 | 1.0269 |
| Trat | 0-14 | 12.0 | 99th | 12.9 | 1.0012 | 0.9895 | 1.0130 |
| Trat | 0-21 | 14.0 | 1st | 2.7 | 1.2169 | 1.0240 | 1.4462 |
| Trat | 0-21 | 14.0 | 10th | 5.3 | 1.1300 | 1.0122 | 1.2614 |
| Trat | 0-21 | 14.0 | 90th | 10.5 | 1.0211 | 0.9572 | 1.0892 |
| Trat | 0-21 | 14.0 | 99th | 12.9 | 1.0020 | 0.9875 | 1.0168 |
| Ubon Ratchathani | 0-7 | 10.7 | 1st | 3.5 | 1.1932 | 1.1287 | 1.2614 |
| Ubon Ratchathani | 0-7 | 10.7 | 10th | 7.3 | 1.0366 | 1.0160 | 1.0577 |
| Ubon Ratchathani | 0-7 | 10.7 | 90th | 15.1 | 1.0304 | 1.0053 | 1.0562 |
| Ubon Ratchathani | 0-7 | 10.7 | 99th | 17.7 | 1.0670 | 1.0348 | 1.1003 |
| Ubon Ratchathani | 0-14 | 11.5 | 1st | 3.5 | 1.2585 | 1.1779 | 1.3447 |
| Ubon Ratchathani | 0-14 | 11.5 | 10th | 7.3 | 1.0570 | 1.0236 | 1.0915 |
| Ubon Ratchathani | 0-14 | 11.5 | 90th | 15.1 | 1.0180 | 0.9961 | 1.0404 |
| Ubon Ratchathani | 0-14 | 11.5 | 99th | 17.7 | 1.0466 | 1.0189 | 1.0751 |
| Ubon Ratchathani | 0-21 | 13.6 | 1st | 3.5 | 1.3371 | 1.2191 | 1.4666 |
| Ubon Ratchathani | 0-21 | 13.6 | 10th | 7.3 | 1.0906 | 1.0260 | 1.1593 |
| Ubon Ratchathani | 0-21 | 13.6 | 90th | 15.1 | 1.0016 | 0.9938 | 1.0095 |
| Ubon Ratchathani | 0-21 | 13.6 | 99th | 17.7 | 1.0123 | 0.9936 | 1.0314 |
| Udon Thani | 0-7 | 9.6 | 1st | 2.8 | 1.1274 | 1.0511 | 1.2092 |
| Udon Thani | 0-7 | 9.6 | 10th | 6.4 | 1.0267 | 1.0068 | 1.0470 |
| Udon Thani | 0-7 | 9.6 | 90th | 14.9 | 1.0427 | 1.0078 | 1.0788 |
| Udon Thani | 0-7 | 9.6 | 99th | 18.0 | 1.0896 | 1.0484 | 1.1325 |
| Udon Thani | 0-14 | 10.5 | 1st | 2.8 | 1.1811 | 1.0856 | 1.2850 |
| Udon Thani | 0-14 | 10.5 | 10th | 6.4 | 1.0459 | 1.0143 | 1.0785 |
| Udon Thani | 0-14 | 10.5 | 90th | 14.9 | 1.0224 | 0.9883 | 1.0578 |
| Udon Thani | 0-14 | 10.5 | 99th | 18.0 | 1.0515 | 1.0147 | 1.0896 |
| Udon Thani | 0-21 | 13.5 | 1st | 2.8 | 1.2375 | 1.1039 | 1.3873 |
| Udon Thani | 0-21 | 13.5 | 10th | 6.4 | 1.0791 | 1.0063 | 1.1571 |
| Udon Thani | 0-21 | 13.5 | 90th | 14.9 | 1.0010 | 0.9923 | 1.0097 |
| Udon Thani | 0-21 | 13.5 | 99th | 18.0 | 1.0088 | 0.9862 | 1.0319 |
| Uthai Thani | 0-7 | 9.8 | 1st | 3.7 | 1.1355 | 1.0423 | 1.2371 |
| Uthai Thani | 0-7 | 9.8 | 10th | 7.2 | 1.0211 | 0.9941 | 1.0488 |
| Uthai Thani | 0-7 | 9.8 | 90th | 14.0 | 1.0307 | 0.9832 | 1.0805 |
| Uthai Thani | 0-7 | 9.8 | 99th | 16.3 | 1.0582 | 0.9981 | 1.1220 |
| Uthai Thani | 0-14 | 10.1 | 1st | 3.7 | 1.1885 | 1.0690 | 1.3212 |
| Uthai Thani | 0-14 | 10.1 | 10th | 7.2 | 1.0316 | 0.9935 | 1.0711 |
| Uthai Thani | 0-14 | 10.1 | 90th | 14.0 | 1.0236 | 0.9693 | 1.0811 |
| Uthai Thani | 0-14 | 10.1 | 99th | 16.3 | 1.0410 | 0.9795 | 1.1063 |
| Uthai Thani | 0-21 | 11.0 | 1st | 3.7 | 1.2531 | 1.0760 | 1.4594 |
| Uthai Thani | 0-21 | 11.0 | 10th | 7.2 | 1.0508 | 0.9849 | 1.1211 |
| Uthai Thani | 0-21 | 11.0 | 90th | 14.0 | 1.0091 | 0.9530 | 1.0684 |
| Uthai Thani | 0-21 | 11.0 | 99th | 16.3 | 1.0141 | 0.9508 | 1.0817 |
| Uttaradit | 0-7 | 8.8 | 1st | 3.2 | 1.1266 | 1.0453 | 1.2141 |
| Uttaradit | 0-7 | 8.8 | 10th | 7.2 | 1.0094 | 0.9969 | 1.0220 |
| Uttaradit | 0-7 | 8.8 | 90th | 14.8 | 1.0634 | 1.0143 | 1.1150 |
| Uttaradit | 0-7 | 8.8 | 99th | 17.6 | 1.0996 | 1.0436 | 1.1585 |
| Uttaradit | 0-14 | 8.9 | 1st | 3.2 | 1.1681 | 1.0651 | 1.2811 |
| Uttaradit | 0-14 | 8.9 | 10th | 7.2 | 1.0136 | 0.9973 | 1.0302 |
| Uttaradit | 0-14 | 8.9 | 90th | 14.8 | 1.0630 | 1.0022 | 1.1274 |
| Uttaradit | 0-14 | 8.9 | 99th | 17.6 | 1.0905 | 1.0288 | 1.1558 |
| Uttaradit | 0-21 | 9.2 | 1st | 3.2 | 1.1869 | 1.0364 | 1.3592 |
| Uttaradit | 0-21 | 9.2 | 10th | 7.2 | 1.0177 | 0.9932 | 1.0427 |
| Uttaradit | 0-21 | 9.2 | 90th | 14.8 | 1.0509 | 0.9742 | 1.1335 |
| Uttaradit | 0-21 | 9.2 | 99th | 17.6 | 1.0695 | 0.9946 | 1.1502 |
| Yala | 0-7 | 10.1 | 1st | 2.7 | 1.1202 | 1.0183 | 1.2323 |
| Yala | 0-7 | 10.1 | 10th | 6.6 | 1.0236 | 0.9949 | 1.0531 |
| Yala | 0-7 | 10.1 | 90th | 12.4 | 1.0055 | 0.9819 | 1.0297 |
| Yala | 0-7 | 10.1 | 99th | 15.4 | 1.0172 | 0.9753 | 1.0609 |
| Yala | 0-14 | 26.0 | 1st | 2.7 | 1.2198 | 0.9423 | 1.5790 |
| Yala | 0-14 | 26.0 | 10th | 6.6 | 1.0426 | 0.8754 | 1.2417 |
| Yala | 0-14 | 26.0 | 90th | 12.4 | 1.0258 | 0.8405 | 1.2520 |
| Yala | 0-14 | 26.0 | 99th | 15.4 | 1.0426 | 0.8498 | 1.2791 |
| Yala | 0-21 | 26.0 | 1st | 2.7 | 1.3373 | 0.9592 | 1.8644 |
| Yala | 0-21 | 26.0 | 10th | 6.6 | 1.0689 | 0.8840 | 1.2925 |
| Yala | 0-21 | 26.0 | 90th | 12.4 | 1.0593 | 0.8494 | 1.3211 |
| Yala | 0-21 | 26.0 | 99th | 15.4 | 1.0878 | 0.8579 | 1.3794 |
| Yasothon | 0-7 | 10.2 | 1st | 3.2 | 1.1439 | 1.0409 | 1.2571 |
| Yasothon | 0-7 | 10.2 | 10th | 7.1 | 1.0255 | 0.9945 | 1.0574 |
| Yasothon | 0-7 | 10.2 | 90th | 14.7 | 1.0306 | 0.9848 | 1.0786 |
| Yasothon | 0-7 | 10.2 | 99th | 17.2 | 1.0653 | 1.0080 | 1.1258 |
| Yasothon | 0-14 | 10.7 | 1st | 3.2 | 1.1997 | 1.0683 | 1.3472 |
| Yasothon | 0-14 | 10.7 | 10th | 7.1 | 1.0377 | 0.9906 | 1.0870 |
| Yasothon | 0-14 | 10.7 | 90th | 14.7 | 1.0201 | 0.9726 | 1.0699 |
| Yasothon | 0-14 | 10.7 | 99th | 17.2 | 1.0415 | 0.9903 | 1.0954 |
| Yasothon | 0-21 | 12.1 | 1st | 3.2 | 1.2693 | 1.0783 | 1.4941 |
| Yasothon | 0-21 | 12.1 | 10th | 7.1 | 1.0611 | 0.9717 | 1.1587 |
| Yasothon | 0-21 | 12.1 | 90th | 14.7 | 1.0039 | 0.9673 | 1.0419 |
| Yasothon | 0-21 | 12.1 | 99th | 17.2 | 1.0105 | 0.9716 | 1.0509 |

**Table S4** The province-specific mortality fractions attributable to non-minimum mortality DTR (non-MM-DTR) at different lag structures

| **Provinces** | **Lag** | **Total deaths** | **AF (%) and 95% eCI** | | |
| --- | --- | --- | --- | --- | --- |
|  |  |  | **AF** | **Lower** | **Upper** |
| Amnat Charoen | 0-7 | 4787 | 1.81 | 0.65 | 2.93 |
| Amnat Charoen | 0-14 | 4787 | 2.47 | 0.01 | 4.55 |
| Amnat Charoen | 0-21 | 4787 | 4.68 | -2.02 | 10.90 |
| Bangkok | 0-7 | 572161 | 0.93 | 0.24 | 1.64 |
| Bangkok | 0-14 | 572161 | 1.57 | 0.58 | 2.51 |
| Bangkok | 0-21 | 572161 | 3.28 | 0.53 | 6.13 |
| Bueng Kan | 0-7 | 5210 | 2.53 | 1.28 | 3.81 |
| Bueng Kan | 0-14 | 5210 | 3.29 | 2.07 | 4.60 |
| Bueng Kan | 0-21 | 5210 | 4.17 | 0.86 | 7.37 |
| Buri Ram | 0-7 | 126666 | 2.46 | 1.66 | 3.20 |
| Buri Ram | 0-14 | 126666 | 2.16 | 1.74 | 2.57 |
| Buri Ram | 0-21 | 126666 | 5.21 | 1.53 | 8.91 |
| Chachoengsao | 0-7 | 65590 | 1.77 | 0.58 | 2.95 |
| Chachoengsao | 0-14 | 65590 | 1.76 | 0.62 | 2.91 |
| Chachoengsao | 0-21 | 65590 | 2.45 | 0.27 | 4.60 |
| Chai Nat | 0-7 | 40054 | 1.28 | 0.40 | 2.09 |
| Chai Nat | 0-14 | 40054 | 1.21 | 0.68 | 1.79 |
| Chai Nat | 0-21 | 40054 | 1.92 | -0.86 | 4.24 |
| Chaiyaphum | 0-7 | 107456 | 1.40 | 0.79 | 1.99 |
| Chaiyaphum | 0-14 | 107456 | 1.98 | 1.42 | 2.52 |
| Chaiyaphum | 0-21 | 107456 | 8.75 | -5.88 | 22.24 |
| Chanthaburi | 0-7 | 52392 | 1.42 | 0.28 | 2.50 |
| Chanthaburi | 0-14 | 52392 | 3.07 | 0.17 | 5.72 |
| Chanthaburi | 0-21 | 52392 | 3.72 | -0.69 | 7.66 |
| Chiang Mai | 0-7 | 190405 | 1.62 | 0.70 | 2.48 |
| Chiang Mai | 0-14 | 190405 | 2.05 | 1.13 | 2.92 |
| Chiang Mai | 0-21 | 190405 | 5.91 | 1.52 | 10.14 |
| Chiang Rai | 0-7 | 118834 | 2.44 | 0.99 | 3.82 |
| Chiang Rai | 0-14 | 118834 | 3.38 | 1.68 | 5.02 |
| Chiang Rai | 0-21 | 118834 | 9.11 | -2.76 | 19.29 |
| Chon Buri | 0-7 | 131238 | 0.79 | -0.29 | 1.85 |
| Chon Buri | 0-14 | 131238 | 1.47 | 0.02 | 2.78 |
| Chon Buri | 0-21 | 131238 | 2.04 | -1.84 | 5.96 |
| Chumphon | 0-7 | 41354 | 3.20 | 0.29 | 5.81 |
| Chumphon | 0-14 | 41354 | 5.47 | 0.72 | 9.91 |
| Chumphon | 0-21 | 41354 | 6.85 | -0.37 | 14.25 |
| Kalasin | 0-7 | 92945 | 2.06 | 0.98 | 3.11 |
| Kalasin | 0-14 | 92945 | 2.58 | 1.70 | 3.38 |
| Kalasin | 0-21 | 92945 | 5.09 | -8.65 | 18.67 |
| Kamphaeng Phet | 0-7 | 65578 | 1.39 | 0.55 | 2.25 |
| Kamphaeng Phet | 0-14 | 65578 | 2.34 | 0.29 | 4.37 |
| Kamphaeng Phet | 0-21 | 65578 | 5.20 | 0.32 | 9.68 |
| Kanchanaburi | 0-7 | 66705 | 2.01 | 0.02 | 3.89 |
| Kanchanaburi | 0-14 | 66705 | 2.53 | -0.35 | 5.07 |
| Kanchanaburi | 0-21 | 66705 | 2.50 | -16.30 | 16.96 |
| Khon Kaen | 0-7 | 180625 | 1.96 | 1.06 | 2.88 |
| Khon Kaen | 0-14 | 180625 | 1.92 | 1.48 | 2.31 |
| Khon Kaen | 0-21 | 180625 | 4.71 | 1.38 | 7.90 |
| Krabi | 0-7 | 28650 | 1.69 | 0.54 | 2.70 |
| Krabi | 0-14 | 28650 | 2.32 | 1.00 | 3.64 |
| Krabi | 0-21 | 28650 | 5.73 | -2.80 | 13.23 |
| Lampang | 0-7 | 96275 | 5.42 | 3.35 | 7.48 |
| Lampang | 0-14 | 96275 | 4.91 | 2.51 | 7.08 |
| Lampang | 0-21 | 96275 | 2.39 | 1.10 | 3.57 |
| Lamphun | 0-7 | 52424 | 3.79 | 1.36 | 5.97 |
| Lamphun | 0-14 | 52424 | 4.06 | 1.33 | 6.65 |
| Lamphun | 0-21 | 52424 | 2.03 | 1.09 | 2.93 |
| Loei | 0-7 | 57890 | 2.30 | 1.18 | 3.36 |
| Loei | 0-14 | 57890 | 2.67 | 1.77 | 3.64 |
| Loei | 0-21 | 57890 | 2.25 | -0.47 | 4.85 |
| Lop Buri | 0-7 | 81014 | 1.10 | 0.61 | 1.53 |
| Lop Buri | 0-14 | 81014 | 1.89 | -0.25 | 3.82 |
| Lop Buri | 0-21 | 81014 | 6.33 | 1.39 | 10.97 |
| Mae Hong Son | 0-7 | 16637 | 2.80 | 0.81 | 4.51 |
| Mae Hong Son | 0-14 | 16637 | 3.11 | 0.97 | 5.04 |
| Mae Hong Son | 0-21 | 16637 | 5.03 | 0.39 | 9.36 |
| Maha Sarakham | 0-7 | 89456 | 1.25 | 0.46 | 2.02 |
| Maha Sarakham | 0-14 | 89456 | 1.52 | 0.68 | 2.30 |
| Maha Sarakham | 0-21 | 89456 | 2.26 | -1.79 | 6.11 |
| Mukdahan | 0-7 | 28791 | 1.91 | 0.42 | 3.18 |
| Mukdahan | 0-14 | 28791 | 1.92 | 1.16 | 2.53 |
| Mukdahan | 0-21 | 28791 | 3.99 | -1.82 | 9.69 |
| Nakhon Nayok | 0-7 | 7031 | 2.04 | -0.63 | 4.58 |
| Nakhon Nayok | 0-14 | 7031 | 4.63 | 1.23 | 7.60 |
| Nakhon Nayok | 0-21 | 7031 | 8.72 | -2.34 | 17.82 |
| Nakhon Pathom | 0-7 | 79145 | 1.04 | -0.07 | 2.03 |
| Nakhon Pathom | 0-14 | 79145 | 1.58 | -0.35 | 3.61 |
| Nakhon Pathom | 0-21 | 79145 | 3.86 | -0.95 | 8.32 |
| Nakhon Phanom | 0-7 | 61780 | 2.29 | 1.36 | 3.20 |
| Nakhon Phanom | 0-14 | 61780 | 2.90 | 1.39 | 4.26 |
| Nakhon Phanom | 0-21 | 61780 | 5.32 | 0.46 | 9.45 |
| Nakhon Ratchasima | 0-7 | 238325 | 1.73 | 1.20 | 2.23 |
| Nakhon Ratchasima | 0-14 | 238325 | 1.91 | 1.24 | 2.60 |
| Nakhon Ratchasima | 0-21 | 238325 | 6.01 | 2.18 | 9.52 |
| Nakhon Sawan | 0-7 | 111512 | 2.24 | 0.98 | 3.51 |
| Nakhon Sawan | 0-14 | 111512 | 2.17 | 0.50 | 3.91 |
| Nakhon Sawan | 0-21 | 111512 | 1.68 | -0.04 | 3.31 |
| Nakhon Si Thammarat | 0-7 | 124851 | 1.77 | 1.04 | 2.51 |
| Nakhon Si Thammarat | 0-14 | 124851 | 1.95 | 1.47 | 2.39 |
| Nakhon Si Thammarat | 0-21 | 124851 | 7.95 | -5.56 | 20.55 |
| Nan | 0-7 | 44907 | 3.07 | 0.79 | 5.34 |
| Nan | 0-14 | 44907 | 3.11 | 1.76 | 4.38 |
| Nan | 0-21 | 44907 | 2.47 | 0.93 | 3.99 |
| Narathiwat | 0-7 | 60713 | 0.80 | 0.04 | 1.46 |
| Narathiwat | 0-14 | 60713 | 1.48 | 0.62 | 2.29 |
| Narathiwat | 0-21 | 60713 | 7.48 | -10.91 | 24.23 |
| Nong Bua Lam Phu | 0-7 | 23451 | 2.44 | 0.65 | 4.19 |
| Nong Bua Lam Phu | 0-14 | 23451 | 2.37 | 1.52 | 3.14 |
| Nong Bua Lam Phu | 0-21 | 23451 | 3.48 | -1.60 | 7.95 |
| Nong Khai | 0-7 | 52639 | 1.70 | 0.44 | 2.86 |
| Nong Khai | 0-14 | 52639 | 2.25 | 0.95 | 3.42 |
| Nong Khai | 0-21 | 52639 | 2.61 | 1.17 | 4.00 |
| Pathum Thani | 0-7 | 84603 | 1.02 | 0.32 | 1.72 |
| Pathum Thani | 0-14 | 84603 | 2.76 | -0.64 | 5.97 |
| Pathum Thani | 0-21 | 84603 | 5.45 | 0.09 | 10.54 |
| Pattani | 0-7 | 57143 | 2.34 | -0.60 | 5.04 |
| Pattani | 0-14 | 57143 | 3.19 | -1.16 | 7.14 |
| Pattani | 0-21 | 57143 | 3.60 | -1.73 | 8.99 |
| Phangnga | 0-7 | 20113 | 1.80 | 0.50 | 3.10 |
| Phangnga | 0-14 | 20113 | 2.41 | 1.18 | 3.65 |
| Phangnga | 0-21 | 20113 | 4.41 | -0.55 | 9.39 |
| Phatthalung | 0-7 | 41026 | 4.37 | 0.89 | 7.59 |
| Phatthalung | 0-14 | 41026 | 5.55 | 0.52 | 9.96 |
| Phatthalung | 0-21 | 41026 | 12.17 | -6.42 | 27.61 |
| Phayao | 0-7 | 54750 | 2.70 | 1.03 | 4.38 |
| Phayao | 0-14 | 54750 | 3.10 | 0.93 | 5.11 |
| Phayao | 0-21 | 54750 | 2.88 | 0.67 | 4.97 |
| Phetchabun | 0-7 | 95728 | 2.31 | 0.52 | 3.87 |
| Phetchabun | 0-14 | 95728 | 1.99 | 0.34 | 3.79 |
| Phetchabun | 0-21 | 95728 | 2.24 | -1.07 | 5.38 |
| Phetchaburi | 0-7 | 45310 | 0.79 | 0.07 | 1.50 |
| Phetchaburi | 0-14 | 45310 | 1.72 | 0.06 | 3.33 |
| Phetchaburi | 0-21 | 45310 | 4.74 | -2.05 | 11.22 |
| Phichit | 0-7 | 57161 | 1.48 | 0.40 | 2.61 |
| Phichit | 0-14 | 57161 | 1.66 | 0.11 | 3.06 |
| Phichit | 0-21 | 57161 | 1.69 | -0.76 | 3.96 |
| Phitsanulok | 0-7 | 90958 | 1.66 | 0.36 | 2.89 |
| Phitsanulok | 0-14 | 90958 | 2.06 | 0.29 | 3.75 |
| Phitsanulok | 0-21 | 90958 | 1.47 | 0.10 | 2.74 |
| Phra Nakhon Si Ayutthaya | 0-7 | 82514 | 1.64 | 0.45 | 2.73 |
| Phra Nakhon Si Ayutthaya | 0-14 | 82514 | 1.39 | 0.44 | 2.39 |
| Phra Nakhon Si Ayutthaya | 0-21 | 82514 | 1.57 | -1.29 | 4.30 |
| Phrae | 0-7 | 59988 | 4.12 | 1.95 | 6.17 |
| Phrae | 0-14 | 59988 | 5.66 | 2.68 | 8.35 |
| Phrae | 0-21 | 59988 | 5.83 | 2.29 | 9.67 |
| Phuket | 0-7 | 26048 | 1.01 | -0.89 | 2.68 |
| Phuket | 0-14 | 26048 | 1.41 | 0.56 | 2.31 |
| Phuket | 0-21 | 26048 | 3.65 | -19.11 | 20.63 |
| Prachin Buri | 0-7 | 45629 | 1.24 | 0.36 | 2.10 |
| Prachin Buri | 0-14 | 45629 | 2.45 | -0.84 | 5.27 |
| Prachin Buri | 0-21 | 45629 | 5.18 | -0.46 | 10.31 |
| Prachuab Khiri Khan | 0-7 | 42254 | 1.24 | 0.21 | 2.20 |
| Prachuab Khiri Khan | 0-14 | 42254 | 1.65 | 0.79 | 2.40 |
| Prachuab Khiri Khan | 0-21 | 42254 | 4.58 | -1.44 | 10.74 |
| Ranong | 0-7 | 11601 | 2.35 | 0.65 | 3.91 |
| Ranong | 0-14 | 11601 | 3.22 | 1.65 | 4.72 |
| Ranong | 0-21 | 11601 | 4.48 | 1.24 | 7.53 |
| Ratchaburi | 0-7 | 88017 | 1.06 | 0.08 | 2.01 |
| Ratchaburi | 0-14 | 88017 | 3.26 | 0.02 | 6.36 |
| Ratchaburi | 0-21 | 88017 | 7.77 | 2.39 | 12.37 |
| Rayong | 0-7 | 55964 | 1.93 | -0.14 | 4.10 |
| Rayong | 0-14 | 55964 | 3.18 | 0.27 | 6.01 |
| Rayong | 0-21 | 55964 | 10.37 | 2.34 | 17.15 |
| Roi Et | 0-7 | 122523 | 1.58 | 0.91 | 2.31 |
| Roi Et | 0-14 | 122523 | 2.94 | 0.66 | 5.14 |
| Roi Et | 0-21 | 122523 | 5.96 | 1.71 | 10.37 |
| Sa Kaeo | 0-7 | 44247 | 1.12 | 0.32 | 1.91 |
| Sa Kaeo | 0-14 | 44247 | 2.18 | -0.70 | 4.80 |
| Sa Kaeo | 0-21 | 44247 | 7.19 | 1.04 | 12.84 |
| Sakon Nakhon | 0-7 | 99218 | 3.01 | 1.73 | 4.15 |
| Sakon Nakhon | 0-14 | 99218 | 3.16 | 1.99 | 4.30 |
| Sakon Nakhon | 0-21 | 99218 | 3.09 | 1.68 | 4.47 |
| Samut Prakan | 0-7 | 100721 | 0.69 | -1.03 | 2.25 |
| Samut Prakan | 0-14 | 100721 | 1.19 | -1.67 | 3.84 |
| Samut Prakan | 0-21 | 100721 | 8.20 | -12.30 | 26.35 |
| Samut Songkhram | 0-7 | 4673 | 2.32 | -0.11 | 4.86 |
| Samut Songkhram | 0-14 | 4673 | 3.80 | 0.72 | 6.75 |
| Samut Songkhram | 0-21 | 4673 | 6.65 | -1.63 | 15.29 |
| Satun | 0-7 | 20544 | 1.78 | 0.05 | 3.68 |
| Satun | 0-14 | 20544 | 4.79 | -0.26 | 9.85 |
| Satun | 0-21 | 20544 | 8.64 | 0.30 | 15.88 |
| Si Sa Ket | 0-7 | 121547 | 1.52 | 0.25 | 2.81 |
| Si Sa Ket | 0-14 | 121547 | 3.44 | 0.59 | 6.03 |
| Si Sa Ket | 0-21 | 121547 | 7.55 | 3.30 | 11.40 |
| Songkhla | 0-7 | 121129 | 4.07 | 1.62 | 6.32 |
| Songkhla | 0-14 | 121129 | 4.56 | 0.26 | 8.74 |
| Songkhla | 0-21 | 121129 | 6.06 | -0.87 | 11.43 |
| Sukhothai | 0-7 | 63074 | 1.57 | 0.97 | 2.12 |
| Sukhothai | 0-14 | 63074 | 2.32 | 0.54 | 4.07 |
| Sukhothai | 0-21 | 63074 | 5.26 | 1.19 | 9.44 |
| Suphan Buri | 0-7 | 92186 | 1.52 | 0.37 | 2.66 |
| Suphan Buri | 0-14 | 92186 | 1.16 | 0.74 | 1.56 |
| Suphan Buri | 0-21 | 92186 | 5.26 | 0.86 | 9.47 |
| Surat Thani | 0-7 | 78117 | 2.37 | 0.04 | 4.48 |
| Surat Thani | 0-14 | 78117 | 2.68 | -0.54 | 5.62 |
| Surat Thani | 0-21 | 78117 | 5.73 | -11.21 | 19.99 |
| Surin | 0-7 | 120784 | 2.13 | 1.08 | 3.14 |
| Surin | 0-14 | 120784 | 1.80 | 1.07 | 2.53 |
| Surin | 0-21 | 120784 | 3.35 | -0.41 | 6.90 |
| Tak | 0-7 | 40711 | 2.45 | 0.49 | 4.12 |
| Tak | 0-14 | 40711 | 2.16 | 1.26 | 2.99 |
| Tak | 0-21 | 40711 | 3.78 | -0.87 | 8.19 |
| Trang | 0-7 | 49401 | 3.49 | 1.13 | 5.84 |
| Trang | 0-14 | 49401 | 4.00 | 0.47 | 7.65 |
| Trang | 0-21 | 49401 | 4.68 | -0.03 | 9.11 |
| Trat | 0-7 | 18728 | 1.21 | -0.12 | 2.51 |
| Trat | 0-14 | 18728 | 3.41 | -1.32 | 7.58 |
| Trat | 0-21 | 18728 | 6.17 | -2.81 | 14.05 |
| Ubon Ratchathani | 0-7 | 159424 | 1.97 | 1.26 | 2.54 |
| Ubon Ratchathani | 0-14 | 159424 | 2.54 | 1.14 | 3.80 |
| Ubon Ratchathani | 0-21 | 159424 | 3.41 | 0.76 | 6.16 |
| Udon Thani | 0-7 | 134737 | 2.24 | 1.08 | 3.32 |
| Udon Thani | 0-14 | 134737 | 2.23 | 1.58 | 2.82 |
| Udon Thani | 0-21 | 134737 | 2.69 | -0.57 | 5.70 |
| Uthai Thani | 0-7 | 7433 | 1.44 | 0.10 | 2.73 |
| Uthai Thani | 0-14 | 7433 | 1.70 | 0.27 | 2.87 |
| Uthai Thani | 0-21 | 7433 | 1.93 | 0.92 | 2.88 |
| Uttaradit | 0-7 | 53426 | 1.76 | 0.35 | 3.11 |
| Uttaradit | 0-14 | 53426 | 2.11 | 0.21 | 3.79 |
| Uttaradit | 0-21 | 53426 | 2.49 | -0.51 | 5.35 |
| Yala | 0-7 | 37815 | 2.17 | -0.82 | 5.23 |
| Yala | 0-14 | 37815 | 1.57 | -1.02 | 3.89 |
| Yala | 0-21 | 37815 | 4.87 | -15.71 | 21.87 |
| Yasothon | 0-7 | 8114 | 1.53 | 0.56 | 2.35 |
| Yasothon | 0-14 | 8114 | 1.79 | 1.05 | 2.52 |
| Yasothon | 0-21 | 8114 | 2.32 | -0.30 | 4.66 |
| Thailand (72 provinces) | 0-7 | 5574850 | 1.88 | 0.69 | 3.03 |
| Thailand (72 provinces) | 0-14 | 5574850 | 2.39 | 0.75 | 3.99 |
| Thailand (72 provinces) | 0-21 | 5574850 | 4.67 | -1.14 | 9.87 |


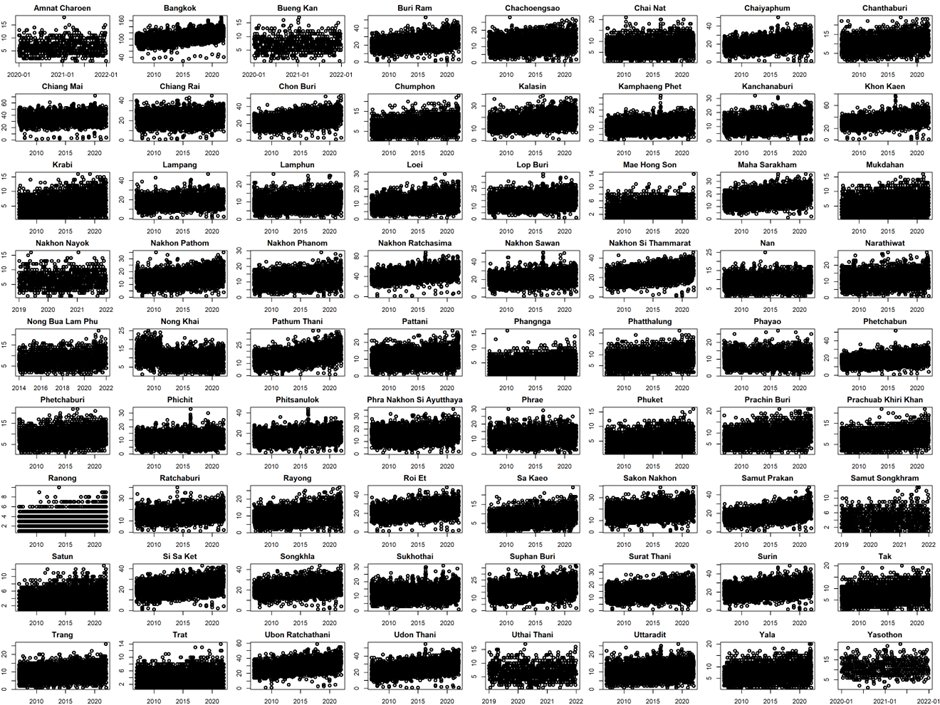


**Fig. S1** Daily number of deaths over time for each province included in this study

**
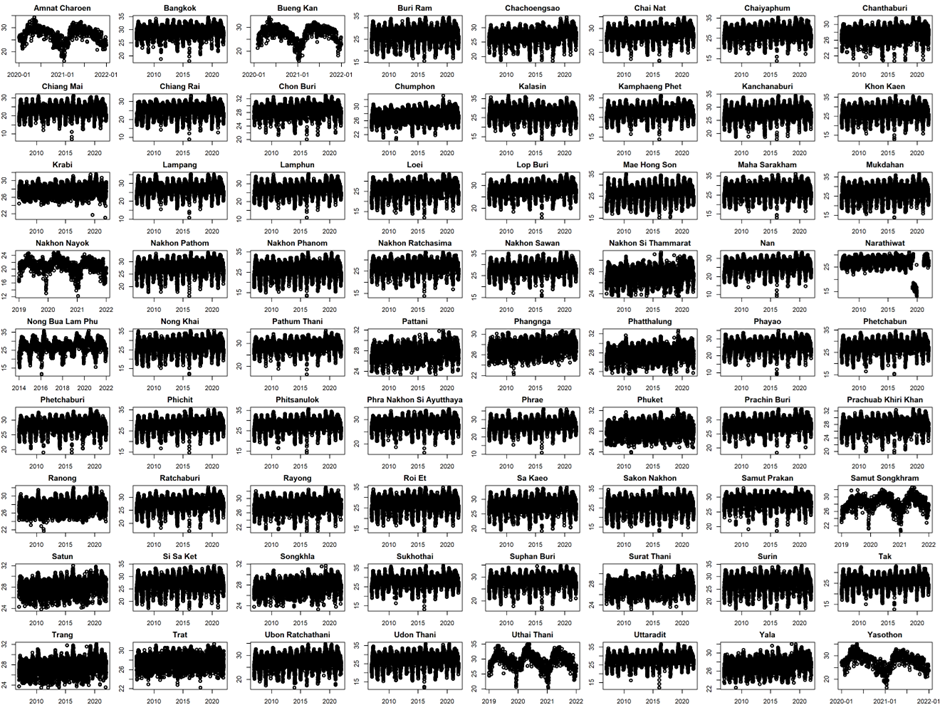
Fig. S2** Daily mean temperature over time for each province included in this study


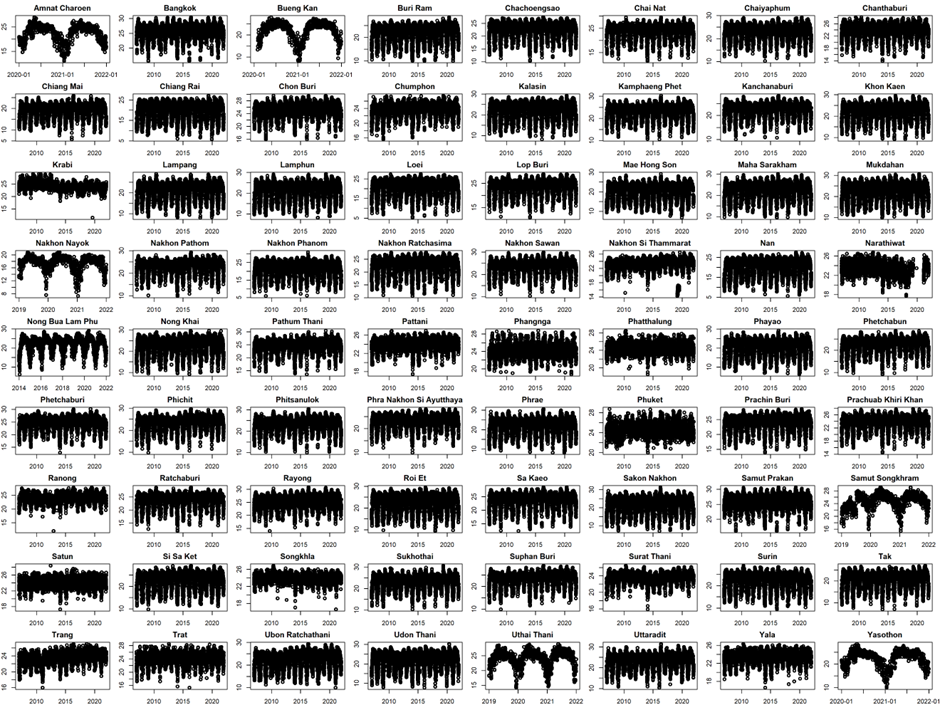
**Fig. S3** Daily minimum temperature over time for each province included in this study


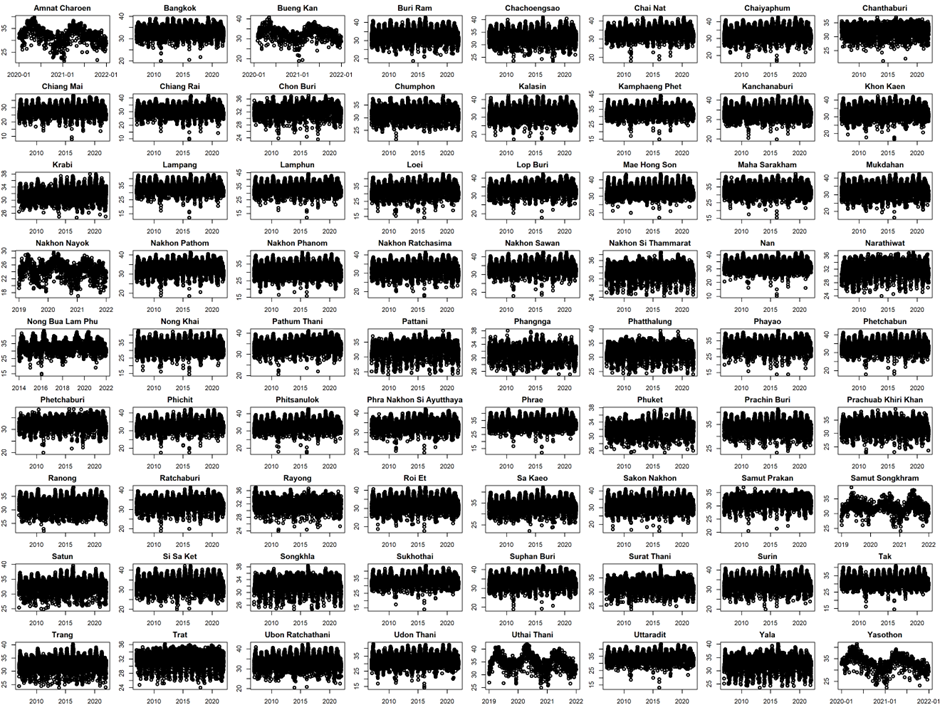
**Fig. S4** Daily maximum temperature over time for each province included in this study

**
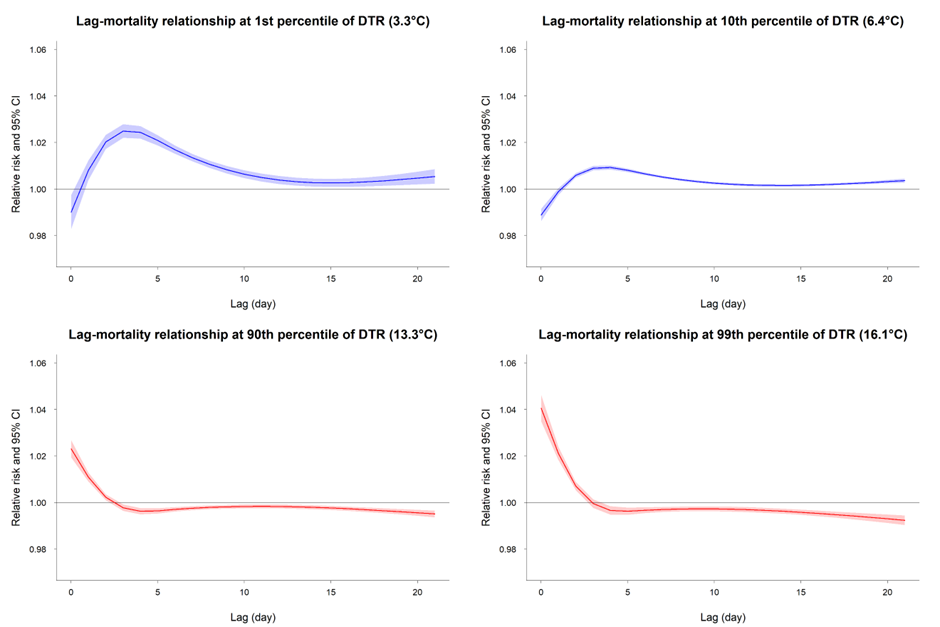
Fig. S5** The pooled relative risk (RR) of all-cause mortality by lag at 1st, 10th, 90th and 99th percentiles of diurnal temperature range (DTR)


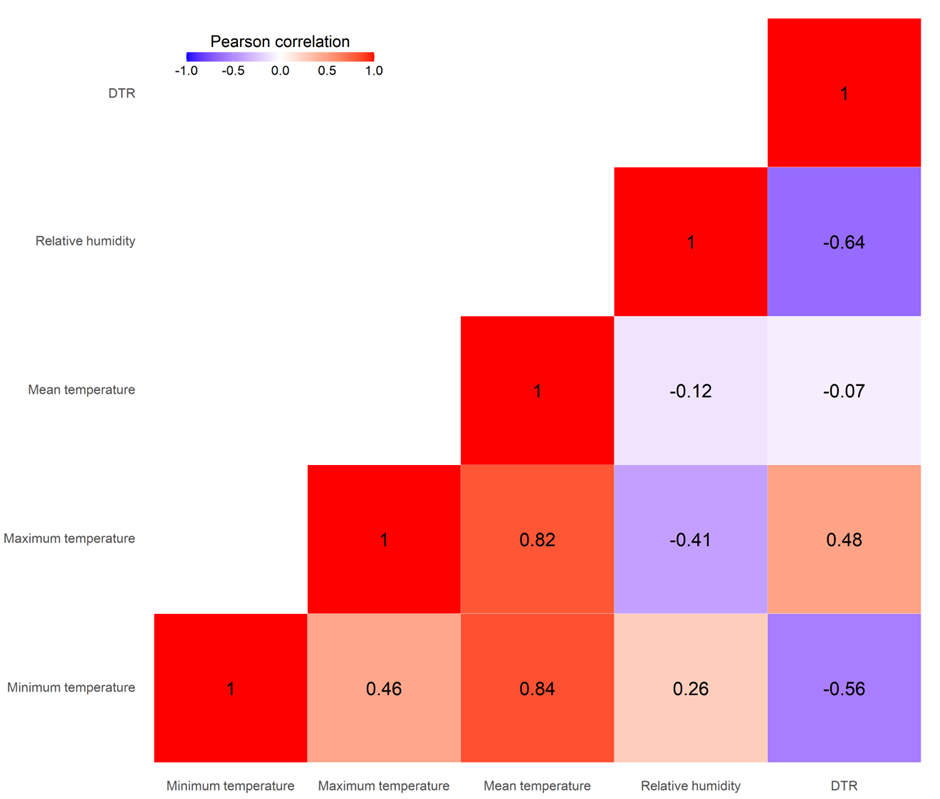
**Fig. S6** The Pearson correlation coefficients among independent variables


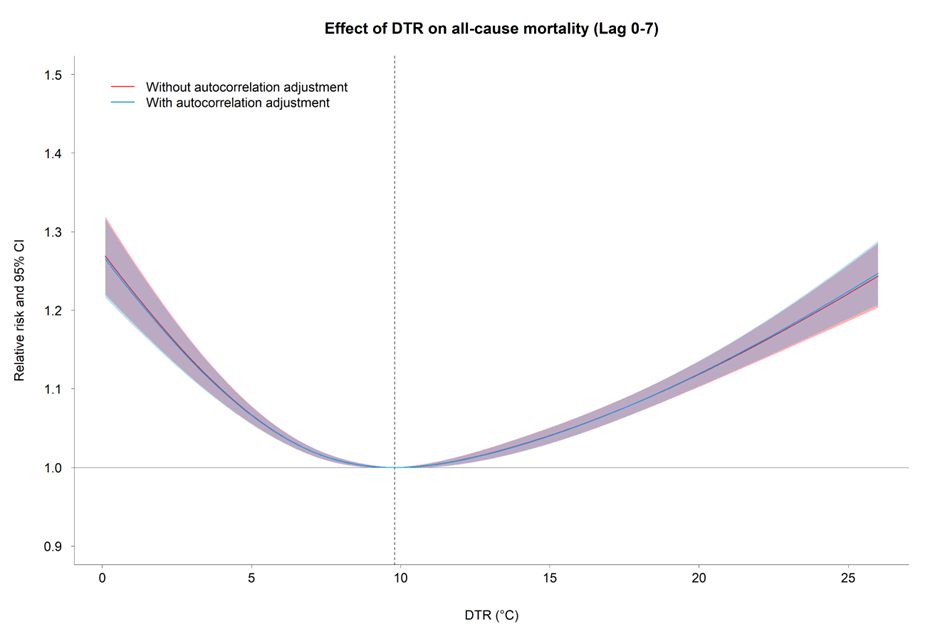


**Fig. S7** The curve of DTR-mortality association at the national-level obtained from the model with and without adjustment for autocorrelation at cumulative lag 0-7 day. A vertical dotted line is the minimum mortality DTR (MM-DTR) (9.8°C)


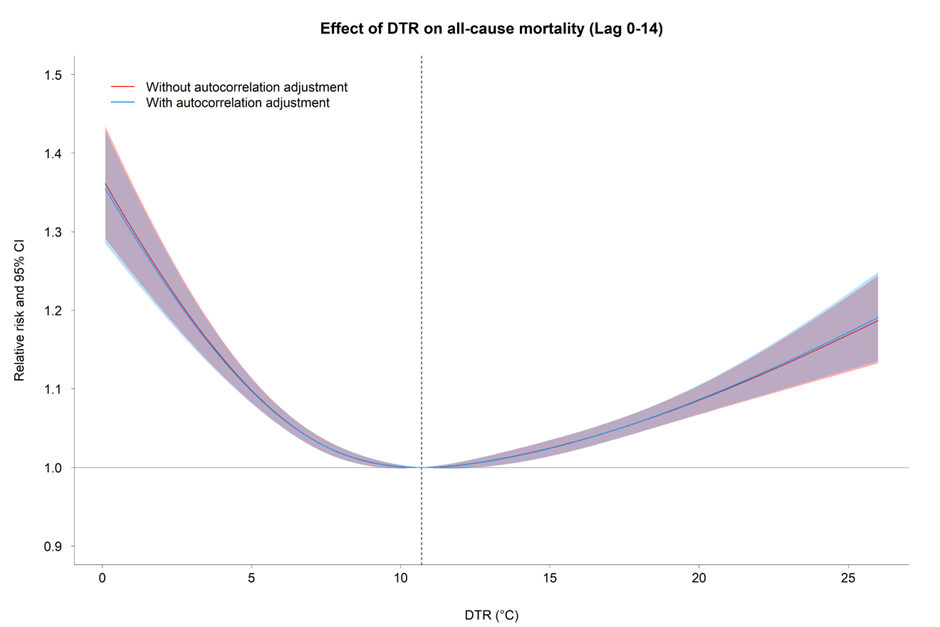


**Fig. S8** The curve of DTR-mortality association at the national-level obtained from the model with and without adjustment for autocorrelation at cumulative lag 0-14 day. A vertical dotted line is the minimum mortality DTR (MM-DTR) (10.7°C)

**
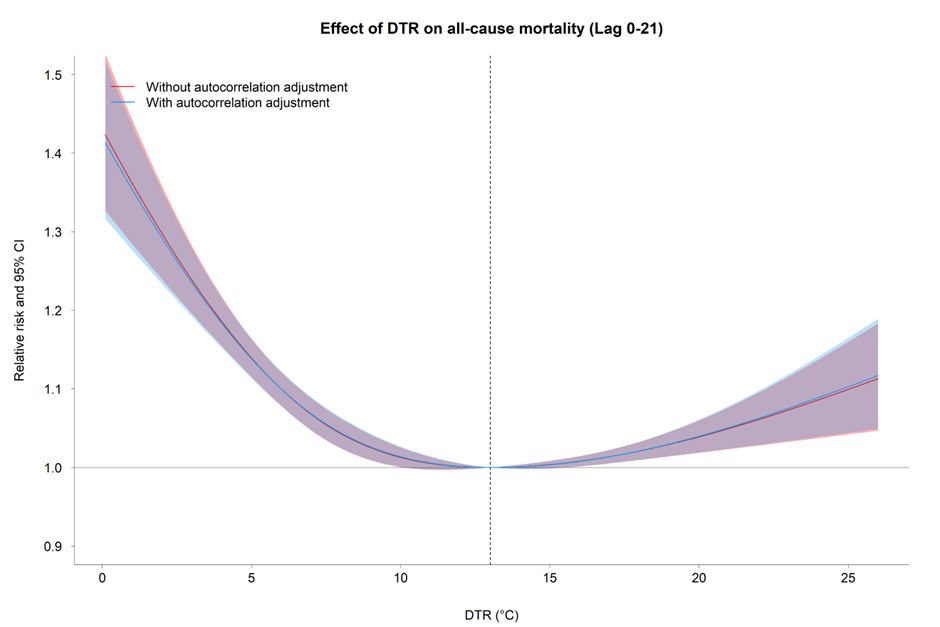
Fig. S9** The curve of DTR-mortality association at the national-level obtained from the model with and without adjustment for autocorrelation at cumulative lag 0-21 day. A vertical dotted line is the minimum mortality DTR (MM-DTR) (13.0°C)


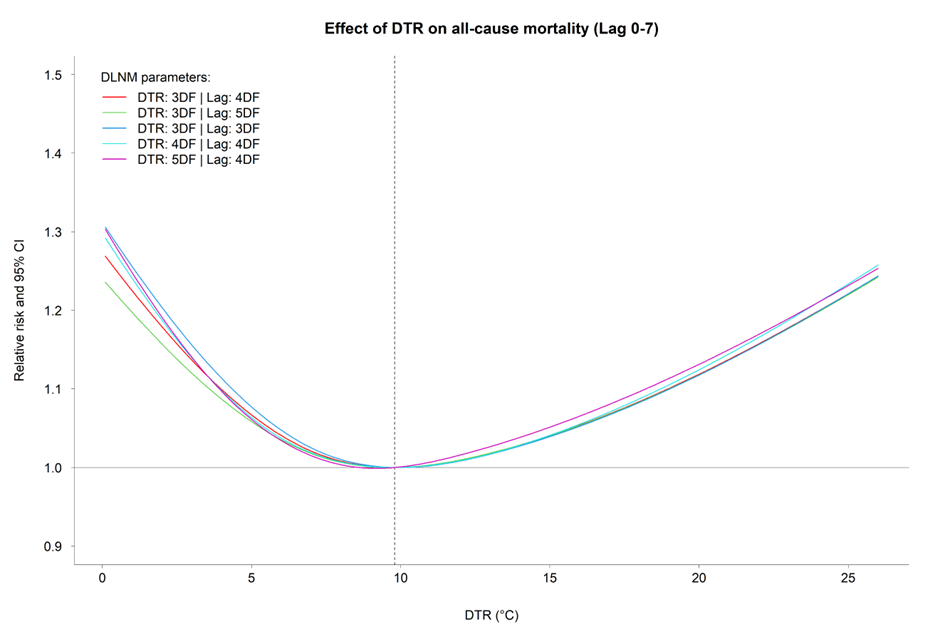


**Fig. S10** The curve of DTR-mortality association at the national-level, adjusted for different DFs of the *ns* function for the DTR variable and its lag in the cross-basis matrix at the cumulative lag 0-7 day. A vertical dotted line is the minimum mortality DTR (MM-DTR) (9.8°C)


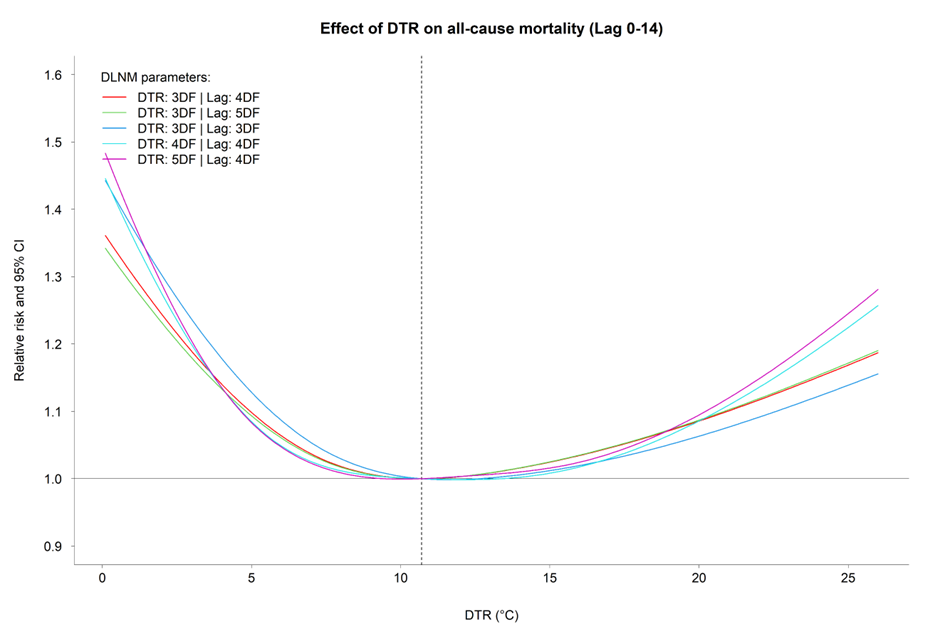


**Fig. S11** The curve of DTR-mortality association at the national-level, adjusted for different DFs of the *ns* function for the DTR variable and its lag in the cross-basis matrix at the cumulative lag 0-14 day. A vertical dotted line is the minimum mortality DTR (MM-DTR) (10.7°C)


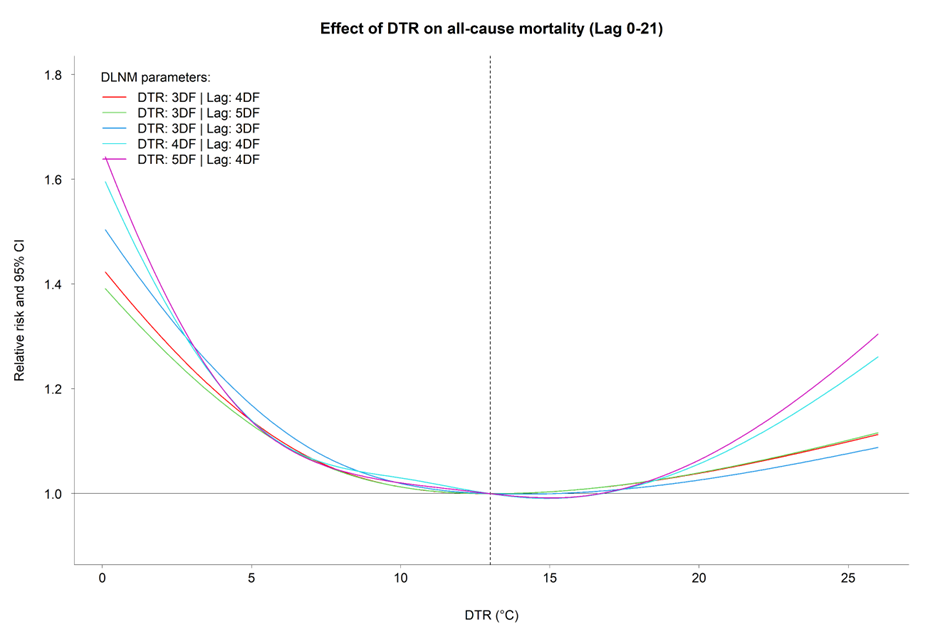


**Fig. S12** The curve of DTR-mortality association at the national-level, adjusted for different DFs of the *ns* function for the DTR variable and its lag in the cross-basis matrix at the cumulative lag 0-21 day. A vertical dotted line is the minimum mortality DTR (MM-DTR) (13.0°C)
